# Boolean network and meshless simulations for the comparison of transport and reaction mechanisms arising in one-short tri-exponential and uniform infusion electrochemotherapeutic treatments

**DOI:** 10.3389/fbinf.2026.1719700

**Published:** 2026-03-25

**Authors:** Fabián Mauricio Vélez Salazar, Iván David Patiño Arcila, Ismael E. Rivera Madrid, Marlon Rincón Fulla

**Affiliations:** 1 Grupo de Investigación e Innovación Ambiental (GIIAM), Institución Universitaria Pascual Bravo, Medellín, Colombia; 2 Institución Universitaria Pascual Bravo, Medellín, Colombia; 3 Grupo en Modelación Computacional de Sistemas Mecánico-Cuánticos, Universidad Nacional de Colombia, Medellín, Colombia

**Keywords:** Boolean modeling, electroporation, infusion, meshless techniques, tri-exponential, one-short, uniform infusion

## Abstract

Drug administration via the bloodstream involves some transport and reaction mechanisms ( RTMs ), such as extravasation, perfusion along blood vessels, transmembrane and interstitial transport, protein dissociation and association, and lymphatic drainage. These RTMs can be influenced by the type of pharmacokinetic ( PK ) profile used for drug delivery in the circulatory system, as well as by the bloodstream velocity $\left(\lambda_{inl}\right)$ . In electroporated tissues, the electric field magnitude ( E ) can also affect the RTMs because it brings about vessel vasoconstriction, cell membrane and vessel wall electro-permeabilization, and changes in tissue porosity. In the present work, in-house computational tools are employed to examine how the combination of E and $\lambda_{inl}$ influences the $RTM^{\prime }s$ existence, interaction, and rates arising in electrochemotherapy for two different PKs : One-short tri-exponential ( TPK ), where the drug concentration decreases exponentially after a one-short infusion, and one uniform ( UPK ), where the drug concentration is kept constant during the whole treatment. First, the ratios between extracellular, free intracellular, and bound intracellular concentrations are obtained from numerical simulations with a meshless code previously developed, calibrated, and validated. Subsequently, the interaction between the RTMs is investigated by means of a Boolean model presented here that is based on the comparison of the spatio-temporal evolution of the concentration ratios. Several combinations of E ( 0 kV/m ; 46 kV/m ; 70 kV/m ), $\lambda_{inl}$ ( $1\times 10^{-4}m/s$ ; $1\times 10^{-3}m/s$ ; $1\times 10^{-2}m/s$ ), and PK ( TPK and UPK ) are tested. The *in silico* findings indicate that $RTM^{\prime }s$ existence, interaction, and rates can vary between the two PKs ( TPK and UPK ) for a specific permutation of E and $\lambda_{inl}$ . Nevertheless, common features are identified between these pharmacokinetic profiles. In general, the lower E , the more uniform the transmembrane transport in the radial and axial direction; the decrease of $\lambda_{inl}$ also improves the radial homogeneity of this transport mechanism but negatively influences the axial uniformity. The uniformity of the mechanisms of association and dissociation is only altered monotonously by E (Vélez Salazar and Patiño Arcila, 2025).

## 1 Introduction

**FIGURE 1:**
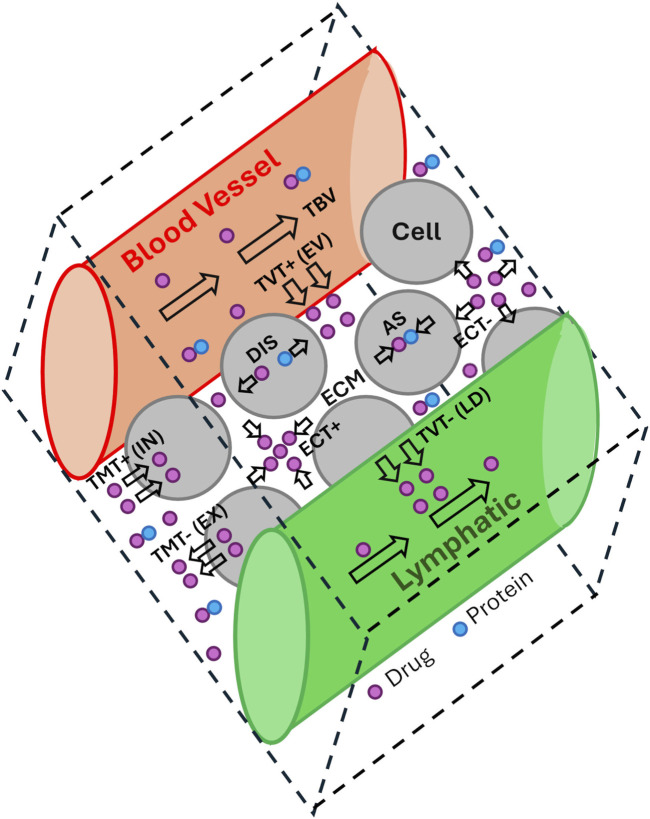
Mechanism of transportation reaction in electrochemotherapy (Vélez Salazar and Patiño Arcila, 2025).

The effectiveness of the administration of chemotherapeutic drugs by the circulatory system relies heavily on the blood perfusion rate and pharmacokinetic profile. Although this systemic delivery of a drug may have some drawbacks in terms of potential adverse effects on healthy tissues, it remains the predominant mechanism for delivering drugs to cancer tissues due to its ability to enhance the distribution of the drug through the microvasculature of the tumor (Vélez Salazar and Patiño Arcila, 2025; Dewhirst and Secomb, 2017). Electroporation has emerged as a viable procedure that allows for the delivery of chemotherapeutic drugs to specific tissue sections and thereby identifies the localized cytotoxic effects of the therapy (Vélez Salazar and Patiño Arcila, 2025). Optimal selection of electroporation parameters, including form of pulse, length, spacing, frequency, and voltage level, as well as the duration and number of electroporation therapies and the position of electrodes, is essential for successfully achieving the principal goal of reversible electroporation ( RE ), that is, to increase the permeability of the cellular membrane and enable the drug passage inside the cell space, while ensuring cellular viability (Vélez Salazar and Patiño Arcila, 2025; Markelc et al., 2017; Markelc et al., 2018; Granot and Rubinsky, 2008). In addition, the application of high-voltage, short-duration electric pulses can cause changes in the endothelial permeability and vessel diameter, which in turn can affect the motion of a drug along the vessels. This is especially important in distal and/or fenestrated vessels of a tumor (Vélez Salazar and Patiño Arcila, 2025; Markelc et al., 2017; Bellard et al., 2012; Brinton et al., 2018). As discussed by Vélez Salazar and Patiño Arcila (2023a), the bloodstream velocity and pharmacokinetic profile have a significant impact on the temporal and spatial changes of drug concentrations in vasoconstricted and electroporated tissues (Vélez Salazar and Patiño Arcila, 2025). This includes the extracellular space drug concentrations, as well as the free and bound drug concentrations within the cells. The changes in drug concentration can also alter the mechanisms of reaction and transport from the circulatory system on the way to the cell. The following mechanisms can be summarized (Figure 1).

### 1.1 Transport through the blood vessels (TBV)

Chemotherapeutic medications are administered systematically through the main circulatory system, allowing the drugs to reach the tumor microvasculature. The convection is the main method by which drugs are transported along tumor vessels. The concentration of drug within the vessels is influenced by different factors, counting the microvascular network structure (Winkler et al., 2004; Jain, 2005; Secomb et al., 1993; Vélez Salazar et al., 2025), intravascular pressure (Pries et al., 1998), shear stress vessel walls (Pries et al., 1998), leakage flow rate across the vessel walls (Vélez Salazar and Patiño Arcila, 2023a; Vélez Salazar et al., 2025; Vélez Salazar and Patiño Arcila, 2024; Vélez Salazar and Patiño Arcila, 2023b), initial dose (Hubbard et al., 2017), binding of proteins (Hubbard et al., 2017; Boyd and Becker, 2016), vasoconstriction and recovery of vessel diameter (Brinton et al., 2018; Vélez Salazar et al., 2025; Palanker et al., 2008; Mandel et al., 2013; Corovic et al., 2015; Markelc et al., 2013; Serša et al., 2017), factor of vascular endothelial growth (Vélez Salazar et al., 2025; Dewhirst and Ashcraft, 2016), tortuosity of the vessel (Sevick and Jain, 1989), acidosis and blood hypoxia (Dewhirst et al., 1992; Dewhirst et al., 1999; Kavanagh et al., 1993), anatomical and/or functional shunts presence (Sorg et al., 2008; Yeh et al., 2015), drug pharmacokinetic profile ( PK ) (Vélez Salazar et al., 2025; Hubbard et al., 2017), and bloodstream velocity ( $\lambda_{inl}$ ) (Bellard et al., 2012; Mandel et al., 2013; Lv et al., 2011), among others. A parametric study is carried out in this work to examine the effects of PK and $\lambda_{inl}$ on electrically stimulated blood vessels. The transport of fluid within blood vessels is represented in this study with an advection/convection equation. This equation is derived from a well-established model for the motion of fluid through circular porous tubes and a previous model presented in Vélez Salazar and Patiño Arcila (2023a), which accounts for vasoconstriction of the vessel and subsequent recovery (Vélez Salazar and Patiño Arcila, 2025).

### 1.2 Transvascular transport (TVT)

Transvascular transport is the motion of a drug from the blood vessel on the way to the space between the cells of the tissue (extravasation) or in the reverse direction (lymphatic drainage) (Vélez Salazar and Patiño Arcila, 2025). Both passive diffusion and convection are present in both cases, whereas the transcytosis mechanism is generally not considered. To analyze the transport of substances through the vessel wall considering convection and diffusion separately, the Staverman–Kedem–Katchalsky equation can be used in combination with Starling’s law (Jain, 1987; Zhan et al., 2014). These equations are formulated based on the hydraulic conductivity, diffusional permeability, osmotic reflection coefficient of the blood and lymphatic vessel walls, differences in osmotic and hydrostatic pressure between plasma and interstitial space, and intra-lymphatic pressure, among other factors. A more simplified approach involves using a Fickian diffusion equation that includes efficient permeability. This approach is applicable when the transvascular movement is primarily ruled by the gradient of concentration of substance instead of the pressure difference across the vessel wall (Vélez Salazar and Patiño Arcila, 2025; Hubbard et al., 2017; Eikenberry, 2009; Groh et al., 2014). In this study, transvascular transportation is referred to as EV or TVT+ when it happens from the vascular system to the interstitial compartment (extravasation) and as LD or TVT− when it happens in the contrary way (discharge of lymphatic fluid). Previous experimental studies (Vélez Salazar and Patiño Arcila, 2025; Markelc et al., 2018; Corovic et al., 2015; Kodama et al., 2019) have demonstrated that the application of electric pulses leads to the permeabilization of the endothelial cell membrane and the disruption of cell-to-cell junctions. This facilitates the TVT and can ultimately enhance the effectiveness of the therapy.

### 1.3 Extracellular transport (ECT)

This process depends on diffusion and convection mechanisms inside the extracellular space, where the interactions between the drug and proteins can hinder the drug transportation and affect the drug absorption into the cells. It is important to realize that the extracellular porosity and microvascular network might be highly nonhomogeneous, especially when healthy tissues and tumors are intermingled. At the macroscopic scale, it is necessary to consider equations of mass conservation, species transport in interstitial space, and fluid flow momentum. These equations incorporate different terms to account for the source (vascular extravasation) and sink (lymphatic drainage) of mass, flow resistance within the tissue (Darcian resistivity), influx/efflux effects, and association/dissociation of proteins. Conversely, they do not explicitly represent the vasculature of the tumor (Zhan et al., 2014; Goodman et al., 2008; Tzafriri et al., 2005; Arifin et al., 2009). However, the incorporation of a complex blood vessel network at a very small scale represents a significant computing challenge (Cattaneo and Zunino, 2014; Secomb, 2016; Stamatelos et al., 2014; Sefidgar et al., 2015; Stéphanou et al., 2005). The tumor cord approach, in contrast, provides a simplified and idealized representation of the tumor’s vascular structures, allowing for the evaluation of how a drug penetrates from a blood vessel to the less well-perfused areas inside the tumor (Vélez Salazar and Patiño Arcila, 2023a; Vélez Salazar et al., 2025; Vélez Salazar and Patiño Arcila, 2024; Vélez Salazar and Patiño Arcila, 2023b; Hubbard et al., 2017; Eikenberry, 2009; Groh et al., 2014). A model of tumor cord modified that includes a convective element to represent the tissue dimension variation when the constriction of the blood vessels happens was employed by Vélez Salazar and Patiño Arcila (2025) and Vélez Salazar and Patiño Arcila (2023a), and it is included in this investigation. In the current study, the term ECT+ signifies a positive net transport outside of cells in the representative unitary cell ( RUC ), reflecting an increase in the extracellular concentration $\left(C_{1}\right)$ (Vélez Salazar and Patiño Arcila, 2025) between two time instants. Conversely, ECT− denotes a negative net transport outside of cells, indicating a reduction in $C_{1}$ at a given RUC . Prior studies have investigated the effects of applying electric pulses on ECT , finding that the electrical stimulation can enhance the diffusion of nanoparticles (Kodama et al., 2019), chemotherapeutic drugs (Gibot et al., 2013; Zhang et al., 2014; Figini et al., 2025), and DNA (Sachdev et al., 2022) by reducing the interstitial barrier of extracellular matrix ( ECM ) (Vélez Salazar et al., 2025). The equilibrium between cell membrane and the extracellular space electro-permeabilization has been studied both *in vitro* (Sukharev et al., 1992; Pavšelj and Préat, 2005) and *in vivo* (Satkauskas et al., 2002; Bureau et al., 2000) using a mix of low voltage, long pulses and high voltage, short pulses (Vélez Salazar et al., 2025).

### 1.4 Transmembrane transport (TMT)

Transmembrane transport is the process by which a drug enters and exits the cells. These processes can happen simultaneously and involve four specific mechanisms of transport: (1) passive diffusion that occurs because of differences in concentration gradients, (2) facilitation of transport by protein channels with gates, (3) endocytosis–exocytosis, and (4) using carrier proteins for active motion that require metabolic energy from ATP or alternative sources. In this study, the tumor cord method integrates transport through the membrane by incorporating source/sink factors into the species transfer equation for the concentration outside of cells ( $C_{1}$ ) and free concentration inside the cells ( $C_{2}$ ) (Vélez Salazar and Patiño Arcila, 2025). The equations also consider the cell membrane permeability ( $K_{1}$ ), which is altered by a function called reversible electroporation degree ( ${DOE}_{R}$ ) (Vélez Salazar and Patiño Arcila, 2025) suggested by Boyd and Becker (2015). This work is focused on the net rate of transmembrane transport, taking into account that drug influx and efflux can happen simultaneously within the same RUC . Within this framework, the terms TMT+ or IN refer to the process of drug internalization, whereas TMT− or EX describe the process of drug externalization. The effect of electroporation ( EP ) on transportation through the membrane has been examined in numerous experimental investigations (Pavšelj and Préat, 2005; Batista Napotnik and Miklavčič, 2018; Kotnik et al., 2019; Rems and Miklavčič, 2016; Sweeney et al., 2018a), as well as in computational simulations (Vélez Salazar and Patiño Arcila, 2024; Secomb, 2016; Sefidgar et al., 2015; Boyd and Becker, 2015; Sweeney et al., 2018b; Argus et al., 2017; Šel et al., 2005). The main factors that contribute to the electro-permeabilization of the cell membrane, as explained by Sachdev et al. (2022), are the formation of hydrophilic pores in the lipid bilayers, changes in the structure of cell membrane caused by chemical modifications of lipid tails, and the denaturation of membrane proteins (Hubbard et al., 2017) leading to the creation of voltage gated ion-channels. Some macromolecules, like DNA, may undergo translocation toward the interior of the cell by initially aggregating on the cell surface (Faurie et al., 2010; Escoffre et al., 2011). A suitable control of the cell membrane integrity is crucial when applying electric pulses, as irreversible electroporation ( IRE ) can occur. During IRE , a fraction of the cell membrane pores remains unsealed after the electric pulse application, leading to the breakdown of certain regions and subsequently to chemical imbalance (Kramar et al., 2009). These actions can result in adverse effects on the tissue, including hemorrhage, portal vein thrombosis, bile duct damage, and infection (Kingham et al., 2012; Philips et al., 2013).

Most *in silico* studies that simulate transmembrane transport ( TMT ) at both macroscopic and microscopic levels have employed traditional numerical techniques such as the finite element method ( FEM ) and the finite volume method ( FVM ). FVM was employed in previous works by Boyd and Becker (2016), Boyd and Becker (2015), and Argus et al. (2017) to investigate the effects of level of voltage, pulse spacing, electrodes arrangement, and concentration of drug on the effectiveness and cell viability of treatments with electrochemotherapy (Hubbard et al., 2017; Vélez Salazar et al., 2022). Šel et al. (2005) utilized the finite element method ( FEM ) to compute the electric field distribution and tissue conductivity during electroporation. They confirmed the accuracy of their results by comparing them with *in vivo* measurements conducted on rabbit liver tissues. Argus et al. (2017) also employed the FEM to examine the increase of permeability of cellular membrane in mice hypodermic tumors (Šel et al., 2005). In Boyd and Becker (2015), Shirakashi et al. (2004), and Vélez Salazar et al. (2020), the finite element method ( FEM ) was utilized to analyze the electro-permeabilization in mouse skin tissues. This study considered several electrode configurations and pulse protocols used in *in vivo* research. Commercial software has also been used to simulate cell membrane permeabilization (Shirakashi et al., 2004). Recently, the global method of approximate particular solutions ( GMAPS ) was employed to investigate the spatial and temporal evolution of drug concentration in tissues that have been electroporated. The study conducted by Vélez Salazar et al. (2020) used this approach to examine the influence of voltage level ( V ) and pulse spacing ( $d_{pulses}$ ) on both the amount and uniformity of the internalized medicament. In addition, Vélez Salazar et al. (2022) analyzed the impact of two medications used in chemotherapy (cisplatin and doxorubicin) on transmembrane transportation. In their research, Puc et al. (2003) investigated the effect of elapsed time, pulse duration, and voltage level regarding drug transportation through the membrane (Hubbard et al., 2017). They developed mathematical equations for the effective coefficient of mass transfer among intracellular and extracellular areas using a model of two compartments (Vélez Salazar et al., 2025). Recent advancements in meshless methods for solving fractional and anomalous transport models in biomedical and environmental contexts further support the applicability of such computational approaches. For instance, Ahmad et al. (2023) developed a three-dimensional multi-term fractional model for solute transport in groundwater using local meshless techniques, demonstrating their efficacy in handling complex transport phenomena. Similarly, Ahmad et al. (2020) applied meshless strategies to simulate anomalous solute transport, while Li et al. (2020) and Wang et al. (2021) extended these methods to time-fractional PDEs and viscous wave equations, respectively. These studies highlight the flexibility and accuracy of meshless methods in modeling multi-scale, multi-physics transport systems.

### 1.5 Intracellular transport (ICT)

Intracellular transport is the mechanism that allows macromolecules to move throughout the cell membrane to enter the cell nucleus. The cytoplasm is a densely packed fluid containing a complex cytoskeleton network, many organelles in cells, and many proteins. In this situation, the convective and diffusive transport usually occur in the intracellular space (Hubbard et al., 2017; Figini et al., 2025). However, in the environment inside the cells, the drug dissociation ( DIS ) and association ( AS ) have a more crucial impact on the free intracellular concentration ( $C_{2}$ ) and bound intracellular concentration ( $C_{3}$ ). In the present study, the tumor cord model incorporates these reaction mechanisms into the equations of transport of species as source/sink terms, which in turn depend on the dissociation ( $k_{-2}$ ) and association ( $k_{2}$ ) rates. The current work does not consider the influence of electric pulse applications on the values of $k_{2}$ and $k_{-2}$ (Sefidgar et al., 2015).

The processes connected with drug motion from bloodstream to tumor cells are highly complex, and this complexity can be further increased by applying electric pulses (Vélez Salazar and Patiño Arcila, 2025; Sefidgar et al., 2015). Consequently, there is a rising need for computational tools to enhance understanding of the interaction among transport and reaction mechanisms involved in treatments using electrochemotherapy, and how this affects the space and time variations of drug concentration inside the tissue. The current work compares the transport and reaction mechanisms arising in electrochemotherapy under two drug infusion conditions that differ in the behavior of drug concentration within the bloodstream $\left(C_{v}\right)$ (Sefidgar et al., 2015): (1) a one-short tri-exponential ( TPK ) infusion where $C_{v}$ is a function of time, and (2) a uniform infusion ( UPK ) where $C_{v}$ is constant during the whole treatment. The use of an electric pulse with E=0 kV/m (electroporation absence), E=46 kV/m (limit of reversibility), and E=70 kV/m (limit of irreversibility), as well as the modification of the inlet blood velocities, namely, $\lambda_{inl}=\left[1\times 10^{-4}m/s,1\times 10^{-3}m/s,1\times 10^{-2}m/s\right]$ , are considered here as well. The reference values of $\lambda_{inl}$ were obtained from Hubbard et al. (2017). First, the $C_{2}/C_{1}$ , $C_{3}/C_{1}$ , and $C_{3}/C_{2}$ ratios are determined using a previously built, verified, and confirmed *in silico* instrument that utilizes GMAPS to resolve the main equations (Vélez Salazar and Patiño Arcila, 2025; Sefidgar et al., 2015). Then, principles of logical inference are utilized to establish links among the aforementioned mechanisms ( EV or TVT+ , TBV , ECT+ , LD *or* TVT− , IN or TMT+ , ECT− , EX or TMT− , DIS , and AS ) (Vélez Salazar and Patiño Arcila, 2025). The primary aim of this study is to explore the impact of the change of E and $\lambda_{inl}$ on the interaction between these mechanisms for one- short tri-exponential ( TPK ) and uniform infusions ( TPK and UPK , respectively) and to propose potential clinical consequences of this.

## 2 Governing equations and simulation techniques

**FIGURE 2:**
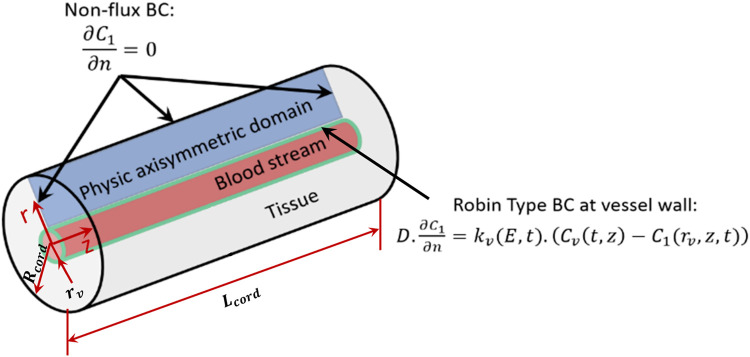
Physical domain border conditions.

This study utilizes a continuum, axisymmetric, tumor cord model for the transfer of species (see Figure 2). Typically, this model is utilized in steady-state domains (Vélez Salazar and Patiño Arcila, 2025; Dewhirst et al., 1999; Sorg et al., 2008; Puc et al., 2003). In a recent investigation (Vélez Salazar and Patiño Arcila, 2023a), the governing equation of interstitial area was altered by integrating a term of divergence that considers advective transport due to the tissue contraction and expansion because of the constriction of blood vessels. The Supplementary Section of Vélez Salazar and Patiño Arcila (2023a) contains a deduction of the modified model. The revised tumor cord equations are expressed as follows (Hubbard et al., 2017):

$$\delta_{1}\frac{\partial C_{1}}{\partial t}=D\nabla^{2}C_{1}-\nabla C_{1}\cdot \vec{u}+\alpha .k_{1}\;\left(E,t\right).\left(C_{2}-C_{1}\right),$$ (1)

$$\delta_{2}\frac{\partial C_{2}}{\partial t}=\alpha .k_{1}\left(E,t\right).\left(C_{1}-C_{2}\right)-\delta_{2}k_{2}C_{2}\left(C_{o}-C_{3}\right)+\delta_{2}k_{-2}C_{3},$$ (2)

$$\delta_{2}\frac{\partial C_{3}}{\partial t}=\delta_{2}k_{2}C_{2}\left(C_{o}-C_{3}\right)-\delta_{2}k_{-2}C_{3},$$ (3)

where the field variables include extracellular drug concentration ( $C_{1}$ ), free intracellular drug concentration ( $C_{2}$ ), and bound intracellular drug concentration ( $C_{3}$ ). Variables $\delta_{1},\delta_{2},$ and α stand for the extracellular volume content, intracellular volume content, and total area of the surface ratio of cellular membrane to the volume of the cord domain, which correspond to geometric-dependent properties (Vélez Salazar and Patiño Arcila, 2025). D and $k_{1}$ are the coefficient of interstitial diffusion and permeability of cell membrane corresponding to transport properties that rely on time and the electric field strength. Conversely, reaction properties are the rate of drug dissociation ( $k_{-2}$ ) and association ( $k_{2}$ ). The effective diffusivity, D , is contingent upon the extracellular porosity, $\delta_{1}$ , and may be expressed as $D=D_{0}.$ $\left(\delta_{1}/\delta_{1,0}\right)$ when the temporal variation of porosity is small, with $\delta_{1,0}$ and $D_{0}$ as the porosity and the coefficient of diffusion corresponding to the initial cord domain (in the absence of electroporation) (Vélez Salazar and Patiño Arcila, 2025). The tissue velocity field resulting from the constriction of blood vessels and subsequent vessel radius recovery is presumed to possess solely radial components, specifically, $\vec{u}=\left(u_{r},0\right)$ . Figure 2 illustrates the boundary conditions, which are Robin-type on the edge corresponding to the vessel wall, represented by the subsequent equation:

$$D.\frac{\partial C_{1}}{\partial n}=k_{v}.\left(C_{v}\left(t,z\right)-C_{1}\left(r_{v},z,t\right)\right),$$ (4)

where $r_{v}$ denotes the radius of the vessel influenced by vasoconstriction, $k_{v}\left(E,t\right)$ represents the vessel wall permeability, $C_{v}\left(t,z\right)$ indicates the drug concentration in the bloodstream as a function of time and space, $C_{1}\left(r_{v},z,t\right)$ signifies the extracellular drug concentration at the interface between the vessel and tissue, and $\partial C_{1}/\partial n$ refers to $C_{1}$ normal gradient (Vélez Salazar and Patiño Arcila, 2025). The remaining boundaries are subject to a non-flux condition.

A prior work specified a complete explanation of how the application of electric pulses impacts the radius of the vessel ( $r_{v}$ ), the permeability of the wall ( $k_{v}$ ), the coefficient of interstitial diffusion ( D ), cellular membrane permeability ( $k_{1}$ ), and the properties that depend on the tissue geometry ( $\delta_{1},\delta_{2}$ and α ) (Vélez Salazar and Patiño Arcila, 2025; Vélez Salazar and Patiño Arcila, 2023b). In conclusion, the following was implemented:

- For electro-permeabilization of the cellular membrane and the vessel wall, a transient electroporation degree function $\left({DOE}_{R}\right)$ is taken into account in the Equation 5 (Vélez Salazar and Patiño Arcila, 2025; Boyd and Becker, 2016; Sweeney et al., 2018b):

$${DOE}_{R}\left(E,t_{p}\right)=DOE\left(E\right).\left[\left(1-DIE\left(E\right)\right).e^{-\frac{t_{p}}{\tau_{i}}}+DIE\left(E\right)\right].$$ (5)

Utilizing the functions depending on the strength of the electric field, $DIE\left(E\right)$ and $DOE\left(E\right)$ , as provided by Equations 6, 7:

$$DOE\left(E\right)=\frac{\sigma \left(E\right)-\sigma_{\min}}{\sigma_{\max}-\sigma_{\min}},$$ (6)

$$DIE\left(E\right)=\left\{\begin{array}{l} 0,when\;E_{irrev}>E,\\ \frac{\sigma \left(E\right)-\sigma_{irrev}}{\sigma_{\max}-\sigma_{irrev}},when\;E_{irrev}\leq E, \end{array}\right.$$ (7)

where $E,E_{irrev},t_{p}$ , and $\tau_{i}$ represent the electric field strength, the irreversible limit of the field magnitude, the duration since the administration of the last pulse, and the permeability decay temporal coefficient, whereas $\sigma_{\max},\sigma_{\min}$ , $\sigma_{irrev}$ , and $\sigma \left(E\right)$ are the maximum, minimum, irreversible, and the effective electrical conductivity, respectively (Vélez Salazar and Patiño Arcila, 2025; Vélez Salazar and Patiño Arcila, 2023b; Hubbard et al., 2017). The effective electrical conductivity for a specific value of E can be calculated by Equation 8 employing a sigmoidal function (Vélez Salazar and Patiño Arcila, 2025; Hubbard et al., 2017; Argus et al., 2017):

$$\sigma \left(E\right)=\frac{\sigma_{\max}-\sigma_{\min}}{1+\alpha^{*}.e^{-\frac{E-a^{*}}{b^{*}}}}+\sigma_{\min},$$ (8)

where the parameters for fitting $a^{*}$ and $b^{*}$ are calculated using the limit of reversibility $\left(E_{rev}\right)$ and the limit of irreversibility $\left(E_{irrev}\right)$ of the electric field intensity in the Equations 9, 10:

$$a^{*}=\left(E_{rev}+E_{irrev}\right)/2,$$ (9)

$$b^{*}=\left(E_{irrev}-E_{rev}\right)/\beta^{*}.$$ (10)

The permeabilization states of the wall vessel and the cell membrane are determined by Equation 11

$$k_{i}\left(E,t_{p}\right)={DOE}_{R}\left(E,t_{p}\right).\left(k_{i,\max}-k_{i,\min}\right)+k_{i,\min},$$ (11)

where $k_{i,\max}$ and $k_{i,\min}$ are the highest and lowest values of permeability, with $i=\left[1,v\right]$ , where the subindex “1” refers to the cell membrane, while the subscript $\prime \prime v^{\prime \prime }$ indicates the wall of the vessel (Vélez Salazar and Patiño Arcila, 2025).

- The mathematical model for vessel vasoconstriction proposed by Vélez Salazar and Patiño Arcila (2023b) is examined, where the normalized radius of the vessel, $\hat{r}$ , defined as the ratio of the current vessel radius to the radius of vessel $\left(\hat{r}=r_{v}/r_{v0}\right)$ , is approximated using a function with two exponentials (Vélez Salazar and Patiño Arcila, 2025):

$$\hat{r}\left(E,t_{p}\right)=\left[\left(1-{\hat{r}}_{\min}\right).e^{-m_{r}.E.e^{-t_{p}/\tau_{r}}}+{\hat{r}}_{\min}\right],$$ (12)

where $\tau_{r}$ , $m_{r}$ , $\hat{r}$ , and ${\hat{r}}_{\min}$ stand for coefficient of decay time for vasoconstriction, coefficient of vasoconstriction, normalized ratio, and minimal normalized ratio, respectively.

- Assuming that the interface velocity function is not space-dependent, the opposite border continues unaltered, and that the motion of the tissue-vessel interface is uniform and happens in the radial direction, the radial velocity $\left(u_{r}\right)$ at any location inside the tissue may be calculated with the methodology employed by Vélez Salazar and Patiño Arcila (2025), Jackson (2003), Simpson et al. (2015), Landman et al. (2003), Baker et al. (2010), and Yates et al. (2012) for the migration of cells in shifting tissue spaces, finding the subsequent sentence (Vélez Salazar and Patiño Arcila, 2025; Hubbard et al., 2017):

$$u_{r}\left(r,E,t\right)=\left(\frac{r‐R_{\text{cord}}}{r_{v}‐R_{\text{cord}}}\right).\frac{dr_{v}\left(E,t\right)}{\mathrm{dt}},\text{for }r_{v}\leq r\leq R_{cord},$$ (13)

where r , $r_{v}$ , and $R_{cord}$ denote the radial coordinate, current radius of the vessel, and radius of cord, as well, whereas $dr_{v}/dt$ represents the interface speed, derived from the temporal derivative of Equation 12.

The GMAPS methodology presented by Vélez Salazar and Patiño Arcila (2023b), which is iterative, is applied in this study. The primary features of this strategy are:

- A fully implicit formulation is employed for temporal discretization.
- Factors of scale $S_{L}$ , $S_{T}$ , and $S_{C}$ are employed for space, time, and concentration to enhance the final component of conditioning.
- The Gauss–Seidel method, which is iterative, is employed to solve the fields of concentration ( $C_{1}$ , $C_{2}$ , and $C_{3}$ ) at every time instant, with a relative error threshold of $1\times 10^{-6}$ .
- Under-relaxation is utilized to modify the concentration field.
- A system of advection/convection is employed to determine the drug concentration within the circulatory system, $C_{v}$ , which is contingent upon the drug pharmacokinetic profile ( PK ), inlet vessel pressure $\left(p_{inl}\right)$ , vessel flow velocity $\left(\lambda \right)$ , viscosity of plasma $\left(\mu \right)$ , radius of the circulatory system $\left(r_{v}\right)$ , and the permeability coefficient of the vessel wall $\left(k_{vc}\right)$ (Vélez Salazar and Patiño Arcila, 2025). The formulation is expressed as

$$\frac{dC_{v}}{dt}=-\lambda \left(z\right)\frac{dC_{v}\left(z,t\right)}{dz}+k_{v}.\left(C_{1}\left(r_{v},z,t\right)-C_{v}\left(z,t\right)\right).$$ (14)

Equation 14 can be used to obtain a recurrence relationship for the drug concentration profile, $C_{v}$ , by applying non-dimensionalization, a temporal implicit scheme, and a backward scheme for the gradients. This is demonstrated by Vélez Salazar and Patiño Arcila (2025) and Vélez Salazar and Patiño Arcila (2023b).

- To minimize the computational cost, the vasoconstriction effect is not considered when the relative reduction of the vessel radius is above 90%. Alternatively, this effect is assessed by recalculating the physical space mesh, the GMAPS matrices that depend on geometry, along with the geometry-dependent tissue properties ( $\delta_{1}$ , $\delta_{2}$ , and α ). The fluid uptake through the vessel wall, which subsequently alters the velocity of blood inside the vessel $\left(\lambda \right)$ , is represented by a model for fluid passage throughout circular porous tubes (Vélez Salazar and Patiño Arcila, 2025; Yates, 2014). This model incorporates the vessel wall permeability coefficient $\left(k_{vc}\right)$ , plasma viscosity $\left(\mu \right)$ , inlet blood velocity $\left(\lambda_{inl}\right)$ , permeate pressure $\left(p_{p}\right)$ , inlet blood pressure $\left(p_{inl}\right)$ , and inlet flow rate $\left(Q_{inl}\right)$ . The plasma speed, $\lambda \left(z\right)$ , may be calculated using the subsequent equation:

$$\lambda \left(z\right)=\lambda_{inl}-\frac{Q_{leak}\left(z\right)}{\pi r_{v}^{2}},$$ (15)

where $\lambda_{inl}$ denotes the inlet blood velocity, and $Q_{leak}\left(z\right)$ represents the leakage flow rate through the vessel wall as calculated by Equation 16

$$Q_{leak}=2\pi r_{v}k_{vc.}\left[\left(p_{inl}-p_{p}\right)z-\frac{A^{*}}{\vartheta^{*}}\left(e^{\vartheta^{*}z}+e^{-\vartheta^{*}z}-2\right)-\left(p_{inl}-p_{p}\right)\left(z-\frac{e^{\vartheta^{*}z}-e^{-\vartheta^{*}z}}{2\vartheta^{*}}\right)\right].$$ (16)

With coefficients $A^{*}$ and $\vartheta^{*}$ defined by the subsequent mathematical formulae:

$$\vartheta^{*}=\sqrt{\frac{16\mu k_{vc}}{r_{v}^{3}}},$$ (17)

$$A^{*}=\frac{1}{2}\frac{8\mu Q_{inl}}{\pi r_{v}^{4}\vartheta^{*}}.$$ (18)

- The accuracy and convergence analysis of the GMAPS code utilized herein was conducted in a previous work of this research group (Vélez Salazar and Patiño Arcila, 2023a), and the chosen values for the number of collocation points ( $N_{points}$ ), under-relaxation factor ( ω ) and time step size ( Δt ) are utilized here as well. According to the numerical results obtained by Vélez Salazar and Patiño Arcila (2023a), $N_{points}=1111$ , Δt=3.5s , and ω=0.30 represent an adequate trade-off between accuracy and computational cost. The GMAPS algorithm was tested by Vélez Salazar and Patiño Arcila (2023a) against numerical findings previously reported by Groh et al. (2014) and Hubbard et al. (2017). In these studies, the problem represented by Equations 1–3 and Figure 2 was resolved employing a radially symmetric compartment approach and the finite volume method ( FVM ). The case of Groh et al. (2014) did not consider the drug variation along the bloodstream, and $L^{2}$ relative error norms of $1.36\times 10^{-3}$ for the extracellular concentration $\left(C_{1}\right)$ and $1.14\times 10^{-3}$ for the bound intracellular concentration $\left(C_{3}\right)$ were achieved. On the other hand, the case of Hubbard et al. (2017) took into account a bloodstream velocity of $\lambda_{inl}=1\times 10^{-3}m/s$ , leading to a longitudinal change of the drug concentration; for this case, the $L^{2}$ relative error norm for the bound intracellular drug exposure $\left(C_{3\exp}\right)$ was $3.66\times 10^{-3}$ . Details about accuracy and convergence analysis of GMAPS can be found elsewhere (Vélez Salazar and Patiño Arcila, 2023a).

**FIGURE 3:**
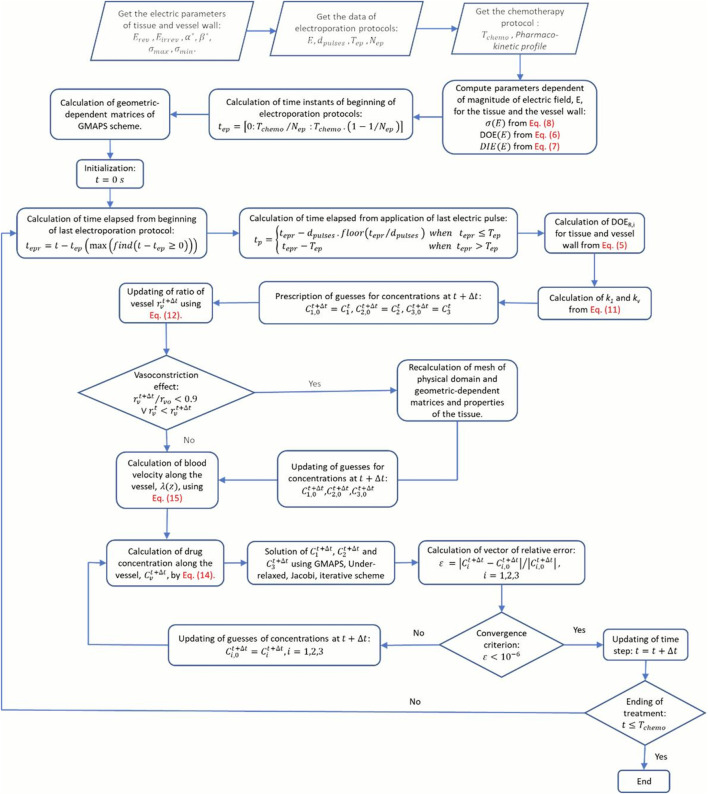
Streamlined flowchart of the global method of approximate particular solutions (GMAPS) framework.

Figure 3 illustrates a summarized flowchart of GMAPS utilized in the current investigation.

## 3 Boolean framework

Boolean modeling has been demonstrated as a powerful tool for analyzing the complex dynamics of biological systems (Karode, 2001; Schwab et al., 2020; Wang et al., 2012; Graudenzi et al., 2011). This instrument may be utilized in cancer therapies to clarify the relationships between the phenomena involved in the systemic delivery of drugs and to develop prospective electrochemotherapeutic protocols (Hubbard et al., 2017; Dahlhaus et al., 2016; Bozic et al., 2013; der Heyde et al., 2014; Fumiã and Martins, 2013). As previously indicated, many reaction and transport mechanisms are involved in the pharmacokinetics from the circulatory system to cells, and vice versa. The space and time change of the extracellular concentration ( $C_{1}$ ), free intracellular concentration ( $C_{2}$ ), and bound intracellular concentration ( $C_{3}$ ) is determined by the interaction among these mechanisms. Consequently, by analyzing the temporal and spatial dynamics of the $C_{3}/C_{2}$ , $C_{3}/C_{1}$ , and $C_{2}/C_{1}$ ratios, the relationships among these mechanisms ( EV or TVT+ , TBV , ECT+ , LD or TVT− , IN or TMT+ , ECT− , EX *or* TMT− , DIS , and AS ) can be revealed as the electrochemotherapeutic therapy progresses (Vélez Salazar and Patiño Arcila, 2025). Additionally, this interaction is influenced by the pharmacokinetic profile ( PK ), the velocity of inlet blood flow $\left(\lambda_{inl}\right)$ , and the electric field magnitude ( E ) (Vélez Salazar and Patiño Arcila, 2025).

### 3.1 Logical deductions regarding the evolution C_3_/C_2_, C_3_/C_1_ and C_2_/C_1_ ratios

For transmembrane transport governed by diffusion ( TMT ), medication transfer occurs across the plasma membrane and is mostly ruled by the concentration differential between $C_{1}$ and $C_{2}$ , as represented in Equations 1 and 2. Note that the bound intracellular drug is unable to exit to the extracellular region (Vélez Salazar and Patiño Arcila, 2025). In light of this, the TMT net rate at a specific temporal point and tissue RUC depends on $C_{2}/C_{1}$ ratio. A ratio less than 1 indicates that the gradient of drug concentration moves into the cell, leading to internalization of the drug. On the other hand, when $C_{2}/C_{1}$ is greater than 1, a contrary concentration differential happens, leading to externalization of drug. When the $C_{2}/C_{1}$ ratio is equal to 1, a partial equilibrium of species is achieved. Concerning the association of drug inside the cell, note that this process is limited by the concentration of binding sites, denoted as $C_{0}$ . Once the bound intracellular concentration, $C_{3}$ , reaches the value of $C_{0}$ , additional association of drug cannot occur. Table 1 displays the logical proposals and inferences for the $C_{2}/C_{1}$ and $C_{3}/C_{1}$ ratios, together with their corresponding physical significance.

**TABLE 1:** Logical deductions and consequences for $C_{3}/C_{1}$ and $C_{2}/C_{1}$ at any temporal points and for each location within the tissue domain.

Logical proposal		Logical consequence	
Declaration	Physical denotation	Declaration	Logical denotation
P_1_: $C_{2}/C_{1}<1$	The concentration gradient is into the cell	$P_{1}↔\exists IN$	P_1_ is both a sufficient and a necessary condition for drug IN existence
P_2_: $C_{2}/C_{1}>1$	The concentration gradient is out of the cell	$P_{2}↔\exists EX$	P_2_ is both a sufficient and a necessary condition for drug EX existence
P_3_: $C_{2}/C_{1}=1$	Partial balance of extracellular and free intracellular concentration	$P_{3}↔∄\;EX\wedge ∄\;IN$	P_3_ is both a sufficient and a necessary condition for the partial equilibrium between $C_{1}$ and $C_{2}$
P_4_: $C_{3}/C_{1}=C_{0}/C_{1}$	The concentration of binding sites, $C_{0},$ has been attained by $C_{3}$	$P_{4}\to ∄AS$	P_4_ is a sufficient condition for inhibiting the $C_{2}$ into $C_{3}$ association

The transport and reaction mechanisms significantly influence the spatio-temporal variations of $C_{1}$ , $C_{2}$ , and $C_{3}$ . Therefore, when there is extravasation ( EV ) or positive transvascular transport ( TVT+ ), the interstitial concentration, $C_{1}$ , increases at the interface between the vessel wall and tissue. Furthermore, the concentration $C_{1}$ increases in the interstitial space due to externalization ( EX ) or negative transmembrane transport ( TMT− ), as well as positive extracellular transport ( ECT+ ) (Vélez Salazar and Patiño Arcila, 2025; Vélez Salazar and Patiño Arcila, 2023b). As expected, $C_{1}$ shows a decrease as a result of the three contrary mechanisms: cell internalization ( IN ), lymphatic drainage ( LD ), and negative extracellular transport ( ECT− ). In contrast, the dissociation of the drug ( DIS ) and the internalization ( IN or TMT+ ) originate an increase of the free intracellular concentration ( $C_{2}$ ) (Vélez Salazar and Patiño Arcila, 2025). The procedures of externalization ( EX or TMT− ) and association of the drug ( AS ) exert opposing effects on $C_{2}$ . The bound intracellular concentration ( $C_{3}$ ) is only increased by the association of the drug ( AS ) and decreased by the dissociation of the drug ( DIS ). A concise overview of implications and logical statements in Table 2 explains how the mechanisms of transport and reaction affect the variations in $C_{1}$ , $C_{2}$ , and $C_{3}$ and their respective ratios ( $C_{2}/C_{1}$ , $C_{3}/C_{1}$ , and $C_{3}/C_{2}$ ). As reasonable, an increase in $C_{2}$ and a reduction in $C_{1}$ are sufficient but not necessary conditions for the increase of the $C_{2}/C_{1}$ ratio. The same principles can be utilized for the $C_{3}/C_{1}$ and $C_{3}/C_{2}$ ratios. Table 2 contains the logical propositions that compare $C_{1}$ , $C_{2}$ , and $C_{3}$ , as well as their corresponding ratios, at two specific time points referred to as $t_{i}$ and $t_{i+1}$ . It is crucial to highlight that within a representative unitary cell ( RUC ), various opposite mechanisms, such as drainage and extravasation, positive and negative extracellular transport, association and dissociation of drugs, and internalization and externalization, can occur concurrently. However, the primary mechanisms within each RUC are those examined in this study. From a Boolean perspective, LD (lymphatic drainage) and EV (extravasation), ECT− (negative outside-of-cells transport) and ECT+ (positive outside-of-cells transport), EX (externalization) and IN (internalization), and DIS (dissociation) and AS (association) are regarded as mutually exclusive mechanisms inside the RUC .

**TABLE 2:** Logical deductions and consequences for how the mechanisms of transport and reaction influence the concentration fields.

Logical proposition		Logical implication	
Declaration	Physical denotation	Declaration	Logical denotation
P_5_: $C_{1}\left(t_{i}\right)\leq C_{1}\left(t_{i+1}\right)$	Extracellular concentration increases or remains equal between times $t_{i}$ and $t_{i+1}$	$P_{10}\Delta P_{11}↔P_{5}$	P_10_ or P_11_ (but not both) is a sufficient and necessary condition for P_5_
P_6_: $C_{2}\left(t_{i}\right)\leq C_{2}\left(t_{i+1}\right)$	Free intracellular concentration increases or remains identical between times $t_{i}$ and $t_{i+1}$	$P_{8}\Delta P_{9}↔P_{6}$	P_8_ or P_9_ (but not both) is a sufficient and necessary condition for P_6_
P_7_: $C_{3}\left(t_{i}\right)\leq C_{3}\left(t_{i+1}\right)$	Bound intracellular concentration increases or remains identical between times $t_{i}$ and $t_{i+1}$	$P_{12}↔P_{7}$	P_12_ is a necessary and sufficient condition for P_7_
P_8_: IN≥AS	The rate of internalization is equal to or imposed on the rate of association	$P_{6}\wedge \neg P_{5}\to P_{14}$	$C_{2}$ increasing and $C_{1}$ decreasing between $t_{i}$ and $t_{i+1}$ is not a necessary but is a sufficient condition for an increase in the C_2_/C_1_ ratio.
P_9_: EX≤DIS	The rate of dissociation is equal to or imposed on the externalization rate	$P_{7}\wedge \neg P_{5}\to P_{15}$	$C_{3}$ increasing and $C_{1}$ decreasing between $t_{i}$ and $t_{i+1}$ is not a necessary but is a sufficient condition for an increase in the C_3_/C_1_ ratio
P_10_: EX≥ECT−	The rate of externalization is equal to or imposed upon the negative extracellular transport	$P_{7}\wedge \neg P_{6}\to P_{16}$	$C_{3}$ increasing and $C_{2}$ decreasing between $t_{i}$ and $t_{i+1}$ is not a necessary but is a sufficient condition for the ratio C_3_/C_2_ increase
P_11_: IN≤ECT+	Positive outer cell transport rate is equal to or imposed upon the degree of internalization.		
P_12_: ∃AS	Exists C_2_ association into C_3_		
P_13_: ∃DIS	Exists C_3_ dissociation into C_2_		
P_14_: $\frac{C_{2}}{C_{1}}\left(t_{i}\right)\leq \frac{C_{2}}{C_{1}}\left(t_{i+1}\right)$	The C_2_/C_1_ ratio increases or remains equal between time stages $t_{i}$ and $t_{i+1}$		
P_15_: $\frac{C_{3}}{C_{1}}\left(t_{i}\right)\leq \frac{C_{3}}{C_{1}}\left(t_{i+1}\right)$	The C_3_/C_1_ ratio increases or remains equal between time stages $t_{i}$ and $t_{i+1}$		
P_16_: $\frac{C_{3}}{C_{2}}\left(t_{i}\right)\leq \frac{C_{3}}{C_{2}}\left(t_{i+1}\right)$	The C_3_/C_2_ ratio increases or remains equal between time stages $t_{i}$ and $t_{i+1}$		

### 3.2 Logical deductions regarding C_2_/C_1_, C_3_/C_1_, and C_3_/C_2_ ratio interactions

This part applies the implication rules to deduce how the time behavior of $C_{2}/C_{1}$ , $C_{3}/C_{1}$ , and $C_{3}/C_{2}$ reveals the interaction of the mechanisms of transport and reaction ( RTMs ) associated with the delivery of the drug. For example, the logical declaration $P_{7}\wedge \neg P_{6}\to P_{16}$ shown in Table 2 indicates that the $C_{3}$ increase and $C_{2}$ decrease between time instants $t_{i}$ and $t_{i+1}$ is a sufficient but not necessary condition for the $C_{3}/C_{2}$ ratio to increase. By applying implication rules, this statement may be reformulated, thus:

$$P_{7}\wedge \neg P_{6}\to P_{16},$$ (19)

$$∴\neg P_{16}\to \neg \left(P_{7}\wedge \neg P_{6}\right),$$ (20)

through the application of the transposition law by the Equation 21,

$$∴\neg P_{16}\to \left(\neg P_{7}\vee P_{6}\right),$$ (21)

by principles of double negation

$$∴\neg P_{16}\to \left[\neg P_{12}\vee \left(P_{8}\Delta P_{9}\right)\right],$$ (22)

according to De Morgan’s second law and the constructive dilemma, as well as the biconditional and transposition laws, considering the declarations $P_{12}↔P_{7}$ and $P_{8}\Delta P_{9}↔P_{6}$ from Table 2.

The logical statement Equation 22 means that the decrease of the $C_{3}/C_{2}$ ratio implies the following: (1) if there is a drug association $\left(\neg P_{12}\right.$ is false), the rate of internalization is equal to or larger than the rate of association ( $P_{8}$ is true implying that $P_{9}$ is false; 2) if there is dissociation $\left(\neg P_{12}\right.$ is true), the remaining mechanisms may interact in different ways.

Analogous declarations can be derived from the temporal behavior of $C_{2}/C_{1}$ and $C_{3}/C_{1}$ . For example, regarding the temporal increase of these two concentration ratios ( $C_{2}/C_{1}$ and $C_{3}/C_{1}$ ) (Vélez Salazar and Patiño Arcila, 2025), identical analysis of inference for logical declarations $\neg P_{6}\wedge P_{5}\to \neg P_{14}$ and $\neg P_{7}\wedge P_{5}\to \neg P_{15}$ (see Table 2) is delineated in the subsequent terms:

$$P_{14}\to \left[\left(P_{8}\Delta P_{9}\right)\vee \neg \left(P_{10}\Delta P_{11}\right)\right],$$ (23)

$$P_{15}\to \left[P_{12}\vee \neg \left(P_{10}\Delta P_{11}\right)\right].$$ (24)

The concurrent presence of $P_{14}$ , $P_{15}$ , and $\neg P_{16}$ , which corresponds to the increase of the $C_{2}/C_{1}$ and $C_{3}/C_{1}$ ratio, and the decrease of the $C_{3}/C_{2}$ ratio, between the times $t_{i}$ and $t_{i+1}$ , can be expressed as

$$P_{14}\wedge P_{15}\wedge \neg P_{16}.$$ (25)

Expression Equation 25 can be developed using inference rules as follows:

$$∴\left[\left(P_{8}\Delta P_{9}\right)\vee \neg \left(P_{10}\Delta P_{11}\right)\right]\wedge \left[P_{12}\vee \neg \left(P_{10}\Delta P_{11}\right)\right]\wedge \left[\neg P_{12}\vee \left(P_{8}\Delta P_{9}\right)\right],$$ (26)

by Ponendo Ponens modus contemplating Equations 22–24

$$∴\left\{\neg \left(P_{10}\Delta P_{11}\right)\vee \left[\left(P_{8}\Delta P_{9}\right)\wedge P_{12}\right]\right\}\wedge \left[\neg P_{12}\vee \left(P_{8}\Delta P_{9}\right)\right],$$ (27)

by distributive and association law.

Substituting the logical propositions $P_{8}$ to $P_{12}$ described in Table 2 within Equation 27 yields all combinations of mechanisms of transport and reaction ( RTMs ) considered in this study that result in increases of the $C_{2}/C_{1}$ and $C_{3}/C_{1}$ ratios, while the $C_{3}/C_{2}$ ratio decreases, between two time instants ( $t_{i}$ and $t_{i+1}$ ). Nevertheless, Equation 27 may be simplified significantly by considering the time behavior of intracellular bound concentration ( $C_{3}$ ) and extracellular concentration ( $C_{1}$ ), along with the ratio $C_{2}/C_{1}$ at each time instant. At this point, it is essential to note that EX and IN , DIS and AS , and ECT− and ECT+ are seen as mechanisms that cannot appear together. Consequently, net drug dissociation ( DIS ) and association *(* AS *)* cannot occur simultaneously at any specific location within the tissue domain according to the current analysis. Taking this into account and the fact that in this model, the dissociation of the drug ( DIS ) is the sole mechanism responsible for the decrease in the bound intracellular concentration ( $C_{3}$ ) between two time instants ( $t_{i}$ and $t_{i+1}$ ), when $C_{3}\left(t_{i}\right)>C_{3}\left(t_{i+1}\right)$ , it indicates that dissociation ( DIS ) prevails over association ( AS ) during this interval ( $t_{i}$ and $t_{i+1}$ ), suggesting that $P_{12}=0$ (false), and $P_{8}\Delta P_{9}=P_{9}$ according to the definition of exclusive disjunction and setting $P_{8}=0$ (false) due to the absence of net association. Under these conditions, Equation 27 as can be formulated by Equations 28–30:

$$∴\left\{\neg \left(P_{10}\Delta P_{11}\right)\vee \left[P_{9}\wedge 0\right]\right\}\wedge \left[1\vee P_{9}\right],$$ (28)

$$∴\left\{\neg \left(P_{10}\Delta P_{11}\right)\vee 0\right\}\wedge 1,$$ (29)

by the disjunction and conjunction definition:

$$∴\neg \left(P_{10}\Delta P_{11}\right),$$ (30)

the conjunction and disjunction identification law.

Proposition Equation 30 can be further simplified by considering the $C_{2}/C_{1}$ ratio. As previously demonstrated in Table 1, the direction of net transmembrane transport ( TMT ) at a specific instant is associated with the $C_{2}/C_{1}$ ratio; thus, when $C_{2}/C_{1}<1$ , net internalization takes place ( ∃IN , ∄ EX ), resulting in $P_{10}=0$ (false). According to the definition of exclusive disjunction, Proposition Equation 30 may be expressed as:

$$∴\neg P_{11}.$$ (31)

Substituting the logical statement $P_{11}$ , as shown in Table 2, within the conditional Proposition Equation 31, yields the Equation 32:

∴IN≥ECT+. (32)

**FIGURE 4:**
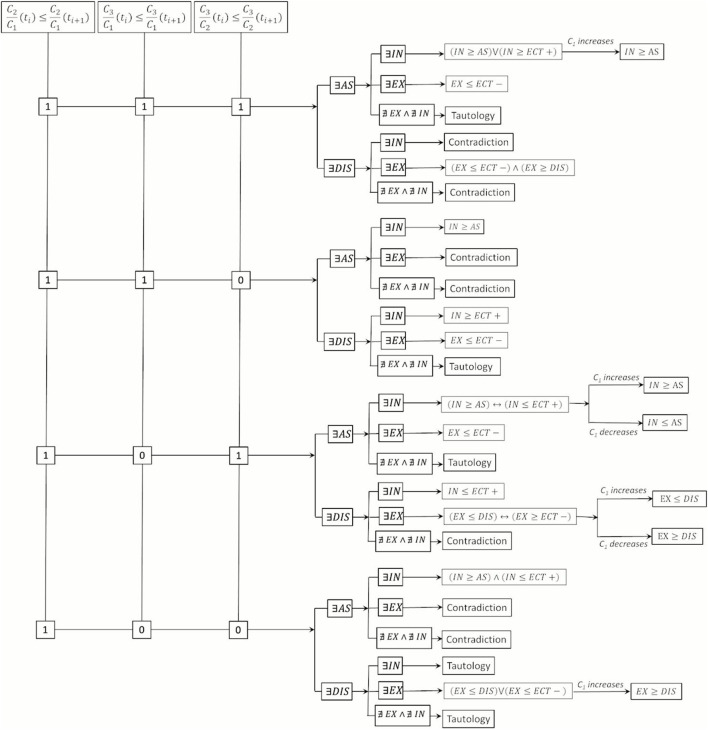
Boolean models for mechanisms of transport and reaction (I).

**FIGURE 5:**
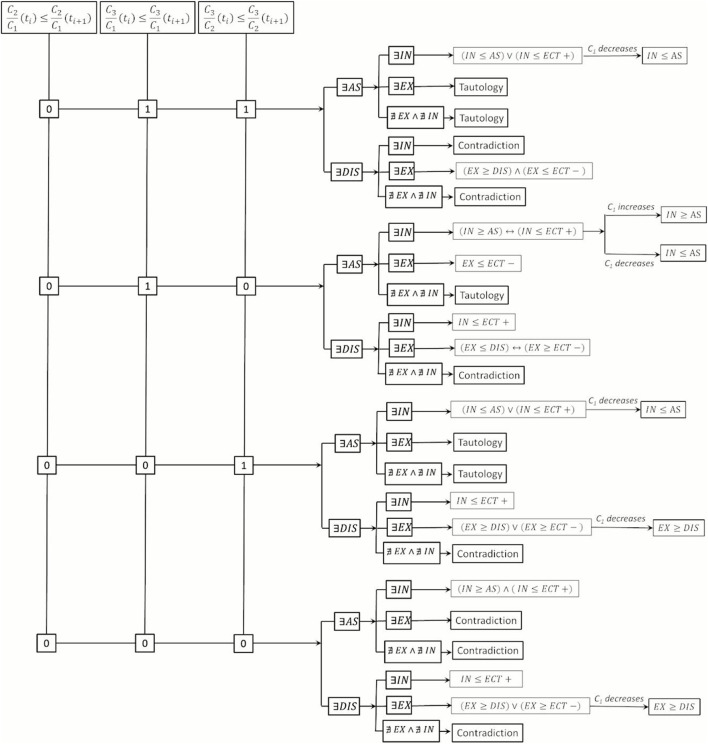
Boolean models for mechanisms of transport and reaction (II).

Bearing in mind a condition that is not in equilibrium $\left(IN\neq ECT+\right)$ , it follows that when dissociation and net internalization occur between two time instants ( $t_{i}$ and $t_{i+1}$ ), and the $C_{2}/C_{1}$ and $C_{3}/C_{1}$ ratios increase, along with a decrease in $C_{3}/C_{2}$ , this suggests that the rate of internalization ( IN ) dominates the positive rate of extracellular transport ( ECT+ ). By applying this procedure, the logical propositions for the remaining situations can be attained. The declarations for all conceivable permutations of the concentration ratios $C_{2}/C_{1}$ , $C_{3}/C_{1}$ , and $C_{3}/C_{2}$ are assumed and presented in Table 3, where *“* D *”* represents the decreases over time, and *“* I *”* represents the increase over time. Figures 4, 5 present the Boolean framework that condenses the outcomes derived from the Boolean evaluations. In Table 3 and Figures 4, 5, the term *“Tautology”* indicates that the scenarios arising from the temporal alterations of $C_{3}/C_{2}$ , $C_{3}/C_{1}$ , and $C_{2}/C_{1}$ , along with the dynamics of drug dissociation/association and externalization/internalization, preclude any definitive conclusions regarding the interactions among the mechanisms of transport and reaction examined in this study. For example, an increase in $C_{2}/C_{1}$ that corresponds to a decrease in $C_{3}/C_{1}$ and $C_{3}/C_{2}$ , alongside the occurrence of drug dissociation and internalization, means that existing reaction and transport mechanisms can interact anyway. Conversely, *“Contradiction”* indicates that the associated scenario lacks physical consistency. For instance, when the temporal behavior of the $C_{2}/C_{1}$ , $C_{3}/C_{1}$ , and $C_{3}/C_{2}$ ratios remain consistent with the previous example, the occurrence of a drug association renders cellular externalization physically impossible.

**TABLE 3:** Comparable logical statements for $C_{3}/C_{2}$ , $C_{3}/C_{1}$ , and $C_{2}/C_{1}$ conceivable scenarios.

Hypothetical situation for ratio time change			Logical declaration of equivalent form	
C_2_/C_1_	C_3_/C_1_	C_3_/C_2_		
I	I	I	*Analysis of general statements and inferences* $P_{14}\wedge P_{15}\wedge P_{16}$ $∴\left[\left(P_{8}\Delta P_{9}\right)\vee \neg \left(P_{10}\Delta P_{11}\right)\right]\wedge \left[P_{12}\vee \neg \left(P_{10}\Delta P_{11}\right)\right]\wedge \left[P_{12}\vee \neg \left(P_{8}\Delta P_{9}\right)\right]$ , by Ponendo Ponens modus $∴\left[\left(P_{8}\Delta P_{9}\right)\vee \neg \left(P_{10}\Delta P_{11}\right)\right]\wedge \left\{P_{12}\vee \left[\neg \left(P_{10}\Delta P_{11}\right)\wedge \neg \left(P_{8}\Delta P_{9}\right)\right]\right\}$ , by the law of distributivity	
			*Drug association arises* $\left(\exists AS\right)$ $P_{12}=1$ (true) $P_{8}\Delta P_{9}=P_{8},$ by exclusive disjunction $∴\left[P_{8}\vee \neg \left(P_{10}\Delta P_{11}\right)\right]\wedge \left\{1\vee \left[\neg \left(P_{10}\Delta P_{11}\right)\wedge \neg P_{8}\right]\right\}$ $∴\left[P_{8}\vee \neg \left(P_{10}\Delta P_{11}\right)\right]\wedge 1$ , by the disjunction definition $∴P_{8}\vee \neg \left(P_{10}\Delta P_{11}\right)$ , by the identity law of conjunction	*Internalization takes place* $\left(\exists IN\right)$ *:* $\left(IN\geq AS\right)\vee \left(IN\geq ECT+\right)$ *An increase in C*_*1*_ *leads to* $\left(IN\geq AS\right)$ *Externalization takes place* $\left(\exists EX\right)$ *:* EX≤ECT− *Partial balance condition (* ∄ EX∧∄ IN): Tautology
			*Drug dissociation arises* $\left(\exists DIS\right)$ $P_{12}=0$ (False) $P_{8}\Delta P_{9}=P_{9}$ , by exclusive disjunction $∴\left[P_{9}\vee \neg \left(P_{10}\Delta P_{11}\right)\right]\wedge \left\{0\vee \left[\neg \left(P_{10}\Delta P_{11}\right)\wedge \neg P_{9}\right]\right\}$ $∴\left[P_{9}\vee \neg \left(P_{10}\Delta P_{11}\right)\right]\wedge \left[\neg \left(P_{10}\Delta P_{11}\right)\wedge \neg P_{9}\right]$ , by the identity law of disjunction $∴\left\{\left[P_{9}\vee \neg \left(P_{10}\Delta P_{11}\right)\right]\wedge \neg \left(P_{10}\Delta P_{11}\right)\right\}\wedge \neg P_{9}$ , by association law $∴\left\{\neg \left(P_{10}\Delta P_{11}\right)\vee \left[P_{9}\wedge 0\right]\right\}\wedge \neg P_{9}$ , by the distributive law $∴\left\{\neg \left(P_{10}\Delta P_{11}\right)\vee 0\right\}\wedge \neg P_{9}$ , by the conjunction definition $∴\neg \left(P_{10}\Delta P_{11}\right)\wedge \neg P_{9}$ , by the disjunction definition	*Internalization takes place* $\left(\exists IN\right)$ *Contradiction* *Externalization takes place* $\left(\exists EX\right)$ *:* $\left(EX\leq ECT-\right)\wedge \left(EX\geq DIS\right)$ *Partial balance condition (* ∄ EX∧∄ IN): *Contradiction*
I	I	D	*Analysis of general statements and inferences* $P_{14}\wedge P_{15}\wedge \neg P_{16}$ $∴\left[\left(P_{8}\Delta P_{9}\right)\vee \neg \left(P_{10}\Delta P_{11}\right)\right]\wedge \left[P_{12}\vee \neg \left(P_{10}\Delta P_{11}\right)\right]\wedge \left[\neg P_{12}\vee \left(P_{8}\Delta P_{9}\right)\right]$ , by the Ponendo Ponens modus $∴\left\{\neg \left(P_{10}\Delta P_{11}\right)\vee \left[\left(P_{8}\Delta P_{9}\right)\wedge P_{12}\right]\right\}\wedge \left[\neg P_{12}\vee \left(P_{8}\Delta P_{9}\right)\right]$ , by the distributive and association law	
			*Drug association arises* $\left(\exists AS\right)$ *:* $P_{12}=1$ (true) $P_{8}\Delta P_{9}=P_{8}$ , by exclusive disjunction $∴\left\{\neg \left(P_{10}\Delta P_{11}\right)\vee \left[P_{8}\wedge 1\right]\right\}\wedge \left[0\vee P_{8}\right]$ $∴\left\{\neg \left(P_{10}\Delta P_{11}\right)\vee P_{8}\right\}\wedge P_{8}$ , using the identity law of conjunction and disjunction $∴P_{8}\vee \left[\neg \left(P_{10}\Delta P_{11}\right)\wedge 0\right]$ , by distributivity law $∴P_{8}$ , by the disjunction and conjunction definition	*Internalization takes place* $\left(\exists IN\right)$ *:* IN≥AS *Externalization occurs* $\left(\exists EX\right)$ *Contradiction* *Partial balance condition (* ∄ EX∧∄ IN): *Contradiction*
			*Drug dissociation arises* $\left(\exists DIS\right)$ $P_{12}=0$ (false) $P_{8}\Delta P_{9}=P_{9}$ , by exclusive disjunction $∴\left\{\neg \left(P_{10}\Delta P_{11}\right)\vee \left[P_{9}\wedge 0\right]\right\}\wedge \left[1\vee P_{9}\right]$ $∴\left\{\neg \left(P_{10}\Delta P_{11}\right)\vee 0\right\}\wedge 1$ *,* by the conjunction and disjunction definition $∴\neg \left(P_{10}\Delta P_{11}\right)$ *,* identity law of conjunction and disjunction	*Internalization takes place* $\left(\exists IN\right)$ IN≥ECT+ *Externalization takes place* $\left(\exists EX\right)$ EX≤ECT− *Partial balance condition (* ∄ EX∧∄ IN): *Tautology*
I	D	I	*Analysis of general statements and inferences* $P_{14}\wedge \neg P_{15}\wedge P_{16}$ $∴\left[\left(P_{8}\Delta P_{9}\right)\vee \neg \left(P_{10}\Delta P_{11}\right)\right]\wedge \left\{\left[\neg P_{12}\vee \left(P_{10}\Delta P_{11}\right)\right]\wedge \left[P_{12}\vee \neg \left(P_{8}\Delta P_{9}\right)\right]\right\}$ , by Ponendo Ponens modus and association law $∴\left[\left(P_{8}\Delta P_{9}\right)\vee \neg \left(P_{10}\Delta P_{11}\right)\right]\wedge \left[\left(P_{10}\Delta P_{11}\right)\vee \neg \left(P_{8}\Delta P_{9}\right)\right]$ , using the resolution law $∴\left[\left(P_{10}\Delta P_{11}\right)\to \left(P_{8}\Delta P_{9}\right)\right]\wedge \left[\left(P_{8}\Delta P_{9}\right)\to \left(P_{10}\Delta P_{11}\right)\right]$ , using disjunctive conditional $∴\left(P_{8}\Delta P_{9}\right)↔\left(P_{10}\Delta P_{11}\right)$ , using the biconditional definition	
			*Drug association arises* $\left(\exists AS\right)$ $P_{8}\Delta P_{9}=P_{8}$ , by exclusive disjunction $∴P_{8}↔\left(P_{10}\Delta P_{11}\right)$	*Internalization takes place* $\left(\exists IN\right)$ $\left(IN\geq AS\right)↔\left(IN\leq ECT+\right)$ *An increase in C*_*1*_ *results in* $\left(IN\geq AS\right)$ *A decrease in C*_*1*_ *results in* $\left(IN\leq AS\right)$ *Externalization takes place* $\left(\exists EX\right)$ *:* EX≤ECT− *Partial equilibrium condition (* ∄ EX∧∄ IN): *Tautology*
			*Drug dissociation arises* $\left(\exists DIS\right)$ $P_{8}\Delta P_{9}=P_{9}$ , by exclusive disjunction $∴P_{9}↔\left(P_{10}\Delta P_{11}\right)$	*Internalization takes place* $\left(\exists IN\right)$ *:* IN≤ECT+ *Externalization takes place* $\left(\exists EX\right)$ $\left(EX\leq DIS\right)↔\left(EX\geq ECT-\right)$ *An increase in C*_*1*_ *leads to* $\left(EX\leq DIS\right)$ *An increase in C*_*1*_ *leads to* $\left(EX\geq DIS\right)$ *Partial balance condition (* ∄ EX∧∄ IN): *Contradiction*
I	D	D	*Analysis of general statements and inferences* $P_{14}\wedge \neg P_{15}\wedge \neg P_{16}$ $∴\left[\left(P_{8}\Delta P_{9}\right)\vee \neg \left(P_{10}\Delta P_{11}\right)\right]\wedge \left\{\left[\neg P_{12}\vee \left(P_{10}\Delta P_{11}\right)\right]\wedge \left[{\neg P}_{12}\vee \left(P_{8}\Delta P_{9}\right)\right]\right\}$ , by Ponendo Ponens modus and association law $∴\left[\left(P_{8}\Delta P_{9}\right)\vee \neg \left(P_{10}\Delta P_{11}\right)\right]\wedge \left\{{\neg P}_{12}\vee \left[\left(P_{8}\Delta P_{9}\right)\wedge \left(P_{10}\Delta P_{11}\right)\right]\right\}$ , by distributivity law	
			*Drug association arises* $\left(\exists AS\right)$ $P_{12}=1$ (True) $P_{8}\Delta P_{9}=P_{8}$ , by exclusive disjunction $∴\left[P_{8}\vee \neg \left(P_{10}\Delta P_{11}\right)\right]\wedge \left\{0\vee \left[P_{8}\wedge \left(P_{10}\Delta P_{11}\right)\right]\right\}$ $\left[P_{8}\vee \neg \left(P_{10}\Delta P_{11}\right)\right]\wedge \left[P_{8}\wedge \left(P_{10}\Delta P_{11}\right)\right]$ , by disjunctive identity $\left\{\left[P_{8}\vee \neg \left(P_{10}\Delta P_{11}\right)\right]\wedge P_{8}\right\}\wedge \left(P_{10}\Delta P_{11}\right)$ , by association law $\left\{P_{8}\vee \left[\neg \left(P_{10}\Delta P_{11}\right)\wedge 0\right]\right\}\wedge \left(P_{10}\Delta P_{11}\right)$ , by distributivity law $P_{8}\wedge \left(P_{10}\Delta P_{11}\right)$ , by the disjunction and conjunction definition	*Internalization takes place* $\left(\exists IN\right)$ $\left(IN\geq AS\right)\wedge \left(IN\leq ECT+\right)$ *Externalization takes place* $\left(\exists EX\right)$ *Contradiction* *Partial balance condition (* ∄ EX∧∄ IN): *Contradiction*
			*Drug dissociation arises* $\left(\exists DIS\right)$ $P_{12}=0$ (False) $P_{8}\Delta P_{9}=P_{9},$ by exclusive disjunction $∴\left[P_{9}\vee \neg \left(P_{10}\Delta P_{11}\right)\right]\wedge \left\{1\vee \left[P_{9}\wedge \left(P_{10}\Delta P_{11}\right)\right]\right\}$ $∴P_{9}\vee \neg \left(P_{10}\Delta P_{11}\right)$ , by the disjunction and conjunction definition	*Internalization takes place* $\left(\exists IN\right)$ *: Tautology* *Externalization takes place* $\left(\exists EX\right)$ $\left(EX\leq DIS\right)\vee \left(EX\leq ECT-\right)$ *An increase in C*_*1*_ *leads to* $\left(EX\leq DIS\right)$ *Partial balance condition (* ∄ EX∧∄ IN): *Tautology*
D	I	I	*Analysis of general statements and inferences* $\neg P_{14}\wedge P_{15}\wedge P_{16}$ $∴\left[\neg \left(P_{8}\Delta P_{9}\right)\vee \left(P_{10}\Delta P_{11}\right)\right]\wedge \left\{\left[P_{12}\vee \neg \left(P_{10}\Delta P_{11}\right)\right]\wedge \left[P_{12}\vee \neg \left(P_{8}\Delta P_{9}\right)\right]\right\}$ , by Ponendo Ponens modus and association law $∴\left[\neg \left(P_{8}\Delta P_{9}\right)\vee \left(P_{10}\Delta P_{11}\right)\right]\wedge \left\{P_{12}\vee \left[\neg \left(P_{10}\Delta P_{11}\right)\wedge \neg \left(P_{8}\Delta P_{9}\right)\right]\right\}$ , by distributivity law	
			*Drug association arises* $\left(\exists AS\right)$ $P_{12}=1$ (true) $P_{8}\Delta P_{9}=P_{8}$ , using exclusive disjunction $∴\left[{\neg P}_{8}\vee \left(P_{10}\Delta P_{11}\right)\right]\wedge \left\{1\vee \left[\neg \left(P_{10}\Delta P_{11}\right)\wedge \neg P_{8}\right]\right\}$ $∴\left[\neg P_{8}\vee \left(P_{10}\Delta P_{11}\right)\right]\wedge 1$ , using the disjunction definition $∴{\neg P}_{8}\vee \left(P_{10}\Delta P_{11}\right)$ , using the identity law of conjunction	*Internalization takes place* $\left(\exists IN\right)$ $\left(IN\leq AS\right)\vee \left(IN\leq ECT+\right)$ *A decrease in C*_*1*_ *leads to* $\left(IN\leq AS\right)$ *Externalization takes place* $\left(\exists EX\right)$ *: Tautology* *Partial balance condition (* ∄ EX∧∄ IN): *Tautology*
			*Drug dissociation arises* $\left(\exists DIS\right)$ $P_{12}=0$ (False) $P_{8}\Delta P_{9}=P_{9}$ , by exclusive disjunction $∴\left[{\neg P}_{9}\vee \left(P_{10}\Delta P_{11}\right)\right]\wedge \left\{0\vee \left[\neg \left(P_{10}\Delta P_{11}\right)\wedge \neg P_{9}\right]\right\}$ $∴\left[\neg P_{9}\vee \left(P_{10}\Delta P_{11}\right)\right]\wedge \left[\neg \left(P_{10}\Delta P_{11}\right)\wedge \neg P_{9}\right]$ , by the identity law of disjunction $∴\left\{\left[\neg P_{9}\vee \left(P_{10}\Delta P_{11}\right)\right]\wedge \neg P_{9}\right\}\wedge \neg \left(P_{10}\Delta P_{11}\right)$ , by association law $∴\left\{\neg P_{9}\vee \left[\left(P_{10}\Delta P_{11}\right)\wedge 0\right]\right\}\wedge \neg \left(P_{10}\Delta P_{11}\right)$ , by distributive law $∴\left\{\neg P_{9}\vee 0\right\}\wedge \neg \left(P_{10}\Delta P_{11}\right)$ , by the conjunction definition $∴\neg P_{9}\wedge \neg \left(P_{10}\Delta P_{11}\right)$ , by the disjunction definition	*Internalization takes place* $\left(\exists IN\right)$ *Contradiction* *Externalization takes place* $\left(\exists EX\right)$ $\left(EX\geq DIS\right)\wedge \left(EX\leq ECT-\right)$ *Partial balance condition (* ∄ EX∧∄ IN): *Contradiction*
D	I	D	*Analysis of general statements and inferences* $\neg P_{14}\wedge P_{15}\wedge \neg P_{16}$ $∴\left[\neg \left(P_{8}\Delta P_{9}\right)\vee \left(P_{10}\Delta P_{11}\right)\right]\wedge \left\{\left[P_{12}\vee \neg \left(P_{10}\Delta P_{11}\right)\right]\wedge \left[\neg P_{12}\vee \left(P_{8}\Delta P_{9}\right)\right]\right\}$ , by Ponendo Ponens modus and association law $∴\left[\neg \left(P_{8}\Delta P_{9}\right)\vee \left(P_{10}\Delta P_{11}\right)\right]\wedge \left[\left(P_{8}\Delta P_{9}\right)\vee \neg \left(P_{10}\Delta P_{11}\right)\right]$ , using resolution $∴\left[\left(P_{8}\Delta P_{9}\right)\to \left(P_{10}\Delta P_{11}\right)\right]\wedge \left[\left(P_{10}\Delta P_{11}\right)\to \left(P_{8}\Delta P_{9}\right)\right]$ , using the disjunctive conditional $∴\left(P_{8}\Delta P_{9}\right)↔\left(P_{10}\Delta P_{11}\right)$ , using the biconditional definition	
			*Drug association arises* $\left(\exists AS\right)$ $P_{8}\Delta P_{9}=P_{8},$ by exclusive disjunction $∴P_{8}↔\left(P_{10}\Delta P_{11}\right)$	*Internalization takes place* $\left(\exists IN\right)$ $\left(IN\geq AS\right)↔\left(IN\leq ECT+\right)$ *An increase in C*_*1*_ *leads to* $\left(IN\geq AS\right)$ *A decrease in C*_*1*_ *results in* $\left(IN\leq AS\right)$ *Externalization takes place* $\left(\exists EX\right)$ *:* EX≤ECT− *Partial balance condition (* ∄ EX∧∄ IN): *Tautology*
			*Drug dissociation arises* $\left(\exists DIS\right)$ $P_{8}\Delta P_{9}=P_{9}$ , by exclusive disjunction $∴P_{9}↔\left(P_{10}\Delta P_{11}\right)$	*Internalization takes place* $\left(\exists IN\right)$ *:* IN≤ECT+ *Externalization takes place* $\left(\exists EX\right)$ $\left(EX\leq DIS\right)↔\left(EX\geq ECT-\right)$ *An increase in C*_*1*_ *leads to* $\left(EX\leq DIS\right)$ *A decrease in C*_*1*_ *leads to* $\left(EX\geq DIS\right)$ *Partial balance condition (* ∄ EX∧∄ IN): *Contradiction*
D	D	I	*Analysis of general statements and inferences* $\neg P_{14}\wedge \neg P_{15}\wedge P_{16}$ $∴\left[\neg \left(P_{8}\Delta P_{9}\right)\vee \left(P_{10}\Delta P_{11}\right)\right]\wedge \left\{\left[\neg P_{12}\vee \left(P_{10}\Delta P_{11}\right)\right]\wedge \left[P_{12}\vee \neg \left(P_{8}\Delta P_{9}\right)\right]\right\}$ , by Ponendo Ponens modus and association law $∴\left[\neg \left(P_{8}\Delta P_{9}\right)\vee \left(P_{10}\Delta P_{11}\right)\right]\wedge \left[\left(P_{10}\Delta P_{11}\right)\vee \neg \left(P_{8}\Delta P_{9}\right)\right]$ , using the resolution law $∴\neg \left(P_{8}\Delta P_{9}\right)\vee \left(P_{10}\Delta P_{11}\right)$ , using the idempotent law	
			*Drug association arises* $\left(\exists AS\right)$ $P_{8}\Delta P_{9}=P_{8},$ by exclusive disjunction $∴\neg P_{8}\vee \left(P_{10}\Delta P_{11}\right)$	*Internalization takes place* $\left(\exists IN\right)$ $\left(IN\leq AS\right)\vee \left(IN\leq ECT+\right)$ *A decrease in C*_*1*_ *leads to* $\left(IN\leq AS\right)$ *Externalization takes place* $\left(\exists EX\right)$ *: Tautology* *Partial balance condition (* ∄ EX∧∄ IN): *Tautology*
			*Drug dissociation arises* $\left(\exists DIS\right):$ $P_{8}\Delta P_{9}=P_{9}$ , by exclusive disjunction $∴\neg P_{9}\vee \left(P_{10}\Delta P_{11}\right)$	*Internalization takes place* $\left(\exists IN\right)$ *:* IN≤ECT+ *Externalization takes place* $\left(\exists EX\right)$ $\left(EX\geq DIS\right)\vee \left(EX\geq ECT-\right)$ *A decrease in C*_*1*_ *leads to* $\left(EX\geq DIS\right)$ *Partial balance condition (* ∄ EX∧∄ IN): *Contradiction*
D	D	D	*Analysis of general statements and inferences* $\neg P_{14}\wedge \neg P_{15}\wedge \neg P_{16}$ $∴\left[\neg \left(P_{8}\Delta P_{9}\right)\vee \left(P_{10}\Delta P_{11}\right)\right]\wedge \left\{\left[\neg P_{12}\vee \left(P_{10}\Delta P_{11}\right)\right]\wedge \left[{\neg P}_{12}\vee \left(P_{8}\Delta P_{9}\right)\right]\right\}$ , by Ponendo Ponens modus and association law $∴\left[\neg \left(P_{8}\Delta P_{9}\right)\vee \left(P_{10}\Delta P_{11}\right)\right]\wedge \left\{{\neg P}_{12}\vee \left[\left(P_{8}\Delta P_{9}\right)\wedge \left(P_{10}\Delta P_{11}\right)\right]\right\}$ , by distributivity law	
			*Drug association arises* $\left(\exists AS\right)$ $P_{12}=1$ (true) $P_{8}\Delta P_{9}=P_{8}$ , by exclusive disjunction $∴\left[\neg P_{8}\vee \left(P_{10}\Delta P_{11}\right)\right]\wedge \left\{0\vee \left[P_{8}\wedge \left(P_{10}\Delta P_{11}\right)\right]\right\}$ $\left[\neg P_{8}\vee \left(P_{10}\Delta P_{11}\right)\right]\wedge \left[P_{8}\wedge \left(P_{10}\Delta P_{11}\right)\right]$ , using disjunctive identity $\left\{\left[\neg P_{8}\vee \left(P_{10}\Delta P_{11}\right)\right]\wedge \left(P_{10}\Delta P_{11}\right)\right\}\wedge P_{8}$ , using the association law $\left\{\left(P_{10}\Delta P_{11}\right)\vee \left[P_{8}\wedge 0\right]\right\}\wedge P_{8}$ , using distributivity law $P_{8}\wedge \left(P_{10}\Delta P_{11}\right)$ , using the disjunction and conjunction definition	*Internalization takes place* $\left(\exists IN\right)$ $\left(IN\geq AS\right)\wedge \left(\;IN\leq ECT+\right)$ *Externalization takes place* $\left(\exists EX\right)$ *Contradiction* *Partial balance condition (* ∄ EX∧∄ IN): *Contradiction*
			*Drug dissociation arises* $\left(∄AS\right)$ $P_{12}=0$ (false) $P_{8}\Delta P_{9}=P_{9}$ , using exclusive diffusion $∴\left[\neg P_{9}\vee \left(P_{10}\Delta P_{11}\right)\right]\wedge \left\{1\vee \left[P_{9}\wedge \left(P_{10}\Delta P_{11}\right)\right]\right\}$ $∴\neg P_{9}\vee \left(P_{10}\Delta P_{11}\right)$ , using the disjunction and conjunction definition	*Internalization takes place* $\left(\exists IN\right)$ *:* IN≤ECT+ *Externalization takes place* $\left(\exists EX\right)$ $\left(EX\geq DIS\right)\vee \left(EX\geq ECT-\right)$ *A decrease in C*_*1*_ *leads to* $\left(EX\geq DIS\right)$ *Partial balance condition (* ∄ EX∧∄ IN): *Contradiction*

## 4 Simulation planning

Using the previously reported Boolean model, this work investigates how the inlet blood velocity ( $\lambda_{inl}$ ), pharmacokinetic profile type ( PK ), and electric field magnitude ( E ) affect the interaction among the RTMs during the treatment with electrochemotherapy. Three values of $E\;\left(0,46,y\;70\;kV/m\right)$ , two values of PK (one-short tri-exponential, or TPK , and uniform, or UPK ) and three values of $\lambda_{inl}$ $\left(1\times 10^{-2}m/s\right.$ , $1\times 10^{-3}m/s$ , and $\left.1\times 10^{-4}m/s\right)$ are taken into consideration in the computational experiments. The remaining parameters are fixed. Table 4 displays both fixed and variable parameters.

**TABLE 4:** Numerical simulation parameters: variable and fixed.

Electrical characteristic				
	Tissue		Wall of vessel	
	Value	Reference	Value	Reference
$E_{rev}$ (kV/m)	46	Boyd and Becker (2016)	46	Ramirez et al. (2020)
$E_{irrev}$ (kV/m)	70	Boyd and Becker (2016)	175	Ramirez et al. (2020)
α*	10	Boyd and Becker (2016)	10	Boyd and Becker (2016)
β*	8	Boyd and Becker (2016)	8	Boyd and Becker (2016)
$\sigma_{\max}$ (S/m)	3.141 × 10^−1^	Boyd and Becker (2016)	6.250 × 10^−1^	Fiederer et al. (2016)
$\sigma_{\min}$ (S/m)	1.998 × 10^−2^	Boyd and Becker (2016)	0.630 × 10^−2^	Fiederer et al. (2016)
$\tau_{i}$ (s)	100	Boyd and Becker (2016)	100	Fiederer et al. (2016)

Properties of transport and reaction		
	Value	Reference
$C_{o}$ (μM)	2.6 × 10^3^	Hubbard et al. (2017), Groh et al. (2014)
$k_{o}$ (m/s)	2.5 × 10^−6^	Hubbard et al. (2017), Evans et al. (2009)
$k_{1,\min}$ (m/s)	1.0 × 10^−9^	Hubbard et al. (2017), Groh et al. (2014)
$k_{1,\max}$ (m/s)	1.0 × 10^−6^	Hubbard et al. (2017), Groh et al. (2014)
$k_{2}$ (μM^−1^.s^−1^)	0.9 × 10^−6^	Hubbard et al. (2017), Groh et al. (2014)
$k_{-2}$ (s^−1^)	14 × 10^−6^	Hubbard et al. (2017), Groh et al. (2014)
$k_{v,\min}$ (m/s)	2.8 × 10^−6^	Hubbard et al. (2017), Eikenberry (2009)
$k_{v,\max}$ (m/s)	4.4 × 10^−6^	Hubbard et al. (2017)
$k_{vc,\min}$ (m/Pa.s)	2.7 × 10^−12^	Zhan et al. (2014), Goh et al. (2001)
$k_{vc,\max}$ (m/Pa.s)	2.1 × 10^−11^	Zhan et al. (2014), Goh et al. (2001)
Do (m^2^/s)	5.0 × 10^−11^	Hubbard et al. (2017), Evans et al. (2009), Lee et al. (2004)

Properties of geometry		
	Value	Reference
$r_{vo}$ (μm)	16	Hubbard et al. (2017), Groh et al. (2014), Harada et al. (2008)
$R_{cord}$ (μm)	200	Hubbard et al. (2017), Groh et al. (2014), Lee et al. (2004)
$L_{cord}$ (μm)	400	Hubbard et al. (2017), Groh et al. (2014), Thurber and Weissleder (2011)
$\delta_{1,o}$	5.88 × 10^−2^	Hubbard et al. (2017), Groh et al. (2014), Evans et al. (2009), Vestvik et al. (2007)
$\delta_{2,o}$	9.41 × 10^−1^	Hubbard et al. (2017), Groh et al. (2014), Evans et al. (2009), Vestvik et al. (2007)
αo (m^−1^)	1.94028 × 10^5^	Hubbard et al. (2017), Groh et al. (2014), Evans et al. (2009), Vestvik et al. (2007)

Details of the electroporation protocol		
	Value	Reference
E (kV/m)	[0, 46, 70]	Authors
$d_{pulses}$ (s)	10	Authors
$T_{ep}$ (min)	10	Authors
$N_{pulses}$	1–5	Authors
$t_{pulse}$ (μs)	100–1,000	Authors
$f_{pulse}$ (Hz)	1–10	Authors
$N_{ep}$	6	Authors

Details about treatment with chemotherapy		
	Value	Reference
Drug type	Doxorubicin	Authors
$M_{w,DOX}$ (g/mol)	543.52	National Library of Medicine, 2022
Pharmacokinetic profiles	One-short tri-exponential, uniform	Hubbard et al. (2017)
$T_{chemo}$ (hr)	24	Authors

Details about the model of vasoconstriction		
	Value	Reference
$\tau_{r}$ (s)	330	Vélez Salazar and Patiño Arcila (2023a)
$m_{r}{\;\left(V/m\right)}^{-1}$	6.49 × 10^−5^	Vélez Salazar and Patiño Arcila (2023a)
${\hat{r}}_{\min}$	0.25	Vélez Salazar and Patiño Arcila (2023a)

Blood description		
	Value	Reference
Inlet velocity, $\lambda_{inl}$ (m/s)	[1 × 10^−4^, 1 × 10^−3^, 1 × 10^−2^]	Authors
Inlet pressure, $p_{inl}$ (mm Hg)	150	Mohammed et al. (2021)
Permeate pressure, $p_{p}$ (mm Hg)	0	Authors
Viscosity at 37 °C, μ (cps)	1.24	Nader et al. (2019)

The temporal dynamics of drug concentration in the circulatory system ( $C_{v}$ ) are contingent upon the pharmacokinetic profile associated with drug administration (Vélez Salazar and Patiño Arcila, 2025). The equations relevant to the profiles examined in this context are (Sorg et al., 2008; Robert et al., 1982; El-Kareh and Secomb, 2005):

One-short tri-exponential infusion ( TPK ) by Equation 33:

$${\left.C_{v}\left(t\right)\right|}_{z=0}=\left\{\begin{array}{l} \frac{D_{o}}{\tau^{\prime }}\left[\frac{A^{\prime }}{A^{\prime \prime }}\left(1-e^{-A^{\prime \prime }t}\right)+\frac{B^{\prime }}{B^{\prime \prime }}\left(1-e^{-B^{\prime \prime }t}\right)+\frac{C^{\prime }}{C^{\prime \prime }}\left(1-e^{-C^{\prime \prime }t}\right)\right],\\ \;fort\leq \tau^{\prime },\\ \frac{D_{o}}{\tau^{\prime }}\left[\frac{A^{\prime }}{A^{\prime \prime }}\left(e^{A^{\prime \prime }\tau^{\prime }}-1\right)e^{-A^{\prime \prime }t}+\frac{B^{\prime }}{B^{\prime \prime }}\left(e^{B^{\prime \prime }\tau^{\prime }}-1\right)e^{-B^{\prime \prime }t}\right.\\ \;\left.+\frac{C^{\prime }}{C^{\prime \prime }}\left(e^{C^{\prime \prime }\tau^{\prime }}-1\right)e^{-C^{\prime \prime }t}\right],fort>\tau^{\prime }, \end{array}\right.$$ (33)

where $D_{o}=1.1983\times 10^{2}\;\mu mol$ , $\tau^{\prime }=180\;s$ , $A^{\prime }=7.46\times 10^{-2}\;l^{-1}$ , $A^{\prime \prime }=2.69\times 10^{-3}\;s^{-1}$ , $B^{\prime }=2.49\times 10^{-3}\;l^{-1}$ , $B^{\prime \prime }=2.83\times 10^{-4}\;s^{-1}$ , $C^{\prime }=5.52\times 10^{-4}\;l^{-1}$ , and $C^{\prime \prime }=1.18\times 10^{-5}\;s^{-1}$ .

Uniform infusion by Equation 34:

$${\left.C_{v}\left(t\right)\right|}_{z=0}=3.85802\times 10^{-2}\;\mu M.$$ (34)

## 5 Results and discussion

**FIGURE 6:**
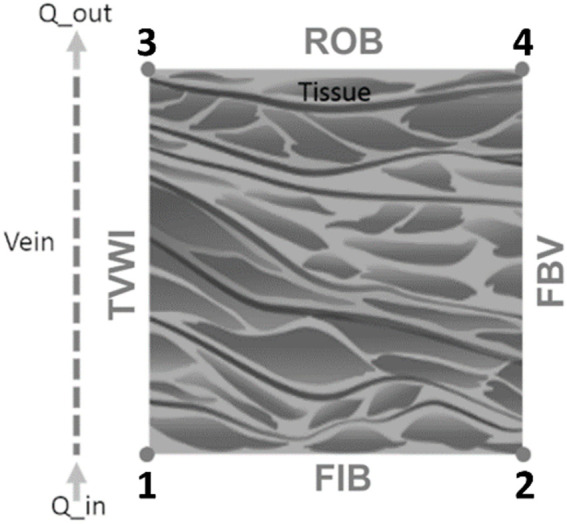
Tumor cord domain boundaries. TVWI : tissue–vessel wall interface, FBV : farthest border vessel, ROB : rear outlet border, and FIB : front inlet border.

In this section, the impact of the combination of inlet blood velocity ( $\lambda_{inl}$ ) and intensity of electric field ( E ) regarding the RTMs contour lines, $C_{2}/C_{1}$ and $C_{3}$ , considering two drug infusion protocols ( TPK and UPK ), is studied (Vélez Salazar and Patiño Arcila, 2023b). To simplify, the tissue boundaries are designed as illustrated in Figure 6: TVWI denotes the interface between vessel wall and tissue where drug extravasation occurs, FBV indicates the vessel’s most distant border with a non-flux condition, ROB represents the rear outlet border, and FIB indicates the front inlet border. Supplementary Material includes specific contour plots pertinent to the findings discussed in the subsequent subsections.

The RTM contour plots account for both non-equilibrium conditions ( RTM different rates) and equilibrium ( RTM equal rates). However, it is important to note that the former condition is more prevalent throughout the treatment, as drug concentrations typically vary between two temporal points, $t_{i}$ and $t_{i+1}$ , based on numerical simulations. Furthermore, the presence of black indicators in any RTM contour plot region indicates that $C_{1}$ , which denotes the extracellular concentrations, diminishes between two temporal points, $t_{i}$ and $t_{i+1}$ .

### 5.1 Analysis of non-electroporated tissue

#### 5.1.1 One-short tri-exponential profile (TPK)

##### 5.1.1.1 Interaction of mechanisms of transport and reaction (RTMs).

**FIGURE 7:**
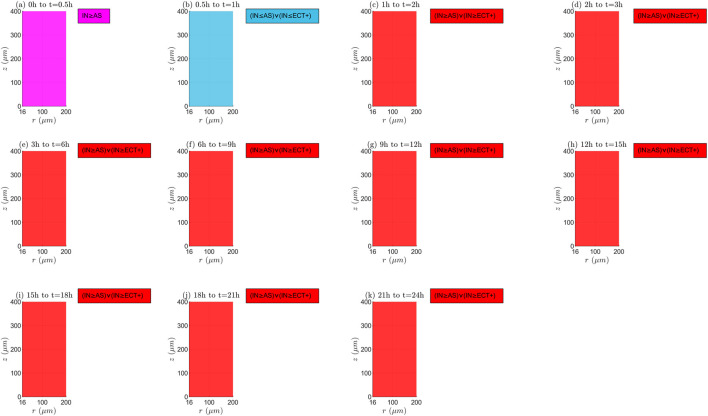
Mechanisms of transport and reaction: E=0kV/m , $\lambda_{inl}=0.0001m/s$ , and TPK . **(a)** 0h to 0.5h, **(b)** 0.5h to 1h, **(c)** 1h to 2h, **(d)** 2h to 3h, **(e)** 3h to 6h, **(f)** 6h to 9h, **(g)** 9h to 12h, **(h)** 12h to 15h, **(i)** 15h to 18h, **(j)** 18h to 21h, **(k)** 21h to 24h.

**FIGURE 8:**
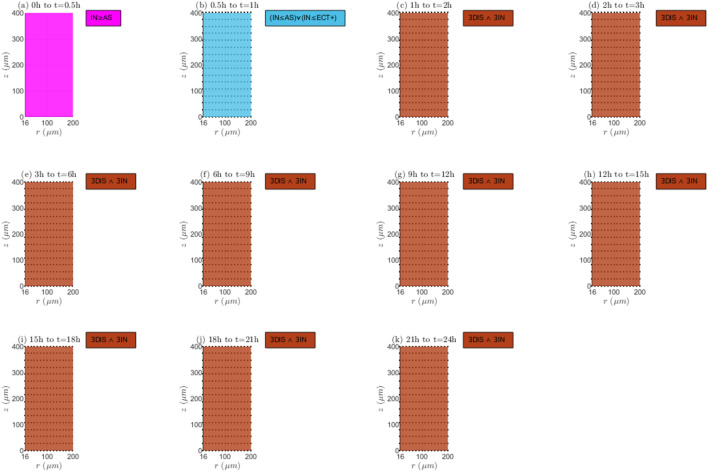
Mechanisms of transport and reaction: E=0kV/m , $\lambda_{inl}=0.001m/s$ , and TPK . **(a)** 0h to 0.5h, **(b)** 0.5h to 1h, **(c)** 1h to 2h, **(d)** 2h to 3h, **(e)** 3h to 6h, **(f)** 6h to 9h, **(g)** 9h to 12h, **(h)** 12h to 15h, **(i)** 15h to 18h, **(j)** 18h to 21h, **(k)** 21h to 24h.

The time and space variations of the interaction among mechanisms of transport and reaction ( RTMs ) for the tissue that has not undergone electroporation and TPK are illustrated in Figures 7, 8 of the manuscript, as well as Figure 1 of the Supplementary Material. It is crucial to highlight that both non-equilibrium (different RTM rates) and equilibrium (equal RTM rates) situations can happen within the domain. For a blood velocity of $\lambda_{inl}=0.0001m/s$ (Figure 7), during the initial interval (0 h–0.5 h), the rate of drug internalization is greater than the rate of association ( IN≥AS ). Through the period 0.5 h–1 h, the IN value may be less than both AS and the positive extracellular transport degree ( ECT+ ). Next, $\left(IN\geq AS\right)\vee \left(IN\geq ECT+\right)$ until the therapy ends. There are no black points at these time instants, which indicates that $C_{1}$ is nonstop increasing. This indicates that IN cannot be higher than ECT+ , which suggests that the internalization degree is greater than association degree ( IN≥AS ), as at the first time instant (Vélez Salazar and Patiño Arcila, 2025; Vélez Salazar and Patiño Arcila, 2023b). For a blood velocity of $\lambda_{inl}=0.001m/s$ (Figure 8), at the initial period (0 h–0.5 h), a greater rate of internalization than association is found ( IN≥AS ), analogous to the preceding case with $\lambda_{inl}=0.0001m/s$ . During the period between 0.5 h and 1 h, the value of IN can be smaller than both AS and ECT+ , and the existence of black points shows a decrease of $C_{1}$ in all tissue zones. Thereby, it can be concluded that the statement IN≤ECT+ is false, leading to the statement IN≤AS being true. Subsequently, until the treatment ends, it can only be deduced that internalization and dissociation occur, and that $C_{1}$ constantly decreases. At the highest blood velocity of $\lambda_{inl}=0.01m/s$ , as shown in Figure 1 Supplementary Material, the RTM behavior is analogous to that examined for $\lambda_{inl}=0.0001m/s$ (Figure 6), except for the black indicators presence from 0.5 h until treatment end, indicating that IN≤AS between 0.5 h and 1 h, and that $\left(IN\geq AS\right)\vee \left(IN\geq ECT+\right)$ from t=1h onward.

##### 5.1.1.2 Rates of internalization and/or externalization behavior in relation to space and time

**FIGURE 9:**
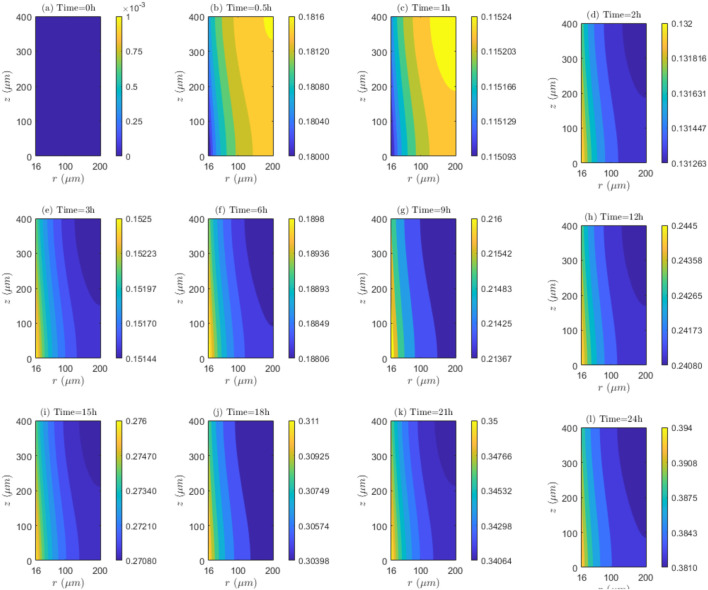
Contour plots of the $C_{2}/C_{1}$ ratio: E=0kV/m , $\lambda_{inl}=0.0001m/s$ , and TPK spatial and temporal dynamics of association and/or dissociation rates. **(a)** Time=0h, **(b)** Time=0.5h, **(c)** Time=1h, **(d)** Time=2h, **(e)** Time=3h, **(f)** Time=6h, **(g)** Time=9h, **(h)** Time=12h, **(i)** Time=15h, **(j)** Time=18h, **(k)** Time=21h, **(l)** Time=24h.

Based on the results of Figure 9, for $\lambda_{inl}=0.001m/s$ , the values of $C_{2}/C_{1}$ are less than 1 at all time instants analyzed, which suggests the continuous presence of the internalization mechanism throughout the therapy. Furthermore, the greatest rates of internalization ( IN ) are observed at the initial treatment hour ( t=1h ), where $C_{2}/C_{1}$ ratios are minimum during the entire period. After 1 hour of treatment, results indicate that the rate of internalization ( IN ) successively decreases throughout the entire duration of the therapy. Regarding the distribution of $C_{2}/C_{1}$ ratio, it is evident that for t=0.5h and t=1h , in TVWI , the IN is superior because $C_{2}/C_{1}$ values are reduced; however, following t=2h , IN is greater in the opposing border, FBV . Additionally, the difference $\prime \prime {\left(C_{2}/C_{1}\right)}_{\max}-$ ${{\left(C_{2}/C_{1}\right)}_{\min}}^{\prime \prime }$ enables quantifying the IN radial uniformity. For the current velocity ( $\lambda_{inl}=0.0001m/s$ ), this difference progressively increases, resulting in a decrease of radial uniformity. For example, at t=0.5h, the change is 0.0016 , at t=12h, it is 0.0037 , and at t=24h , it is 0.013 . For the middle blood velocity of $\lambda_{inl}=0.001\;m/s$ , $theC_{2}/C_{1}$ values in Figure 2 of Supplementary Material are also less than 1 at all time instants, signifying internalization presence, with a rate ( IN ) initially increasing during the first hour followed by a continuous decrease throughout the remaining therapy. For this velocity $\left(\lambda_{inl}=0.001m/s\right)$ , IN in TVWI is higher at t=0.5h and t=1h , and thereafter it is higher in FBV . IN radial distribution becomes more regular at t=1h , as indicated by the difference between ${\left(C_{2}/C_{1}\right)}_{\max}$ and ${\left(C_{2}/C_{1}\right)}_{\min}$ , however, this uniformity gradually decreases over the course of the therapy. Regarding the higher blood velocity $\left(\lambda_{inl}=0.01m/s\right)$ , Figure 3 of Supplementary Material shows that the ratios $C_{2}/C_{1}$ exhibit considerable similarity to the case with $\lambda_{inl}=0.001m/s$ ; this suggests that the increase in blood velocity has a negligible impact on the rate of internalization ( IN ) for these two last cases.

Alternatively, the linearity of the lines of contour of $C_{2}/C_{1}$ explains the drug externalization and/or internalization axial uniformity. For $\lambda_{inl}=0.0001m/s$ , the rate of internalization ( IN ) is not axially uniform at all times, in contrast to the other blood velocities ( $\lambda_{inl}=0.001m/s$ and $\lambda_{inl}=0.01m/s$ ) where this rate is clearly more uniform along the longitudinal direction. This suggests that increasing $\lambda_{inl}$ promotes the axial uniformity of IN .

##### 5.1.1.3 Spatial and temporal dynamics of association and/or dissociation rates

**FIGURE 10:**
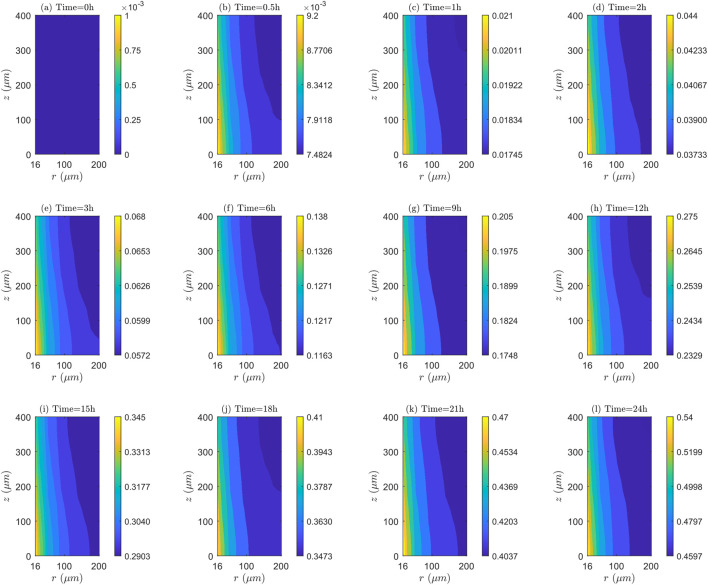
Bound intracellular concentration $C_{3}$ : E=0kV/m , $\lambda_{inl}=0.0001m/s$ , and TPK . **(a)** Time=0h, **(b)** Time=0.5h, **(c)** Time=1h, **(d)** Time=2h, **(e)** Time=3h, **(f)** Time=6h, **(g)** Time=9h, **(h)** Time=12h, **(i)** Time=15h, **(j)** Time=18h, **(k)** Time=21h, **(l)** Time=24h.

Figure 10 demonstrates that for a blood velocity of $\lambda_{inl}=0.0001m/s$ , the bound intracellular concentration ( $C_{3}$ ) continuously increases over time, suggesting the presence of an association throughout the entire therapy. At all time instants, the value of $C_{3}$ is highest in TVWI and lowest in FBV . The difference ${\left(C_{3}\right)}_{\max}-{\left(C_{3}\right)}_{\min}$ enables the quantification of the radial uniformity of drug association and/or dissociation degree ( AS/DIS ). This change grows as time progresses, in a manner that at t=0.5h, this variation is $1.7176\times 10^{-3}\;\mu M$ ; at t=1h , it is 0.00355 μM ; at t=12h, it is 0.0421 μM ; and at t=24h, it is 0.0803 μM . It is also found that the rate of association ( AS ) is greater in the TVWI than the FBV over the entire therapy. For the blood velocity of $\lambda_{inl}=0.001m/s$ , Figure 4 of Supplementary Material allows inferring that $C_{3}$ is highest in TVWI and lowest in FBV , as for the preceding velocity ( $\lambda_{inl}=0.0001m/s$ ). However, in this case, dissociation occurs after t=2h . The variation in the change between ${\left(C_{3}\right)}_{\max}$ and ${\left(C_{3}\right)}_{\min}$ is not monotonous over time. Namely, at t=0.5h , this change is 0.2374 μM ; at t=1h , it is 0.2505 μM ; at t=12h, it is 0.1988 μM ; and at t=24h , it is 0.2101 μM . It also suggests that a prevailing rate of associations/dissociation ( AS/DIS ) at either TVWI and FBV borders does not exist at every time instant. For $\lambda_{inl}=0.01m/s$ , as shown in Figure 5 of Supplementary Material, a similar pattern to the case of $\lambda_{inl}=0.0001m/s$ is observed. Namely, $C_{3}$ has its highest value in TVWI and its lowest value in FBV. In the TVWI , the association degree ( AS ) is larger than FBV during the whole therapy. For all blood velocities, the intracellular bound concentration ( $C_{3}$ ) reaches its peak after 24 h of electrochemotherapy, being larger for the middle blood velocity, $\lambda_{inl}=0.001\;m/s$ .

Conversely, the linearity of the contours of $C_{3}$ is an indication of the axial uniformity of drug association and/or dissociation. The axial distribution of $C_{3}$ is not uniform for the three blood speeds, indicating that the association/dissociation rate is not the same for a set of points equidistant from the TVWI border.

#### 5.1.2 Uniform profile (UPK)

##### 5.1.2.1 Interaction among transport and reaction mechanisms (RTMs)

Figures 6–8 of Supplementary Material depict the mechanisms of transport and reaction ( RTMs ) at all time intervals for the UPK and the three analyzed blood speeds ( $\lambda_{inl}=0.0001m/s$ , $\lambda_{inl}=0.001m/s$ , and $\lambda_{inl}=0.01m/s$ ). When comparing these figures with those from the TPK profile (Figures 6, 7 of the manuscript; Figure 1 of Supplementary Material), the same RTM behavior can be observed, except for the time interval from 0.5 h to 1 h. In this interval, for $\lambda_{inl}=0.0001m/s$ , $\left(IN\geq AS\right)\vee \left(IN\geq ECT+\right)$ and $C_{1}$ increases, resulting in IN≥AS . However, for $\lambda_{inl}=0.001m/s$ and $\lambda_{inl}=0.01m/s$ , $C_{1}$ decreases, and $\left(IN\geq AS\right)\vee \left(IN\geq ECT+\right)$ .

##### 5.1.2.2 Spatial and temporal dynamics of externalization and/or internalization rates

**FIGURE 11:**
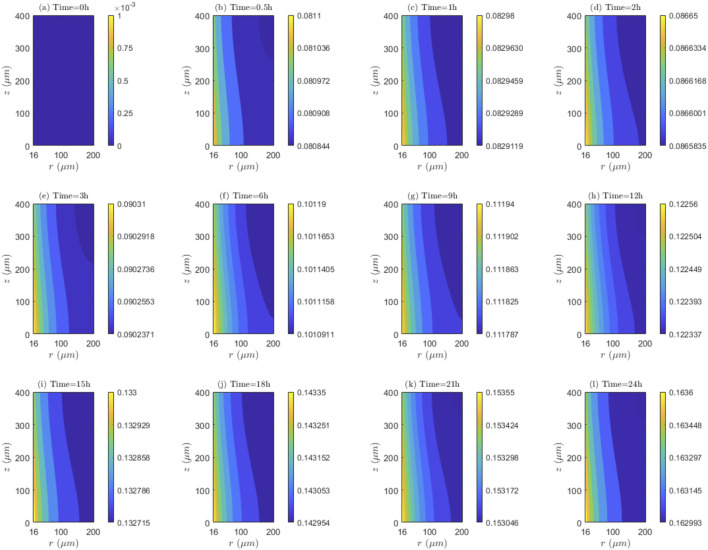
Contour plots of $C_{2}/C_{1}$ values: E=0kV/m , $\lambda_{inl}=0.0001m/s$ , and UPK . **(a)** Time=0h, **(b)** Time=0.5h, **(c)** Time=1h, **(d)** Time=2h, **(e)** Time=3h, **(f)** Time=6h, **(g)** Time=9h, **(h)** Time=12h, **(i)** Time=15h, **(j)** Time=18h, **(k)** Time=21h, **(l)** Time=24h.

For a blood velocity of $\lambda_{inl}=0.0001m/s$ , as shown in Figure 11 of the manuscript, it can be noted that $C_{2}/C_{1}$ values are below 1 at all time instants, demonstrating internalization during the entire treatment, similar to the TPK profile. Moreover, it can be noted that as the values of $C_{2}/C_{1}$ increase, the internalization rates ( IN ) are decreasing over time; additionally, the internalization rate ( IN ) is always greater near the *FBV* boundary. On the other hand, the variation in the difference between ${\left(C_{2}/C_{1}\right)}_{\max}$ and ${\left(C_{2}/C_{1}\right)}_{\min}$ shows no signs of monotony over time, which implies that the uniformity level of IN can either increase or decrease during the therapy. For a blood velocity of $\lambda_{inl}=0.001m/s$ (Figure 9 of Supplementary Material), the same as for TPK , the $C_{2}/C_{1}$ values are smaller than 1 at all time instants, signifying the presence of internalization. As for the last velocity $\left(\lambda_{inl}=0.0001m/s\right)$ , IN decreases and is higher in the FBV throughout the entire period. The difference between ${\left(C_{2}/C_{1}\right)}_{\max}$ and ${\left(C_{2}/C_{1}\right)}_{\min}$ increases after the initial hour, suggesting a decrease in the radial uniformity for the rest of the therapy. For the largest blood velocity, $\lambda_{inl}=0.01m/s$ , Figure 10 of Supplementary Material is nearly identical to such corresponding to $\lambda_{inl}=0.001m/s$ (Figure 9 of Supplementary Material), suggesting that the blood velocity has a minimum impact on the rate of internalization ( IN ) for these cases.

Concerning the IN axial uniformity, the uniformity of the blood velocity of $\lambda_{inl}=0.0001m/s$ is affected over time, whereas for the remaining two blood velocities ( $\lambda_{inl}=0.001m/s$ and $\lambda_{inl}=0.01m/s$ ), it is suitable, coinciding with the previous profile ( TPK ).

##### 5.1.2.3 Spatial and temporal dynamics of association and/or dissociation rates

**FIGURE 12:**
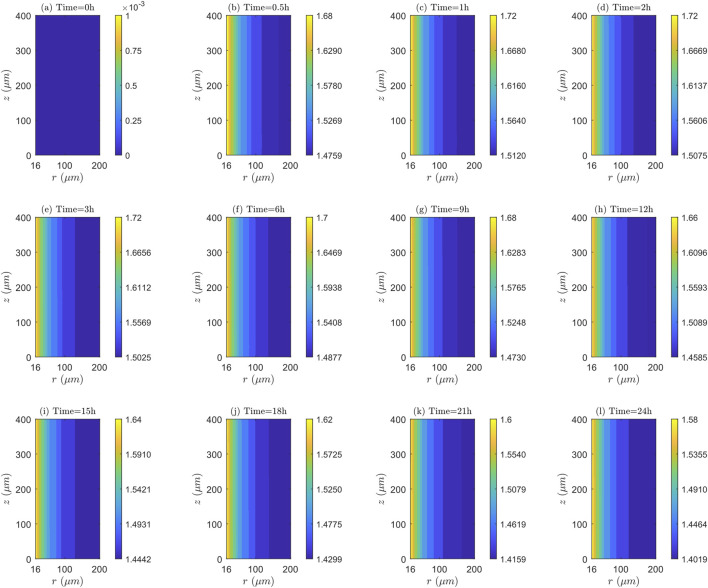
Bound intracellular concentration $C_{3}$ : E=0kV/m , $\lambda_{inl}=0.001m/s$ , and UPK . **(a)** Time=0h, **(b)** Time=0.5h, **(c)** Time=1h, **(d)** Time=2h, **(e)** Time=3h, **(f)** Time=6h, **(g)** Time=9h, **(h)** Time=12h, **(i)** Time=15h, **(j)** Time=18h, **(k)** Time=21h, **(l)** Time=24h.

Figure 11 of Supplementary Material demonstrates that for $\lambda_{inl}=0.0001m/s$ , in an analogous manner to TPK (Figure 10), $C_{3}$ exhibits a uniform increase over the time, indicating the presence of an association; additionally, $C_{3}$ reaches its higher values in the TVWI . The difference between ${\left(C_{3}\right)}_{\max}$ and ${\left(C_{3}\right)}_{\min}$ increases over time. At t=0.5h , it is $1.4502\;x{\;10}^{-3}\;\mu M$ ; at t=1h , it is 0.00308 μM ; at t=12h , it is 0.0354 μM ; and at t=24h, it is 0.0977 μM , indicating that the degree of association ( AS ) is larger in TVWI . Regarding the blood velocity of $\lambda_{inl}=0.001m/s$ , Figure 12 of the manuscript, there is a distinct behavior compared to the preceding example ( $\lambda_{inl}=0.0001m/s$ ). In this case, $C_{3}$ increases only until t=1h and then diminishes, being attributed to the dissociation occurrence throughout a significant part of the treatment. Furthermore, $C_{3}$ reaches higher values in TVWI and smaller values in FBV . The difference between ${\left(C_{3}\right)}_{\max}$ and ${\left(C_{3}\right)}_{\min}$ does not exhibit monotonic behavior in time, suggesting that the AS/DIS may be larger or smaller in TVWI when compared to FBV . For a blood velocity of $\lambda_{inl}=0.01\;m/s$ , Figure 12 of Supplementary Material, as well as in the case of $\lambda_{inl}=0.0001\;m/s$ , shows that an association exists throughout the entire therapy, and $C_{3}$ reaches its higher values in TVWI . In that case, the change between ${\left(C_{3}\right)}_{\max}$ and ${\left(C_{3}\right)}_{\min}$ is progressively increasing, indicating a greater AS in TVWI .

As an important different characteristic of the TPK , the linearity of the contours of $C_{3}$ indicates that the association/dissociation rates ( AS/DIS ) distribute uniformly along the axial direction for the three blood speeds under consideration, suggesting that AS/DIS ratios are nearly identical at specific equidistant points from the TVWI .

#### 5.1.3 Comparison with recent literature of non-electroporated tissues

The geometry, transport, reaction, and pharmacokinetic properties employed in the present research closely resemble those utilized in Groh et al. (2014), where both TPK and UPK were examined for drug delivery in non-electroporated tissues ( E=0 kV/m ). Although the axial variation of drug concentration in the vessel $\left(C_{v}\right)$ was not addressed in the referenced study (Groh et al., 2014), several results align with the current findings. The behavior of the extracellular concentration $\left(C_{1}\right)$ is different between both profiles (see Figure 7 of Groh et al. (2014)). At the first time instants, the initial increase of $C_{1}$ is markedly higher for TPK than for UPK ; then, an important decrease of $C_{1}$ is reported for TPK , while a continuous increase occurs for UPK . This means that larger internalization rates could be expected for UPK than for TPK during most of the treatment because the extracellular concentration $\left(C_{1}\right)$ always increases for the former profile. This agrees with the present results, where smaller values of $C_{2}/C_{1}$ (larger internalization rates) were obtained for UPK (Figure 11 of the manuscript and Figures 9, 10 of Supplementary Material) than TPK (Figure 9 of the manuscript and Figures 2, 3 of Supplementary Material). Internalization is present in both profiles ( UPK and TPK ) at all time instants.

### 5.2 Analysis of tissue that has been electroporated with E=46 kV/m

#### 5.2.1 One short tri-exponential profile (TPK)

##### 5.2.1.1 Interaction of mechanisms of transport and reaction (RTMs)

**FIGURE 13:**
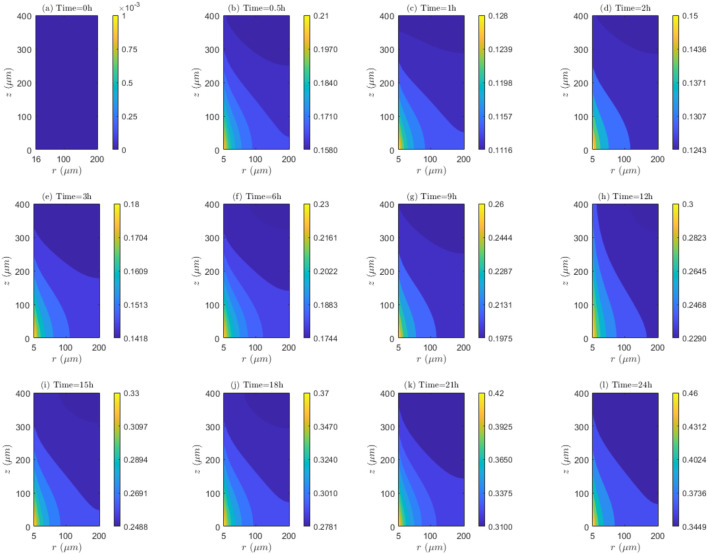
Contour plots of $C_{2}/C_{1}$ values: E=46kV/m , $\lambda_{inl}=0.0001m/s$ , and TPK . **(a)** Time=0h, **(b)** Time=0.5h, **(c)** Time=1h, **(d)** Time=2h, **(e)** Time=3h, **(f)** Time=6h, **(g)** Time=9h, **(h)** Time=12h, **(i)** Time=15h, **(j)** Time=18h, **(k)** Time=21h, **(l)** Time=24h.

Figures 13–15 of the Supplementary Material illustrate the interaction among the RTMs when the tissue undergone electroporation with an electrical field magnitude of E=46kV/m (reversible limit), for three dissimilar blood velocities considered here, namely, $\lambda_{inl}=0.0001m/s$ , $\lambda_{inl}=0.001m/s$ , and $\lambda_{inl}=0.01m/s$ . For the blood velocities of $\lambda_{inl}=0.0001m/s$ and $\lambda_{inl}=0.001m/s$ (Supplementary Material, Figures 13, 14), the RTMs behavior is analogous to the tissue that has not been electroporated ( E=0 kV/m ), with the $C_{1}$ decrease between t=9h and t=12h as the most relevant difference. For the blood velocity of $\lambda_{inl}=0.01m/s$ , Figure 15 of Supplementary Material, the RTMs behavior is identical to the non-electroporated tissue at this velocity.

##### 5.2.1.2 Spatial and temporal dynamics of internalization and/or externalization rates

Figure 13 of the manuscript and Figures 16, 17 of Supplementary Material indicate that some features of the $C_{2}/C_{1}$ ratio behavior change when electrical pulses are administrated to the tissue, while others remain unchanged concerning the non-electroporated tissue. The internalization mechanism can be confirmed during the entire therapy for all blood velocities ( $C_{2}/C_{1}$ is always less than 1). For the specific scenario where $\lambda_{inl}=0.0001km/s$ , Figure 13 of the manuscript, the internalization rate ( IN ) decreases after t=1h ( $C_{2}/C_{1}$ values increase), coinciding with the non-electroporated tissue. In addition, IN is consistently smaller in the TVWI lower section across all time instants. As for the tissue that is not electroporated, the IN radial uniformity diminishes with passage of time according to the progress of the difference between ${\left(C_{2}/C_{1}\right)}_{\max}$ and ${\left(C_{2}/C_{1}\right)}_{\min}$ . Now, for $\lambda_{inl}=0.001m/s$ and $\lambda_{inl}=0.01m/s$ (Supplementary Material, Figures 16, 17), the behavior of IN is identical to that of $\lambda_{inl}=0.0001\;m/s$ (it diminishes as time passes following the initial hour), exhibiting a reduced position throughout the entire TVWI . For these two blood velocities ( $\lambda_{inl}=0.001m/s$ and $\lambda_{inl}=0.01m/s$ ), the reduction of IN after the initial hour also aligns with the findings of the non-electroporated tissue. Moreover, the increase of the difference between ${\left(C_{2}/C_{1}\right)}_{\max}$ and ${\left(C_{2}/C_{1}\right)}_{\min}$ signifies that the IN radial uniformity also diminishes along the time for these velocities ( $\lambda_{inl}=0.001m/s$ and $\lambda_{inl}=0.01m/s$ ). Conversely, the shape of lines of contour of $C_{2}/C_{1}$ points out that for $\lambda_{inl}=0.0001m/s$ and $\lambda_{inl}=0.001m/s$ , the rate of internalization ( IN ) is not axially uniform throughout the time, in contrast with $\lambda_{inl}=0.01m/s$ , where *IN* is homogenous, verifying the favorable influence of increasing $\lambda_{inl}$ on the IN longitudinal uniformity.

##### 5.2.1.3 Spatial and temporal dynamics of association and/or dissociation rates

**FIGURE 14:**
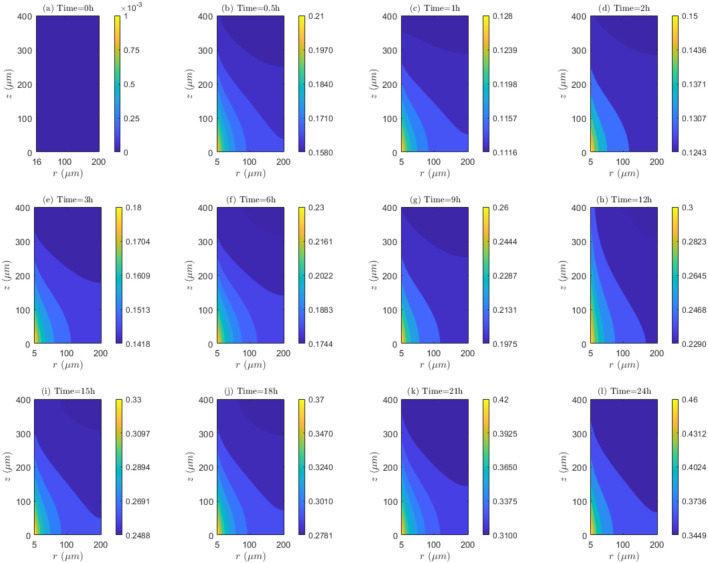
Bound intracellular concentration $C_{3}$ : E=46kV/m , $\lambda_{inl}=0.0001m/s$ , and TPK . **(a)** Time=0h, **(b)** Time=0.5h, **(c)** Time=1h, **(d)** Time=2h, **(e)** Time=3h, **(f)** Time=6h, **(g)** Time=9h, **(h)** Time=12h, **(i)** Time=15h, **(j)** Time=18h, **(k)** Time=21h, **(l)** Time=24h.

As shown in Figure 14 of the manuscript, for $\lambda_{inl}=0.0001m/s$ , $C_{3}$ increases during the treatment, indicating an association. The concentration of $C_{3}$ is highest in TVWI and lowest in FBV . These results look like the findings in the non-electroporate tissue, with the exception that in the present case ( E=46kV/m ), the highest values of $C_{3}$ are situated in the TVWI lower region. The difference between ${\left(C_{3}\right)}_{\max}$ and ${\left(C_{3}\right)}_{\min}$ progressively increases, suggesting that the association rate ( AS ) persists at an increased level in the TVWI , which also coincides with the tissue that is not subjected to electroporation. For a blood velocity of $\lambda_{inl}=0.001m/s$ , Figure 18 of Supplementary Material, the $C_{3}$ changes indicate that association exists at the initial hour, and then dissociation happens, which is also aligned with the behavior observed in the non-electroporated tissue. In contrast, for a blood velocity of $\lambda_{inl}=0.01m/s$ (Figure 19 of Supplementary Material), association takes place across the entire therapy, which also matches with the E=0 kV/m case. For velocities $\lambda_{inl}=0.001m/s$ and $\lambda_{inl}=0.01m/s$ , $C_{3}$ reaches its maximum value in the TVWI lower part. However, the difference between ${\left(C_{3}\right)}_{\max}$ and ${\left(C_{3}\right)}_{\min}$ shows no signs of monotony over time, concluding that there is no predominance of the dissociation rate ( DIS ) for $\lambda_{inl}=0.001m/s$ or association rate ( AS ) for $\lambda_{inl}=0.01m/s$ in any of the radial boundaries ( TVWI or FBV ).

Similar to the scenario where E=0 kV/m , the $C_{3}$ axial distribution for the blood speeds is non-uniform; that is, AS/DIS values differ for equidistant points from TVWI . As in the non-electroporated tissue, the highest $C_{3}$ values are also reached for the middle velocity, $\lambda_{inl}=0.001m/s$ .

#### 5.2.2 Uniform profile (UPK)

##### 5.2.2.1 Interaction of mechanisms of transport and reaction (RTMs)

**FIGURE 15:**
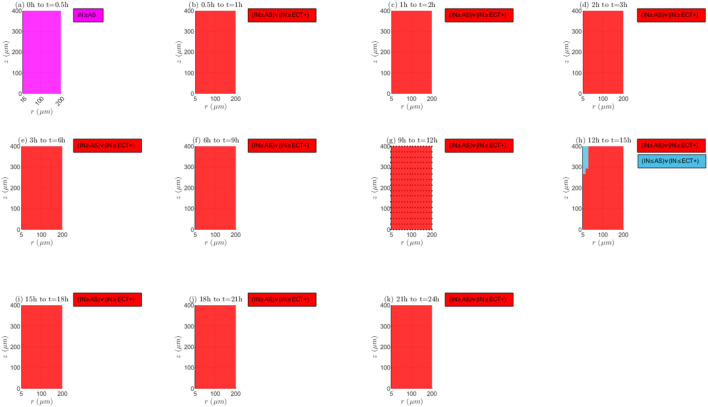
Mechanisms of transport and reaction: E=46kV/m , $\lambda_{inl}=0.0001m/s$ , and UPK . **(a)** 0h to 0.5h, **(b)** 0.5h to 1h, **(c)** 1h to 2h, **(d)** 2h to 3h, **(e)** 3h to 6h, **(f)** 6h to 9h, **(g)** 9h to 12h, **(h)** 12h to 15h, **(i)** 15h to 18h, **(j)** 18h to 21h, **(k)** 21h to 24h.

For the velocity of $\lambda_{inl}=0.0001m/s$ , Figure 15 of the manuscript, at the initial time interval (0 h–0.5 h), it is noted that IN≥AS and $C_{1}$ increases. In the subsequent five time intervals (0.5 h–9 h), it turns out that $\left(IN\geq AS\right)\vee \left(IN\geq ECT+\right)$ , and $C_{1}$ continues to increase, still implying that IN≥AS . From 9 h to 12 h, the behavior is like the previous intervals, but in this case $C_{1}$ diminishes. From t=12h to t=15h , it happens that $\left(IN\leq AS\right)\vee \left(IN\leq ECT+\right)$ at certain locations near the upper part of TVWI , and from t=15h until the treatment ends, it happens that $C_{1}$ increases again and $\left(IN\geq AS\right)\vee \left(IN\geq ECT+\right)$ , entailing that IN≥AS . It is worth noting that this behavior of RTMs shares many characteristics with the one identified for TPK and E=46 kV/m (Figure 13 of Supplementary Material). For the other blood velocities ( $\lambda_{inl}=0.001m/s$ and $\lambda_{inl}=0.01m/s$ *)*, Figures 20, 21 of Supplementary Material show that the RTM behavior is analogous to that observed for TPK with these corresponding velocities, with the difference that in the second time period (0.5 h–1 h), $\left(IN\leq AS\right)\vee \left(IN\leq ECT+\right)$ in the left half domain, and $\left(IN\geq AS\right)\vee \left(IN\geq ECT+\right)$ in the right half domain, respectively, signifying a spatial change in the relationship between IN , AS , and ECT+ within the same time interval.

##### 5.2.2.2 Spatial and temporal dynamics of internalization and/or externalization rates

In Figures 22–24 of Supplementary Material, it is evident that internalization is the dominant process at all time instants for the three blood velocities ( $\lambda_{inl}=0.001m/s$ , $\lambda_{inl}=0.01m/s$ , and $\lambda_{inl}=0.01m/s$ ), while the internalization rates ( IN ) decrease over time (values of $C_{2}/C_{1}$ increase). However, the difference between ${\left(C_{2}/C_{1}\right)}_{\max}$ and ${\left(C_{2}/C_{1}\right)}_{\min}$ shows no signs of monotony over time, suggesting that the radial uniformity of IN can increase or decrease during the therapy. Concerning the rate of internalization ( IN ), when $\lambda_{inl}=0.0001m/s$ , this rate is diminished in the TVWI lower section, except at t=12h when IN is lower along the whole TVWI . When $\lambda_{inl}=0.001m/s$ , IN is lower along the TVWI , except at t=12h , when it is lower only in the upper part of this boundary. When $\lambda_{inl}=0.01m/s$ , IN is also smaller along the TVWI , except at t=12h , when it becomes higher in this boundary.

Concerning the axial distribution of IN , it can be deduced that it is consistent with that of the non-electroporated tissue and the TPK profile for all values of $\lambda_{inl}$ .

##### 5.2.2.3 Spatial and temporal dynamics of association and/or dissociation rates

For a blood velocity of $\lambda_{inl}=0.0001m/s$ , according to Figure 25 of Supplementary Material, $C_{3}$ continuously increases, signifying the presence of association, and the rate of association ( AS ) is increased in the TVWI because the difference between ${\left(C_{3}\right)}_{\max}$ and ${\left(C_{3}\right)}_{\min}$ increases over time. For a blood velocity of $\lambda_{inl}=0.001m/s$ , Figure 26 of Supplementary Material, the variations in $C_{3}$ over time are not monotonous, indicating that both association and dissociation are occurring during the treatment. For a blood velocity of $\lambda_{inl}=0.01m/s$ , Figure 27 of Supplementary Material points out the existence of association during the whole therapy. For the three blood velocities, $C_{3}$ reaches its highest value along the TVWI and its lowest values along the FVB .

The $C_{3}$ axial distribution is uniform for the blood speeds examined, suggesting that the *AS/DIS* are nearly identical at equidistant places from the TVWI . As for the tissue that is not electroporated and TPK profile, the largest values of $C_{3}$ are reached for the middle velocity, $\lambda_{inl}=0.001\;m/s$ .

#### 5.2.3 Comparison with recent literature on tissues electroporated with the reversible electric field

**FIGURE 16:**
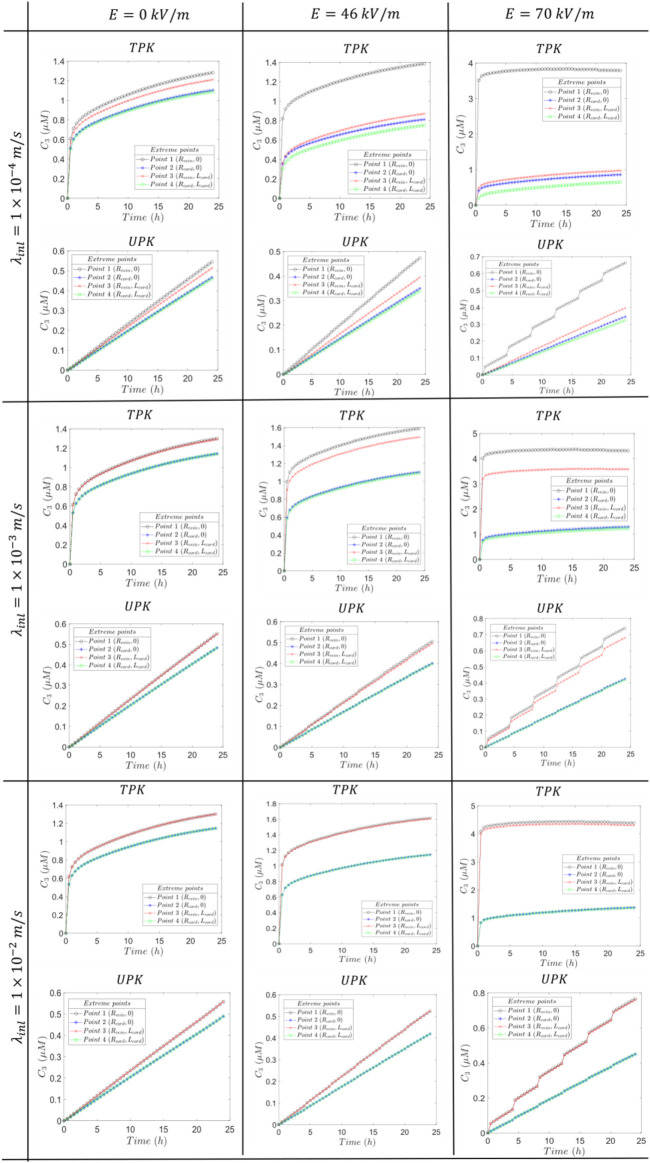
Time behavior of the bound intracellular concentration, $C_{3}$ , for all combinations of E and $\lambda_{inl}$ , and two pharmacokinetic profiles ( TPK and UPK ).

This section demonstrates that when tissue is electroporated with a reversible electric field magnitude $\left(E=46\;kV/m\right)$ , the $C_{2}⁄C_{1}$ ratio, which reflects cellular internalization or externalization rates, decreases radially at most time instants from the TVWI to the FBV for both TPK (Figure 13 of the manuscript; Figures 16, 17 of Supplementary Material) and UPK (Figures 22–24 of Supplementary Material), with a more pronounced effect as $\lambda_{inl}$ increases. This agrees with a recent study (Mondal and Dalal, 2023) that examined the impact of pulse numbers ( $N_{ep}$ ) on the extracellular and intracellular concentrations in a tissue subjected to a reversible electric field of E=28 kV/m , with a uniform extracellular concentration of $C_{1}=1M$ at one boundary of the tumor domain (corresponding to the TVWI boundary in the present work). Notwithstanding the disparity in reversible thresholds ( 28 kV/m in Mondal and Dalal (2023) *versus* 46 kV/m in the present work), it was observed that at a given time instant, the $C_{2}⁄C_{1}$ ratio is larger in TVWI , diminishing toward the outermost boundary ( FBV ), and this pattern persists irrespective of the increase in the number of pulses ( $N_{ep}$ ).

### 5.3 Analysis of tissue that has been electroporated with E = 70kV/m

#### 5.3.1 One-short tri-exponential profile (TPK)

##### 5.3.1.1 Interaction of mechanisms of transport and reaction (RTMs).

For $\lambda_{inl}=0.0001m/s$ and TPK , Figure 40 of Supplementary Material, the RTM behavior until t=9h is highly analogous to the one acquired for E=46 kV/m and E=0 kV/m . After t=9h , some differences can be appreciated. During the 9–12 h period, certain locations near the TVWI lower section exhibit $\left(IN\geq AS\right)\vee \left(IN\geq ECT+\right)\;to\;EX\geq ECT-$ , indicating that in those regions, in a specific moment within this interval, a transition from IN to EX , as well as from positive to negative, extracellular transport takes place. Throughout the specified time frame (9–12 h), $\left(IN\geq AS\right)\vee \left(IN\geq ECT+\right)$ and, in contrast to the preceding periods, $C_{1}$ diminishes. Between 12 h and 15 h, $\left(IN\leq AS\right)\vee \left(IN\leq ECT+\right)$ , and $C_{1}$ increases throughout most of the domain; however, at a specific moment within this period, in certain points of the TVWI lower section, it is noted that $\left(IN\geq AS\right)\vee \left(IN\leq ECT+\right)\;to\;\exists AS\wedge \exists EX$ , signifying that IN terminates leading to EX , while the AS persists throughout the entire interval. From t=15h until the end of therapy, in most of the tissue area, $C_{1}$ increases and $\left(IN\geq AS\right)\vee \left(IN\geq ECT+\right)$ , indicating that IN≥ECT+ is untrue, and consequently, IN≥AS is true, which is aligns with findings in the TPK profile for E=46 kV/m and E=0 kV/m .

For a blood velocity of $\lambda_{inl}=0.001m/s$ , Figure 41 of Supplementary Material, the RTMs behavior resembles that observed for E=46 kV/m until t=3h . After t=3h , some differences can be appreciated. The subsequent periods (3–6 h and 6–9 h) indicate that ∃DIS∧∃IN in points near FIB , whereas $C_{1}$ diminishes and $\left(IN\geq AS\right)\vee \left(IN\geq ECT+\right)$ across most of the domain, indicating that internalization occurs at all sites, whereas association does not. Between 9 h and 12 h, it occurs that $\left(EX\leq ECT-\right)\wedge \left(EX\geq DIS\right)\;to\;\exists DIS\wedge \exists IN$ in a certain point near TVWI , which means that externalization stops and internalization begins at a given temporal instant within this interval, but dissociation is consistently present. In the remaining domain during the period (9–12 h), IN≥ECT+ . Between 12h and 15h , it is noted that IN≤ECT+ at all points of the domain until a specific moment within this period; however, certain points near TVWI exhibit that $\left(IN\geq AS\right)\wedge \left(IN\leq ECT+\right)\;to\;\exists AS\wedge \exists EX$ , signifying a transition from internalization to externalization in these points. In contrast to the preceding time period, $C_{1}$ decreases. During the following time periods (15–18 h and 18–21 h), $C_{1}$ increases and $\left(IN\geq AS\right)\vee \left(IN\geq ECT+\right)$ in most points, suggesting that IN≥ECT+ is not feasible, thus affirming that IN≥AS holds true. From t=21h to t=24h , $C_{1}$ diminishes, and $\left(EX\leq ECT-\right)\wedge \left(EX\geq DIS\right)$ in most of the tissue; that is, during the last 3 hours of therapy, externalization happens in nearly all tissue locations.

For a velocity of $\lambda_{inl}=0.01\;m/s$ (Figure 42 of Supplementary Material), the RTMs behavior is consistent with that observed at E=46 kV/m and E=0 kV/m until t=9h . Between 9 h and 12 h, the condition $\left(IN\geq AS\right)\vee \left(IN\geq ECT+\right)$ holds true at each point of the domain until a specific time instant, which leads to the fact that $\left(IN\geq AS\right)\vee \left(IN\geq ECT+\right)\;to\;EX\leq ECT-$ in certain areas next to TVWI , indicating that IN and ECT+ are replaced by opposing mechanisms. During the period from 12 h to 15 h, IN≤ECT+ is observed throughout every domain region up to a specific time instance, from which it becomes evident that $\left(IN\geq AS\right)\wedge \left(IN\leq ECT+\right)\;to\;\exists AS\wedge \exists EX$ at certain locations throughout TVWI , indicating a shift from internalization to externalization. From t=15h to t=21h , RTMs exhibits identical behavior as from 9h to 12h , excluding certain points in proximity to TVWI . During the period from 21 h to 24 h, $\left(IN\geq AS\right)\vee \left(IN\geq ECT+\right)$ is prevalent in most of the subject areas; however, in certain locations close to TVWI , it happens that EX≤ECT− .

##### 5.3.1.2 Spatial and temporal dynamics of internalization and/or externalization rates

Figure 28 of Supplementary Material shows that for a blood velocity of $\lambda_{inl}=0.0001m/s$ , internalization prevails at all time instants ( $C_{2}/C_{1}$ is lower than 1 in most of points), with only several locations near the TVWI exhibiting externalization during the final time intervals. The peak internalization rates (*IN*) emerge during the initial hour, coinciding with the lowest values of $C_{2}/C_{1}$ across the entire time window, consistent with the findings at E=46 kV/m and E=0 kV/m . Furthermore, at E=46 kV/m , IN is consistently lower at TVWI bottom across every time instant. Now, the difference between ${\left(C_{2}/C_{1}\right)}_{\max}$ and ${\left(C_{2}/C_{1}\right)}_{\min}$ is progressively increasing, signifying a reduction in the radial uniformity of IN . For a blood velocity of $\lambda_{inl}=0.001m/s$ , Figure 29 of Supplementary Material, internalization occurs throughout most of the tissue, except for certain areas near the TVWI boundary at specific time points. As for E=0kV/m and E=46kV/m , the maximum internalization degree ( IN ) is observed during the initial hour. In addition, IN is consistently lower in TVWI across each time instants. The minimum velocity ( $\lambda_{inl}=0.0001m/s$ ) shows that incremental difference between ${\left(C_{2}/C_{1}\right)}_{\max}$ and ${\left(C_{2}/C_{1}\right)}_{\min}$ reflects a decrease in the radial uniformity of IN . For $\lambda_{inl}=0.01m/s$ , Figure 30 of Supplementary Material shows that the behavior of ratio $C_{2}/C_{1}$ is comparable to such of the preceding velocity ( $\lambda_{inl}=0.001m/s$ ), which means that the blood velocity has no relevant influence on IN for these two cases.

The rate of internalization ( IN ) exhibits non-uniformity along the axial direction for the velocity of $\lambda_{inl}=0.0001m/s$ . However, for the velocities of $\lambda_{inl}=0.001m/s$ and $\lambda_{inl}=0.01m/s$ , the $C_{2}/C_{1}$ contours exhibit small horizontal deviations, indicating that longitudinal uniformity of *IN* improves with the blood velocity.

##### 5.3.1.3 Spatial and temporal dynamics of association and/or dissociation rates

Figure 31 of Supplementary Material indicates that the $C_{3}$ behavior for $\lambda_{inl}=0.0001m/s$ resembles that observed with E=46kV/m . That is, association predominates throughout the treatment, with $C_{3}$ attaining its lowest value in FBV and its highest value in TVWI , and the degree of association ( AS ) is maximum in TVWI . However, it is important to highlight that after 24 h, $C_{3}$ is higher for E=70kV/m than for E=46kV/m and E=0kV/m in almost all tissue points. For blood velocity of $\lambda_{inl}=0.001m/s$ , Figure 32 of Supplementary Material, the change in $C_{3}$ across the entire domain over time is non-monotonic, suggesting alternating phases of dissociation and association. As with E=46kV/m , the $C_{3}$ largest values are found in the TVWI lower section, whereas the smallest values are observed along the FBV . The difference between ${\left(C_{3}\right)}_{\max}$ and ${\left(C_{3}\right)}_{\min}$ diminishes over the time after t=0.5h , which suggests that AS is lower in TVWI than FBV throughout most of the therapy. For blood velocity of $\lambda_{inl}=0.01m/s$ , Figure 33 of Supplementary Material shows that $C_{3}$ increases with time, suggesting association, and is notably highest in the lower section of the TVWI . Additionally, the decrease in the difference between ${\left(C_{3}\right)}_{\max}$ and ${\left(C_{3}\right)}_{\min}$ after t=0.5h indicates that AS is reduced in TVWI throughout most of the therapy.

The tissue that has not undergone electroporation demonstrates that the axial uniformity of $C_{3}$ is aversively influenced by the application of an electric pulse across three blood velocities ( $\lambda_{inl}=0.0001m/s$ , $\lambda_{inl}=0.001m/s$ , and $\lambda_{inl}=0.01m/s$ ), representing that the dissociation/association rates vary along the bloodstream. Consistent with prior findings, the $C_{3}$ maximum values are achieved when the velocity is at the middle level, $\lambda_{inl}=0.001\;m/s$ .

#### 5.3.2 Uniform profile (UPK)

##### 5.3.2.1 Interaction of mechanisms of transport and reaction (RTMs).

For a blood velocity of $\lambda_{inl}=0.0001m/s$ , Figure 43 of Supplementary Material, the RTMs behavior is consistent with the observations for case with E=46kV/m at the specified time intervals from 0 h to 0.5 h, 1 h–9 h, and 15 h–24 h. For the other time intervals, noticeable variations may be observed with respect to E=46kV/m . Accordingly, from 0.5 h to 1 h, $\left(IN\leq AS\right)\vee \left(IN\leq ECT+\right)$ is present, and $C_{1}$ increases in most of the tissue area; from 9 h to 12 h, *IN ≥ AS* is present, and $C_{1}$ decreases. Between 12 h and 15 h, $\left(IN\leq AS\right)\vee \left(IN\leq ECT+\right)$ , and $C_{1}$ increases. Regarding other two velocities ( $\lambda_{inl}=0.001m/s$ and $\lambda_{inl}=0.01m/s$ ), the behavior of the RTMs changes with respect to E=46kV/m at the same time periods of the previous blood velocity ( λ=0.0001m/s ), see Figures 44, 45 of Supplementary Material. Between 0.5 h and 1 h, $\left(IN\leq AS\right)\vee \left(IN\leq ECT+\right)$ for both velocities ( $\lambda_{inl}=0.001m/s$ and $\lambda_{inl}=0.01m/s$ ). Between 9 h and 12 h, IN≥ECT+ for $\lambda_{inl}=0.001m/s$ , and IN≥AS for $\lambda_{inl}=0.01m/s$ . Between 12 h and 15 h, IN≤ECT+ for $\lambda_{inl}=0.001m/s$ , and $\left(IN\leq AS\right)\vee \left(IN\leq ECT+\right)$ for $\lambda_{inl}=0.01m/s$ .

##### 5.3.2.2 Spatial and temporal dynamics of externalization and/or internalization rates

For $\lambda_{inl}=0.0001m/s$ , Figure 34 of Supplementary Material, internalization exists during the treatment, as for E=46 kV/m and E=0 kV/m , and the IN rate is lower near the TVWI inferior zone, except at t=12h where IN values are smaller along the FBV . In this scenario, the highest IN is observed during the initial hour of treatment, and the comportment of the change between ${\left(C_{2}/C_{1}\right)}_{\min}$ and ${\left(C_{2}/C_{1}\right)}_{\max}$ shows no signs of monotony, that is, radial uniformity of IN can increase or decrease over the time. For the two additional blood speeds, $\lambda_{inl}=0.001m/s$ (Figure 35 of Supplementary Material) and $\lambda_{inl}=0.01m/s$ (Figure 36 of Supplementary Material), the $C_{2}/C_{1}$ ratios behavior is quite similar to the analogous velocities with E=46 kV/m , except at t=12h where there is a significant increase in $C_{2}/C_{1}$ for E=70 kV/m , indicating a significant reduction in the internalization rate ( IN ) throughout the tissue.

The effect of $\lambda_{inl}$ on IN axial uniformity is consistent for both E , 46 kV/m and 0 kV/m ; that is, for $\lambda_{inl}=0.0001m/s$ , this uniformity is clearly affected, whereas the other two blood velocities have a lesser impact.

##### 5.3.2.3 Spatial and temporal dynamics of association and/or dissociation rates

For a blood velocity of $\lambda_{inl}=0.0001m/s$ , Figure 37 of Supplementary Material, the $C_{3}$ value constantly increases, and it reaches its highest value in TVWI and its lowest value in FBV . Additionally, the AS values are maximum along the TVWI . The $C_{3}$ upper limit for this field of electricity size ( E=70 kV/m ) exceeds such as other ones E , ( 46 kV/m and 0 kV/m ). For the blood velocities ( $\lambda_{inl}=0.001m/s$ and $\lambda_{inl}=0.01m/s$ ), Figures 38, 39 of Supplementary Material indicate that $C_{3}$ exhibits behavior analogous to the corresponding blood velocities at E=46 kV/m , albeit with different values. Among the three electric field strengths ( E=70 kV/m , E=46 kV/m , and E=0 kV/m ), the $C_{3}$ largest values occur when E=70 kV/m .

The $\lambda_{inl}$ influence on the $C_{3}$ axial uniformity is insignificant for this UPK profile. The $C_{3}$ maximum values are still achieved for the middle blood speed, $\lambda_{inl}=0.001m/s$ .

#### 5.3.3 Comparison with recent literature of tissues electroporated above the reversible electric field

Recent studies have examined the effects of electric pulses exceeding the reversible electric field on intracellular and extracellular concentrations (Mondal et al., 2021; Mondal et al., 2023), yielding results that are qualitatively consistent with the current investigation. As noted in Mondal et al. (2023), an increase in the electric field magnitude ( E ) beyond the reversible threshold ( 10 kV/m in that work) results in the equilibrium of concentrations ( $\left.C_{2}/C_{1}\right)$ being achieved more rapidly. In that study, a concentration balance between $C_{1}$ and $C_{2}$ was achieved at electric fields of 13 kV/m , 14 kV/m , and 15 kV/m , occurring at 190 s, 75 s, and 20 s, respectively. Under the simulation conditions of the present work, a continuous concentration balance could not be achieved, likely due to protein association, which was neglected in [102]. However, for E=70 kV/m , partial equilibrium conditions ( $\left.C_{2}/C_{1}=1\right)$ are attained for the TPK profile from 18 h until the conclusion of the treatment at certain points near the TVWI (Figures 28–30 of Supplementary Material). In the case of E=46 kV/m (Figure 13 of the manuscript and Figures 16 and 17 of Supplementary Material), partial equilibrium conditions are not attained for TPK , indicating that an increase in E beyond the reversible threshold ( E=46 kV/m in this case) can facilitate this concentration balance. On the other hand, the impact of pulse strengths exceeding the reversible threshold on both extracellular and intracellular concentrations was examined in [103] utilizing a single cell-electroporation model. Three levels of electrical field magnitude ( 15 kV/m,25 kV/m,and 40 kV/m ) and two pulse spacing intervals (50 s and 100 s) were examined within a time window of 1,000 s. In the referenced study, for a constant pulse spacing *(* $d_{pulses}$ *)*, an increase of the electric field ( E ) by 1.67 times (from 15 kV/m to 25 kV/m ) and 1.60 times (from 25 kV/m to 40 kV/m ) was reported, leading to average intracellular concentration increases of 17 and 2 times, respectively. Conversely, when maintaining a constant electric field magnitude $\left(E\right)$ and reducing the pulse spacing by a half (from 100 s to 50 s), the average intracellular concentration increased by only 1.2 times. This indicates that the electric field magnitude ( E ) exerts a more significant influence on the internalization rate ( IN ) than the pulse spacing *(* $d_{pulses}$ *)*. Further research could corroborate this with the model developed in the current study.

## 6 Clinical implications and conclusion

This investigation examined the impact of the combination of inlet blood velocity ( $\lambda_{inl}$ ) and electric field strength ( E ) regarding the presence, comparative significance, and rates of transport and reaction mechanisms ( RTMs ) arising in therapies with electricity and chemotherapy. Two dissimilar pharmacokinetic profiles were compared: tri-exponential ( TPK ) one-short, where drug concentration decreases exponentially after a one-short infusion, and uniform ( UPK ), where drug concentration is maintained at a constant level throughout the treatment. The findings were obtained from simulations of multiple treatment scenarios utilizing a prior developed, tested, and verified *in silico* tool found on the global method of approximate particular solutions ( GMAPS ) (Vélez Salazar and Patiño Arcila, 2023b). The outcomes indicate that RTMs for one-shot tri-exponential ( TPK ) and uniform ( UPK ) profiles may vary for a specific combination of E and $\lambda_{inl}$ . The limit that can be reversed ( E=46 kV/m ) for TPK does not significantly impact the interplay of the RTMs compared to domain that has been no electroporated ( E=0 kV/m ) across the analyzed blood speeds, $\lambda_{inl}=\left[1x10^{-4}m/s,1x10^{-3}m/s,1x10^{-2}m/s\right]$ ; however, this interaction is notably affected by $\lambda_{inl}$ . The internalization rate ( IN ) of drug initially dominates the drug association degree ( AS ) during the first 30 min. Subsequently, from 0.5h to 1h , a reversal in this trend may happen for each value of $\lambda_{inl}$ and both sizes of field of electricity ( E=46 kV/m and E=0 kV/m ). From 1h to the conclusion of the therapy, the RTMs presence and significance are contingent upon blood velocity. Specifically, for $\lambda_{inl}=1\;x\;10^{-4}m/s$ , the internalization degree ( IN ) exceeds the association degree ( AS ). As $\lambda_{inl}=1\;x\;10^{-3}m/s$ , both dissociation and internalization occur, and their interaction remains indeterminate. For $\lambda_{inl}=1x10^{-2}m/s$ , the rate of internalization ( IN ) may dominate the rate of association ( AS ) and/or positive outside-of-cells transport degree ( ECT+ ). The limit of irreversible ( E=70 kV/m ) significantly impacts the RTMs in relation to the tissue that has not been electroporated ( E=0 kV/m ), particularly after t=1h , with this effect being contingent upon $\lambda_{inl}$ . For the lower velocity $\left(\lambda_{inl}=1\;x{\;10}^{-4}m/s\right)$ , IN exceeds AS for most of the treatment across most of the domain. However, from t=9h to t=12h , IN dominates over AS and/or ECT+ , leading to externalization in certain areas near the TVWI . At an intermediate velocity $\left(\lambda_{inl}=1\;x{\;10}^{-3}m/s\right)$ , internalization occurs until t=21h , with exceptions near TWVI where externalization is observed between t=9h and 12 h. Furthermore, periods of dissociation and association may occur from t=1h to t=21h , while positive outside-of-cells transport is predominantly observed from t=3h to t=21h . During the therapy final 3 hours ( t=21h to t=24h ), dissociation, externalization, and negative outside-of-cells transport occur, with the externalization ( EX ) degree being higher than the dissociation rate ( DIS ) and lower than the negative outside-of-cell transport degree ( ECT− ). At the higher velocity $\left(\lambda_{inl}=1\;x\;10^{-2}m/s\right)$ , association, internalization, and positive outside-of-cell transport are the primary RTMs throughout the therapy, while negative extracellular transport and externalization occur only at specific points near TVWI during certain time intervals. In most of the domain, the rate of internalization ( IN ) exceeds association rate ( AS ); however, it may be less than the positive outside-of-cells transport degree ( ECT+ ) conditional on the period.

For profile UPK , the blood velocity $\left(\lambda_{inl}\right)$ and reversible limit ( E=46 kV/m ) can impact the interplay among the RTMs concerning the tissue that was not electroporated. For inlet velocities, $\lambda_{inl}=\left[1x10^{-4}m/s,1x10^{-3}m/s,1x10^{-2}m/s\right]$ and sizes of electric field ( E=46 kV/m and E=0 kV/m )*,* internalization occurs in most tissue locations throughout the entire therapy, analogous to the findings for TPK . However, for the lower speed $\left(\lambda_{inl}=1\;x\;10^{-4}m/s\right)$ , IN≥AS at all time instants for TPK after t=1h , whereas this relationship is reversed between 12 h and 15 h for UPK . For other two speeds ( $\lambda_{inl}=1\;x{\;10}^{-3}m/s$ and $\lambda_{inl}=1\;x\;10^{-2}m/s$ ), the interaction between IN and AS at the initial hour is different for both profiles ( TPK and UPK ), in a manner that IN≤AS along the whole domain for TPK , whereas this relationship is reversed in the right half domain approximately for UPK . In the UPK , association is present for $\lambda_{inl}=1\;x\;10^{-4}m/s$ , whereas dissociation takes place after t=1h for $\lambda_{inl}=1\;x\;10^{-3}m/s$ , being these aspects in agreement with TPK . A common characteristic of the TPK and UPK profiles is that applying an E=70 kV/m modifies the interplay among the RTMs in relation to the limit that can be reversed ( E=46 kV/m ), particularly for $\lambda_{inl}=1\;x\;10^{-3}m/s$ and $\lambda_{inl}=1\;x{\;10}^{-2}m/s$ . At $\left(\lambda_{inl}=1\;x{\;10}^{-4}m/s\right)$ , minor differences are observed in the UPK profile between E=70 kV/m and E=46 kV/m . For E=70 kV/m , notable similarities and variations exist between TPK and UPK . For all velocities examined, common RTMs are observed in the TPK and UPK profiles at specific time intervals, including positive extracellular transport, internalization, and association. However, the negative extracellular transport and externalization that are present in certain regions of the TPK profile are absent in the UPK profile.

Numerical results indicate that, for pharmacokinetic profiles ( UPK and TPK ), the internalization/externalization degrees ( IN/EX ) of radial uniformity in tissues that have not been electroporated decrease with an increase in blood velocity $\left(\lambda_{inl}\;\right)$ . Conversely, the axial homogeneity of internalization IN improves with increasing $\lambda_{inl}$ , demonstrating a beneficial effect on this aspect. The influence of blood velocity $\left(\lambda_{inl}\;\right)$ on the axial and radial uniformity of association/dissociation degrees ( AS/DIS ) is unnecessarily monotonous under the situations examined in this study. These conclusions apply to the electroporated tissues at electric fields of E=70 kV/m and E=46 kV/m , corresponding to both TPK and UPK profiles. The increases of E generally decrease the IN/EX and AS/DIS radial uniformity compared to tissue that has not been electroporated, while the axial uniformity is altered to a lesser degree.

This Boolean model may have biological implications in the advancement of electrochemotherapeutic treatments, as it can aid in defining suitable drug doses, pharmacokinetic profiles, and electroporation parameters. Having a reliable understanding of how the mechanisms of transport and reaction ( RTMs ) interact with each other can help in defining these parameters prior to the implementation of the therapy. When comparing therapies utilizing the same total dosage and drug type, one involving drug internalization ( IN ) throughout the entire tissue as the UPK , and the other allowing for sporadic externalization ( EX ) at certain time points as the TPK , the former may be more beneficial. This is due to its potential to increase fin cell death, as the drug remains with the cells for a longer duration.

The biological processes of protein dissociation ( DIS ) or association ( AS ) are important in therapeutic situations. A significant drug association within the interstitial matrix and blood vessels reduces the extravasation rate ( EV ) and the internalization rate ( IN ), respectively. This suggests that, under these conditions, higher drug dosages or longer treatment periods may be needed to achieve cell death in the desired target tissue. However, association ( AS ) also causes a delay in the lymphatic drainage ( LD ), leading to a prolonged presence of drugs within the tumor and enhancing the effectiveness of the therapy. Moreover, high association in the intracellular environment may enhance therapy efficiency by inhibiting cellular externalization. As expected, protein dissociation ( DIS ) has opposite effects. The protein content in cancer patients’ blood significantly influences the occurrence of therapy-related side effects. Individuals diagnosed with hypoproteinemia have an increased vulnerability to adverse reactions and necessitate reduced medication dosages. Furthermore, these patients exhibit a reduced likelihood of drug interactions, which has significant implications for treatment efficacy.

The outside-of-cells transport ( ECT ) is also relevant in a therapeutic situation. Enabling the adequate delivery of the drug ( ECT+ ) to receptors of cancer cells is beneficial for therapies with antibody-drug conjugate ( ADC ), as the medication is only made available when it is internalized into the cell. Additionally, extracellular transportation ( ECT+ ) to viable cells in the peripherical zone can improve the impact of bystanders. This phenomenon arises when the tumor cells’ mortality is boosted because of a lack of protection and feeding from the adjacent healthy cells. In medical applications, negative outside-of-cells transport ( ETC− ) processes may be advantageous in certain regions of tissue, such as the interface between the vessel wall and tissue, as it can help avert the development of contrary concentration gradients and facilitate the interstitial pressure release.

Any parameter grouping that enhances the axial and radial uniformity of the rates associated with the mechanisms of reaction and transport discussed leads to more homogenous cell death impacts within the designated tissue. On a clinical level, this is particularly advantageous, especially for tumors with minimal vascularization.

The current model is designed to supplement, rather than replace, the heuristic methods traditionally employed for dosing anticancer medications, such as weight-based dosing, body surface area dosing, fixed-dose prescribing, and AUC-based dosing, among others. Heuristic methods have been delineated based on *in vitro* and *in vivo* studies, as well as clinical trials, considering various factors, including drug type, cancer type and subtype, cancer stage, prior health treatments, current medications, and primarily, patient characteristics (age, weight, height, and health issues, among others). A primary disadvantage of these methods is their inadequate correlation with the pharmacokinetic parameters of chemotherapeutic agents (loading and maintenance doses, administration timing, decay rates, *etc.*), which compromises the precision in predicting drug exposure and clearance, thereby hindering the capacity for therapy individualization. Furthermore, certain heuristic models may inadequately forecast drug clearance in specific instances, leading to potential mistakes in the formulation of chemotherapy schedules based on these methodologies. The current model can predict the spatio-temporal progression of drug concentration in the vessels, extracellular and intracellular spaces, together with the transport and reaction mechanisms of the chemotherapeutic agent in electroporated and vasoconstricted tissues. The current model may facilitate the preliminary assessment of the impact of chemotherapy dosages and schedules on drug distribution, concentration, and exposure within the vessels and tissues during the treatment period before therapy administration.

For instance, let us consider the time evolution of the bound intracellular concentration, $C_{3}$ , which is a relevant parameter related to the cytotoxicity of the chemotherapeutic treatments (Hubbard et al., 2017; Groh et al., 2014). Each figure considers the time behavior of $C_{3}$ for the four extreme points of the computational domain represented in Figure 16. The difference between the curves of Points 1 and 3 and Points 2 and 4 stands for the longitudinal uniformity of drug internalization, whereas the difference between the curves of Points 1 and 2 and Points 3 and 4 stands for the radial uniformity. The closer the respective curves, the more uniform the drug uptake into the cell. For any combination of E and $\lambda_{inl}$ , there is a noticeable difference between TPK and UPK . While a steep increase of $C_{3}$ at the first time instants is present in TPK to then diminish the time rate of $C_{3}$ , the slope of $C_{3}\;vs.\;t$ curves is practically constant in UPK , except for E=70 kV/m , where skips corresponding to the ends of the electroporation protocols are observed (here, number of electroporation protocols is $N_{ep}=6$ ). For the lower inlet flow velocity $\left(\lambda_{inl}=1\times 10^{-4}\;m/s\right)$ , the concentration curve corresponding to Point 1 (inlet point of the vessel, see Figure 6) is far from the other three curves, as the electric field magnitude $\left(E\right)$ increases for both profiles ( TPK and UPK ), although this effect is more evident for TPK . For the intermediate flow velocity $\left(\lambda_{inl}=1\times 10^{-3}\;m/s\right)$ , the increase of E brings about a distancing of the profiles of Points 1 and 3 (inlet and outlet points of the vessel, see Figure 6), being this still more perceptible for TPK , while profiles of Points 2 and 4 are close to each other. For the larger velocity $\left(\lambda_{inl}=1\times 10^{-2}\;m/s\right)$ , this behavior is maintained, but profiles of Points 1 and 3 get closer to each other. From a clinical perspective, this means that TPK generates considerably higher cytotoxic effects at the beginning of the treatment than UPK , but then these effects are attenuated (important diminution of the slope of $C_{3}\;vs.\;t$ ). On the other hand, the cytotoxicity of the UPK profile tends to be uniform. Additionally, even though the application of the electric field could favor the increase of drug concentration (principally above the reversible threshold, E=46 kV/m ), this also negatively affects the longitudinal and radial uniformity of the drug uptake in the cells. On the other hand, the increase in the inlet velocity, $\lambda_{inl}$ , has positive effects on the longitudinal uniformity of drug internalization, as well as on the radial uniformity to a lesser extent. These aspects should be considered when selecting pharmacokinetic profiles and electrochemotherapy parameters.

It is worth mentioning that a tumor cord approach is employed in the present work, which is a very simplified representation of a real cancer tumor. The main purpose of this approach is to study the drug passage from the bloodstream in the vessel toward the tissue space, assuming an axisymmetric domain with homogeneous porosity, ε . This implies some limitations with respect to a real tumor domain that were not considered: realistic 3D vascular and lymphatic network, tumor heterogeneity due to mutations, deletions, chromosomal rearrangements, among others, specific transport mechanisms arising in the endothelial walls and the cell cytoplasm, changes in drug metabolism by enzymes, or inactivation of the medication by alteration of its molecular characteristics when binding to proteins, among others.

## Data Availability

The original contributions presented in the study are included in the article/Supplementary Material; further inquiries can be directed to the corresponding author.

## Glossary

***C***_***1***_: Extracellular drug concentration
***C***_***2***_: Free intracellular drug concentration
***C***_***3***_: Bound intracellular drug concentration
***C***_***v***_: Drug concentration in the bloodstream
AS: Association rate
***D***: Interstitial diffusion coefficient of tissue
***D***_***0***_: Interstitial diffusion coefficient of tissue (non-electroporated)
**DIE**: Degree of irreversible electroporation
DIS: Dissociation rate
***DOE***: Degree of electroporation
${DOE}_{R}$: Transient degree of electroporation
$d_{pulses}$: Pulse spacing
***E***: Electric field magnitude
***ECM***: Extracellular matrix
ECT **+**: Positive extracellular transport
ECT **-**: Negative extracellular transport
EP: Electroporation
***E***_***rev***_: Reversible threshold of electroporation
***E***_***irrev***_: Irreversible threshold of electroporation
EV: Extravasation rate
EX: Externalization rate
$f_{pulse}$: Pulse frequency
***FEM***: Finite element method
***FVM***: Finite volume method
GET: Gene electro-transfer therapies
***GMAPS***: Global method of approximate particular solutions
ICT: Intracellular transport
IFP: Interstitial fluid pressure
IN: Internalization of drug
***IRE***: Irreversible electroporation
***k***_***1***_: Cell membrane permeability
***k***_***1,max***_: Maximum value of cell membrane permeability
***k***_***1,min***_: Minimum value of cell membrane permeability
***k***_***2***_: Drug association rate (reaction property)
***k***_***-2***_: Drug dissociation rate (reaction property)
***k***_***v,max***_: Maximum value of vessel wall effective permeability
***k***_***v,min***_: Minimum value of vessel wall effective permeability
***k***_***v***_: Effective vessel wall permeability
$k_{vc}$: Vessel wall permeability coefficient
***L***_***cord***_: Length of cord domain
LD: Lymphatic drainage
***m***_***r***_: Coefficient of vasoconstriction
***N***_***ep***_: Number of electroporation treatments
$N_{points}$: Number of collocation points
$N_{pulses}$: Number of consecutive pulses
***p***_***inl***_: Inlet blood pressure
***PK***: Pharmacokinetic profiles
***p***_***p***_: Permeate pressure
***Q***_***leak***_: Leakage flow rate through the vessel wall
***Q***_***inl***_: Inlet flow rate
***r***: Radial coordinate
***r***_***v***_: Vessel radius
$\hat{r}$: Normalized vessel radius
${\hat{r}}_{\min}$: Minimum normalized vessel radius
***R***_***cord***_: Radius of cord domain
***RE***: Reversible electroporation
***RUC***: Representative unitary cell
RTMs: Reaction and transport mechanisms
***S***_***C***_: Concentration scale
***S***_***L***_: Length scale
***S***_***T***_: Time scale
TBV: Transport along the blood vessels
***t***: Time
***t***_***chemo***_: Total time of chemotherapy
***T***_***ep***_: Time of each electroporation treatment
***τ***_***i***_: Decay time coefficient for the permeability (vessel wall or cell membrane)
TMT: Transmembrane transport
***t***_***p***_: Time elapsed from the application of the last pulse
TPK: Tri-exponential one-short profile
***τ***_***r***_: Decay time coefficient of vasoconstriction
TVT: Transvascular transport
***UPK***: Uniform pharmacokinetic profile
V: Voltage
α: Ratio of the total surface area of the cell membrane
$\alpha^{*}$: Sigmoidal function parameter
$\beta^{*}$: Sigmoidal function parameter
$\delta_{1}$: Extracellular volume content
$\delta_{2}$: Intracellular volume content
***σ***: Effective electrical conductivity
***σ***_**max**_: Maximum effective electrical conductivity
***σ***_**min**_: Minimum effective electrical conductivity
***σ***_***irrev***_: Irreversible effective electrical conductivity
***λ***: Blood velocity along the vessel
***λ***_***inl***_: Inlet blood velocity
***μ***: Plasma viscosity
***ω***: Relaxation factor
***Δt***: Time step
