# Supplementary material for: Boolean network and meshless simulations for the comparison of transport and reaction mechanisms arising in one-short tri-exponential and uniform infusion electrochemotherapeutic treatments
Source: Front Bioinform. 2026 Mar 25;6:1719700. doi: 10.3389/fbinf.2026.1719700 (PMC13057505; doi:10.3389/fbinf.2026.1719700)
Supplement: Supplementary file 1 [file DataSheet2.pdf]

SENSITIVITY ANALYSIS OF BOOLEAN MAPS.

The Boolean functions represented in Table 3 of the manuscript depend, among other things, on the ratio C_2/C_1 at a given time instant (t_i), which determines the presence of net internalization (IN) or net externalization (EX) that are mutually exclusive mechanisms from a physical viewpoint. In the equilibrium $(C_2/C_1 = 1)$, none of these mechanisms is present $(\nexists\, EX \wedge \nexists\, IN)$. Near-equilibrium noise can arise when $C_2/C_1 \approx 1$ since space and time fluctuations between IN and EX can occur, leading to different Boolean results. Moreover, the ratio C_3/C_0 also influence these Boolean functions since $C_3/C_0 = 1$ points out that concentration of binding sites has been reached and association is not possible $(\nexists AS)$. Additionally, the time evolution of C_2/C_1, C_3/C_1 and C_3/C_2 also determines the values of the Boolean functions represented in Table 3. Near-equilibrium noise can also be present for subtle time changes of these ratios. To evaluate the sensitivity of the Boolean maps, the ratios C_2/C_1, C_3/C_1 and C_3/C_2 are modified with a prescribed error, ε, as follows:

$$(C_2/C_1)_{mod} = (C_2/C_1).(1+\varepsilon)$$

$$(C_3/C_1)_{mod} = (C_3/C_1).(1+\varepsilon)$$

$$(C_3/C_2)_{mod} = (C_3/C_2).(1+\varepsilon)$$

With ε as an introduce error assuming values of $\varepsilon = [-1\times10^{-3}, 1\times10^{-3}, -1\times10^{-2}, 1\times10^{-2}, -1\times10^{-1}, 1\times10^{-1}]$. Results are presented for $E = 70kV/m$ and summarized in the following tables.

Sensitivity to $\boldsymbol{\varepsilon}$ for $\boldsymbol{E = 70kV/m, TPK}$ and $\boldsymbol{\lambda_{inl} = 0.0001m/s}$.

Reference Case $(\boldsymbol{\varepsilon = 0})$

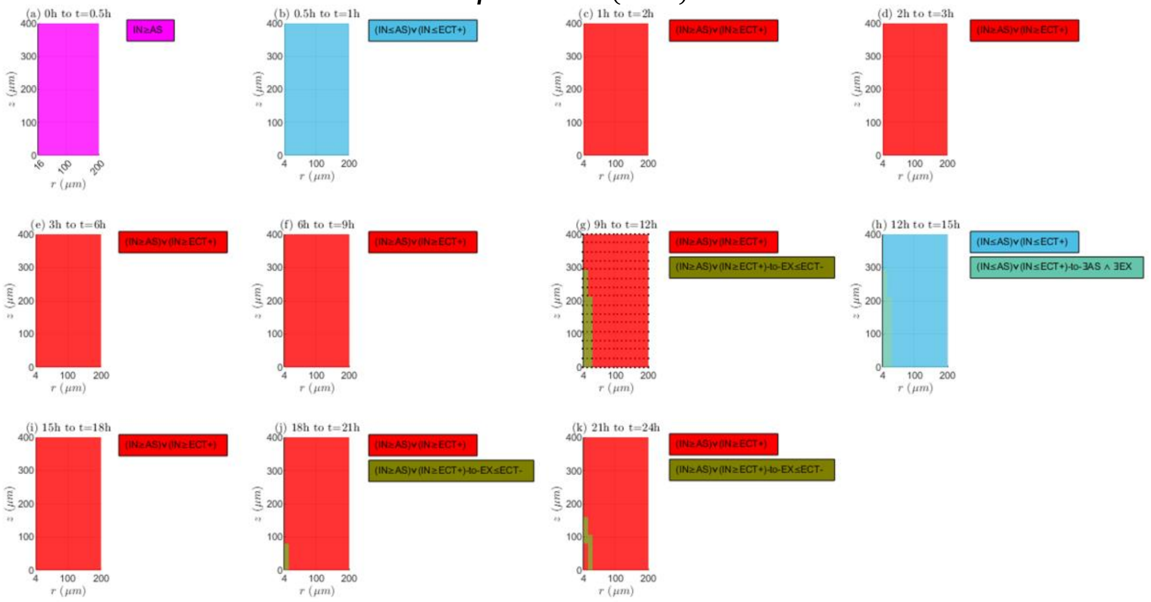

$\boldsymbol{\varepsilon = 1\times10^{-3}}$

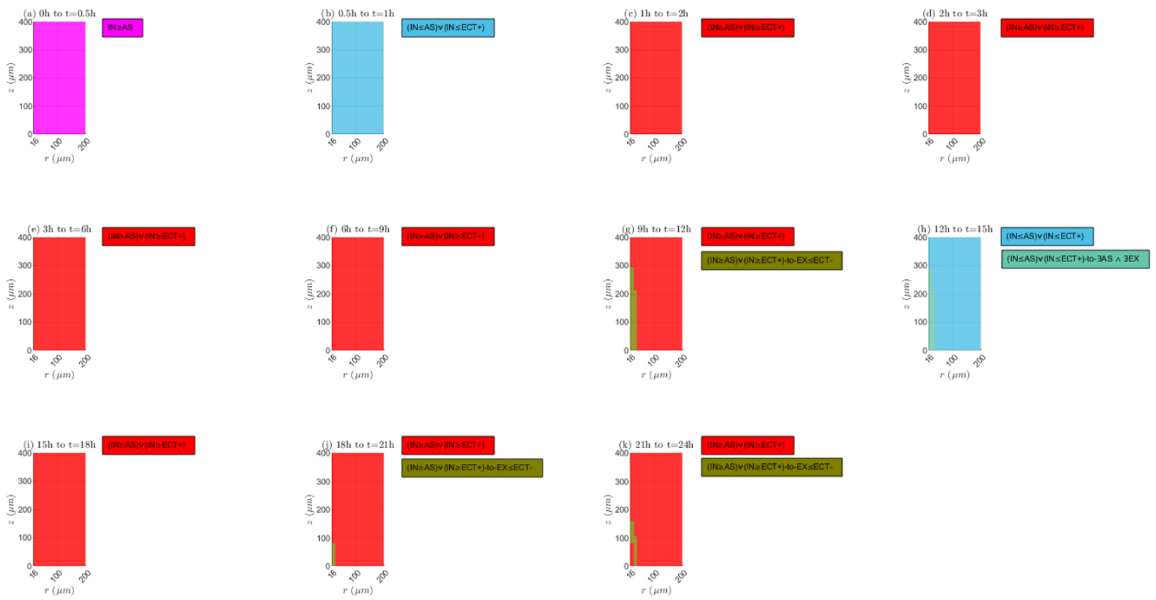

Changes with respect to Reference Case:
Between 9h and 12h, C1 increases

$\boldsymbol{\varepsilon = -1 \times 10^{-3}}$

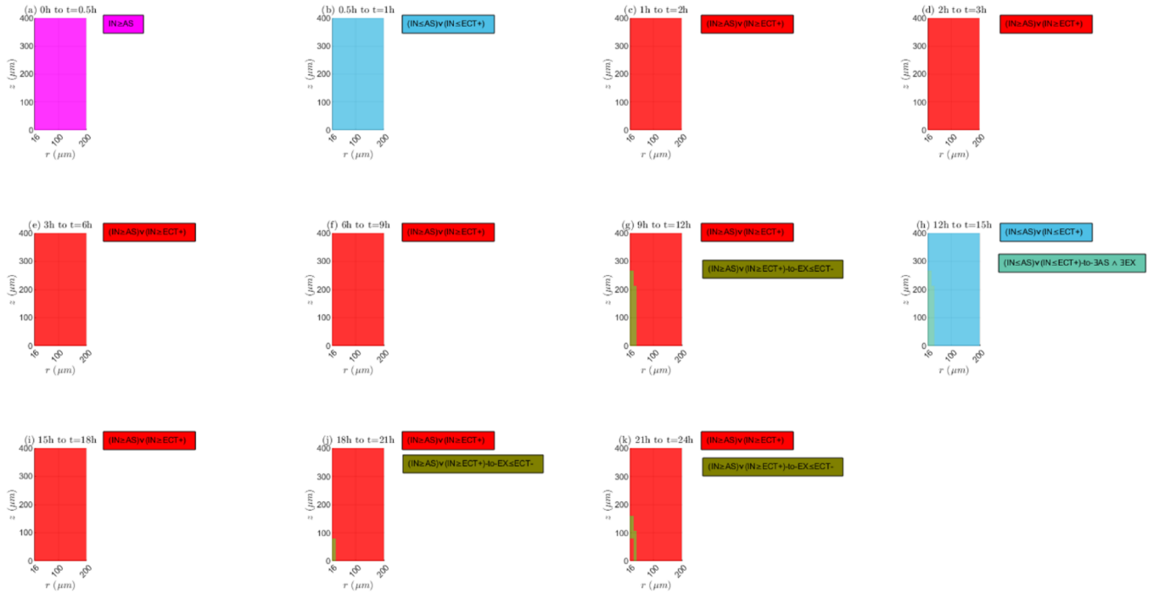

Changes with respect to Reference Case:
Between 9h and 12h, C1 increases

$\boldsymbol{\varepsilon = 1 \times 10^{-2}}$

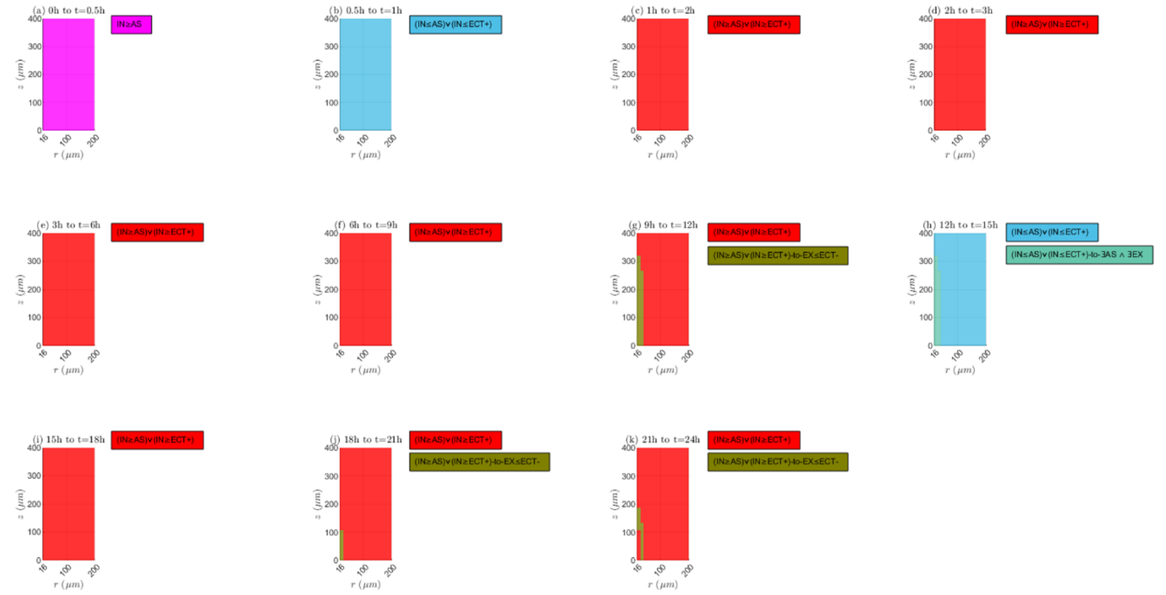

Change with respect to Reference Case:
Between 9h and 12h, C1 increases

$\boldsymbol{\varepsilon = -1 \times 10^{-2}}$

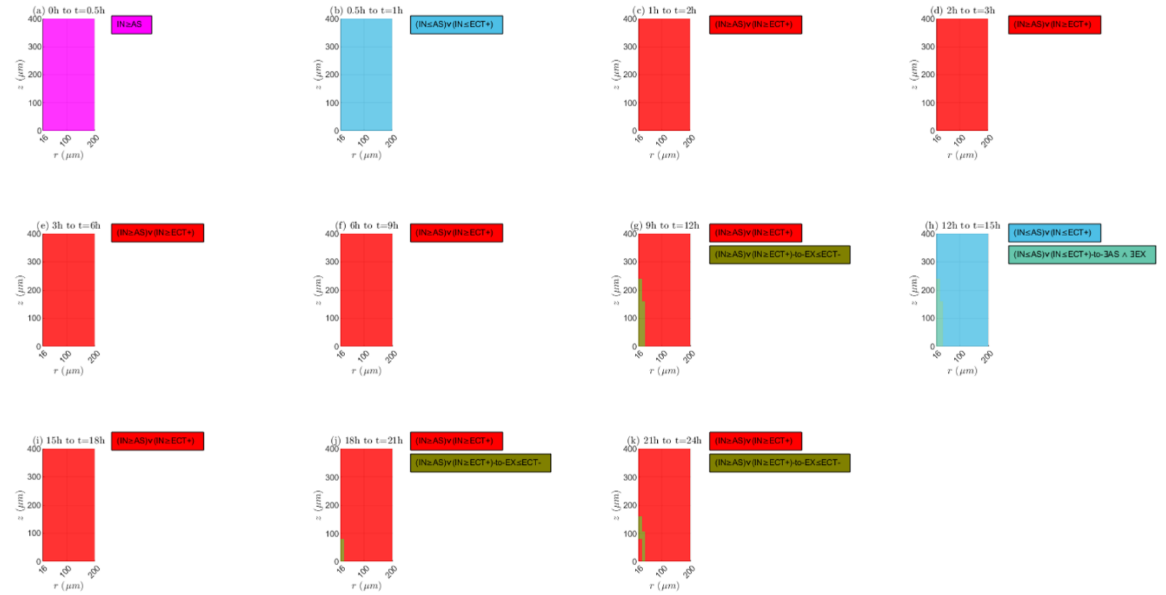

Change with respect to Reference Case:
Between 9h and 12h, C1 increases

$\boldsymbol{\varepsilon = 1 \times 10^{-1}}$

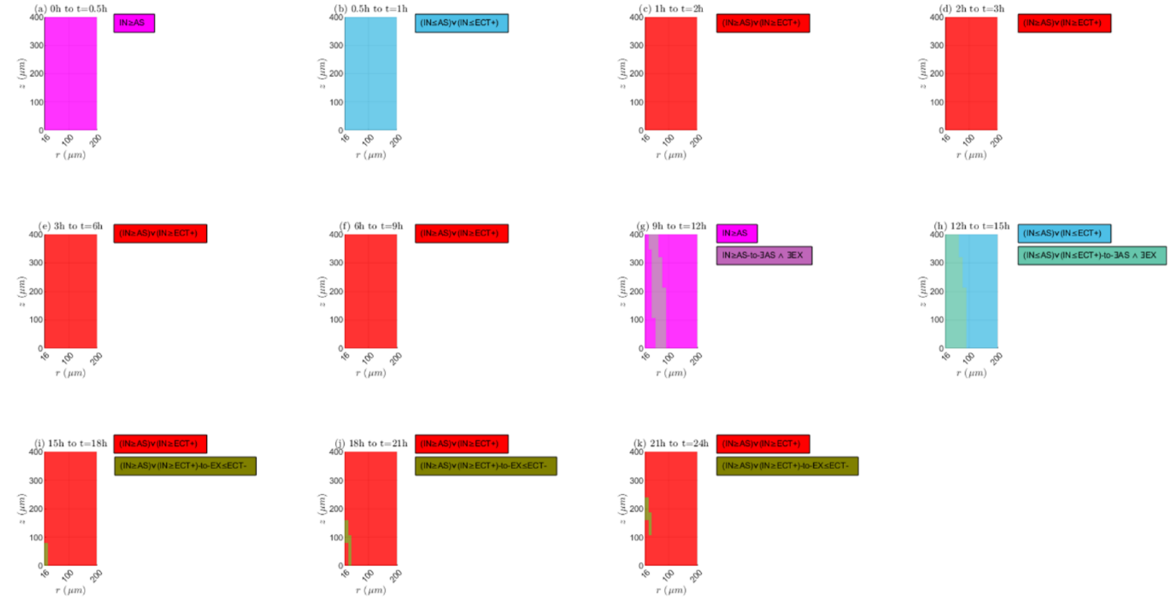

Change with respect to Reference Case:

Between 9h and 12h, $IN > AS$ and $IN > AS\ to\ \exists AS\ and\ \exists EX$, C1 increases

Between 12h and 15h the zone $(IN \leq AS)$ or $((IN \leq ECT+)\ to\ \exists AS\ and\ \exists EX)$ is larger

$\boldsymbol{\varepsilon = -1 \times 10^{-1}}$

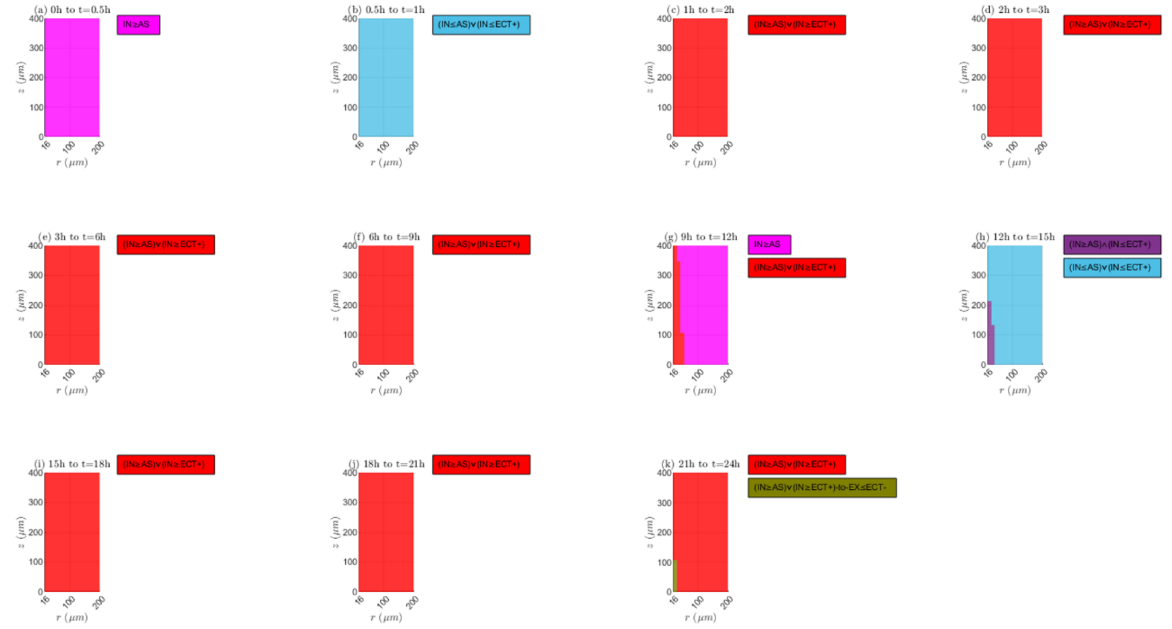

Change with respect to Reference Case:

Between 9h and 12h, $IN > AS$, C1 increases

Between 12h and 15h $(IN \geq AS)$ and $(IN \leq ECT+)$ instead $((IN \leq AS) or (IN \leq ECT+)\ to\ \exists AS\ and\ \exists EX)$

Sensitivity to ε for $E = 70kV/m, TPK$ and $\lambda_{inl} = 0.001m/s$.

Reference Case **($\varepsilon = 0$)**

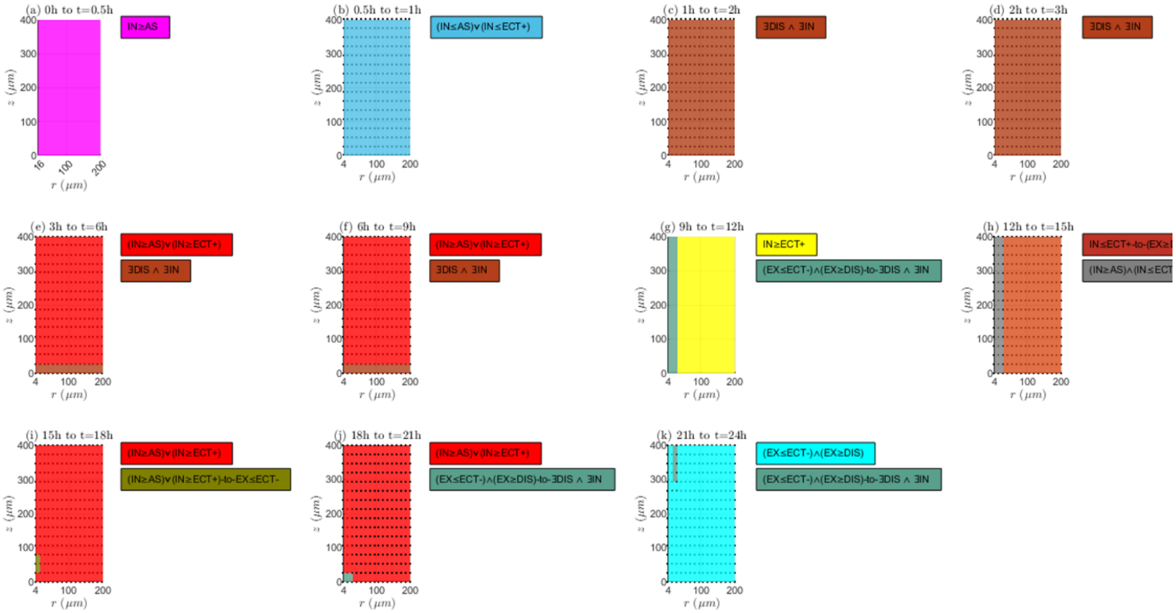

$\varepsilon = 1 \times 10^{-3}$

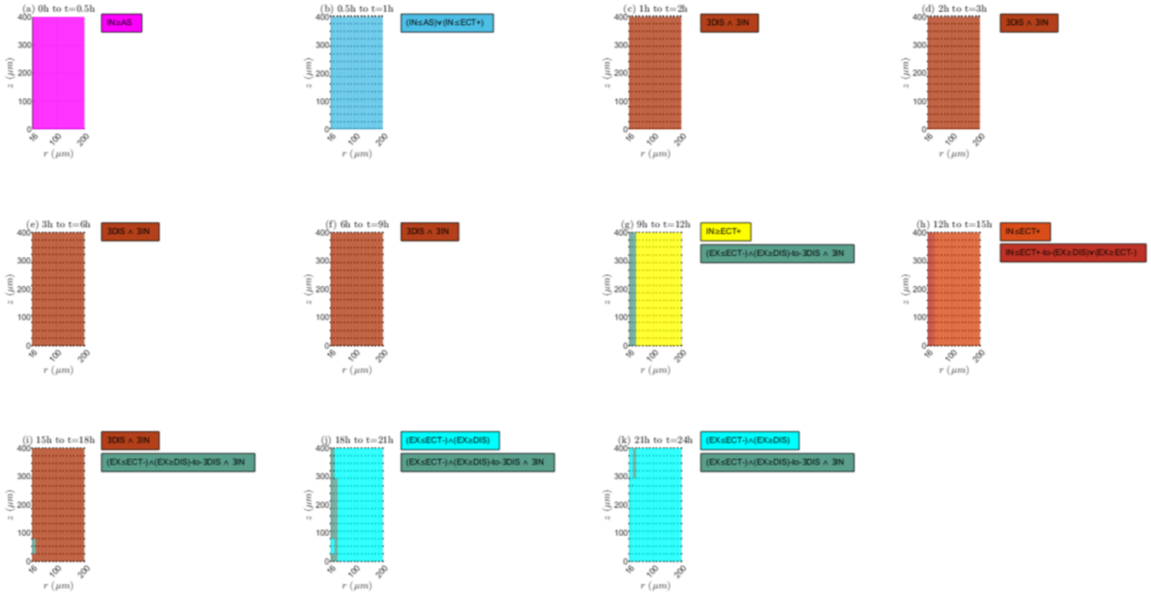

Changes with respect to Reference Case:

Between 3h to 9h $\exists DIS$ *and* $\exists IN$

Between 9h to 12h C1 decreases

Between 12h to 15h $IN \leq ECT+$ instead $IN \leq AS$ *and* $(IN < ECT +)$ *to* $\exists AS$ *and* $\exists EX)$

Between 15h a 18h $\exists DIS$ *and* $\exists IN$ and $(EX < ECT -)$ *and* $(EX \geq ECT +)$ *to* $EX \leq ECT -$

Between 18h to 21h $((EX < ECT -)$ *and* $(EX \geq DIS))$

$(EX < ECT -)$ *and* $(EX \geq DIS)$ *to* $\exists DIS$ $\exists IN$

$\varepsilon = -1 \times 10^{-3}$

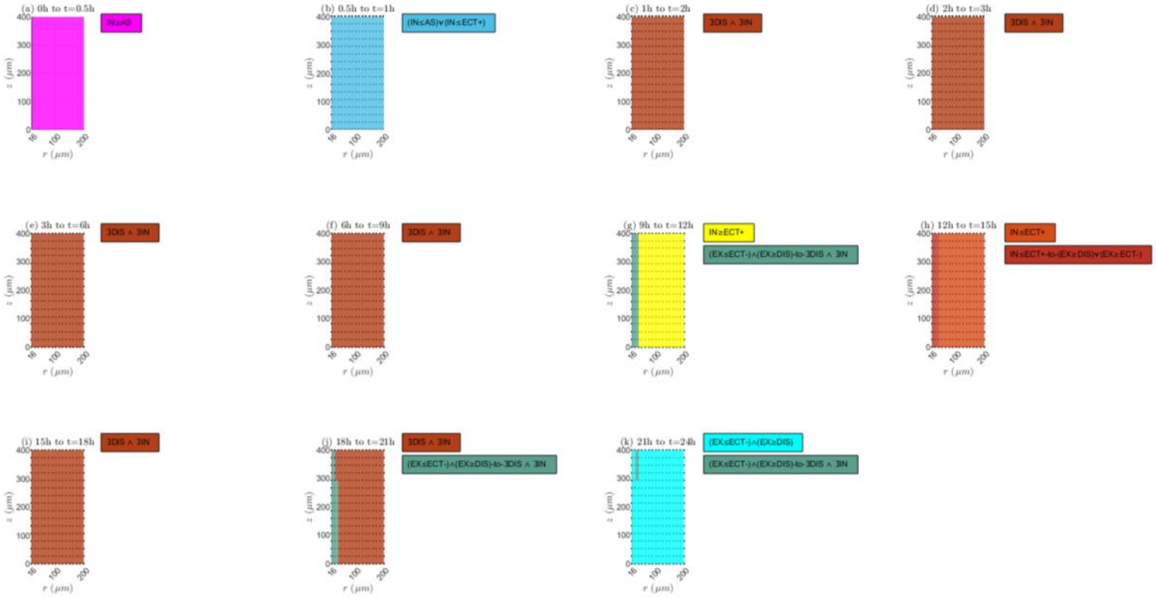

Changes with respect to Reference Case:

Between 3h to 9h $\exists DIS\ and\ \exists IN$

Between 9h to 12h C1 decreases

Between 12h to 15h $IN \leq ECT+$ instead $IN \leq AS\ and\ (IN < ECT+) to\ \exists AS\ and\ \exists EX)$

Between 15h a 18h $\exists DIS\ and\ \exists IN$ and $(EX < ECT-)\ and\ (EX \geq ECT+) to\ EX \leq ECT-$

Between 18h to 21h $((EX < ECT-)\ and\ (EX \geq DIS))$

$(EX < ECT-)\ and\ (EX \geq DIS)\ to\ \exists DIS\ and\ \exists IN$

$\boldsymbol{\varepsilon = 1 \times 10^{-2}}$

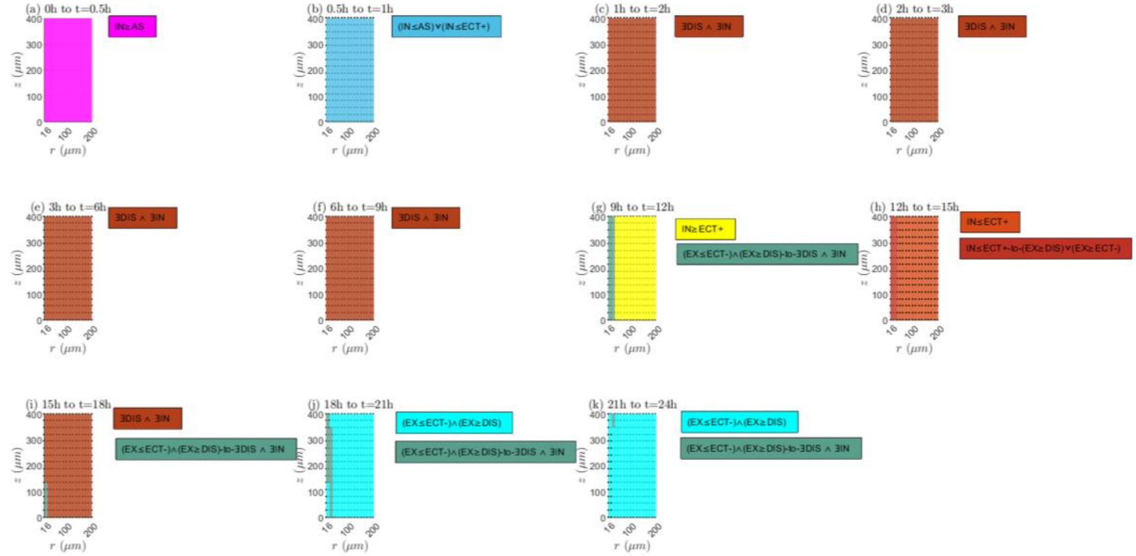

Changes with respect to Reference Case:

Between 3h to 9h $\exists DIS\ and\ \exists IN$

Between 9h to 12h C1 decreases

Between 12h to 15h $IN \leq ECT+$ instead $IN \leq AS\ and\ (IN < ECT+) to\ \exists AS\ and\ \exists EX)$

Between 15h a 18h $(\exists DIS\ and\ \exists IN)$ and $(EX < ECT-)\ and\ (EX \geq DIS) to\ \exists DIS\ and\ \exists INAS$

Between 18h to 21h $((EX < ECT-)\ and\ (EX \geq DIS))$

$(EX < ECT-)\ and\ (EX \geq DIS)\ to\ \exists DIS\ and\ \exists IN$

$\boldsymbol{\varepsilon = -1 \times 10^{-2}}$

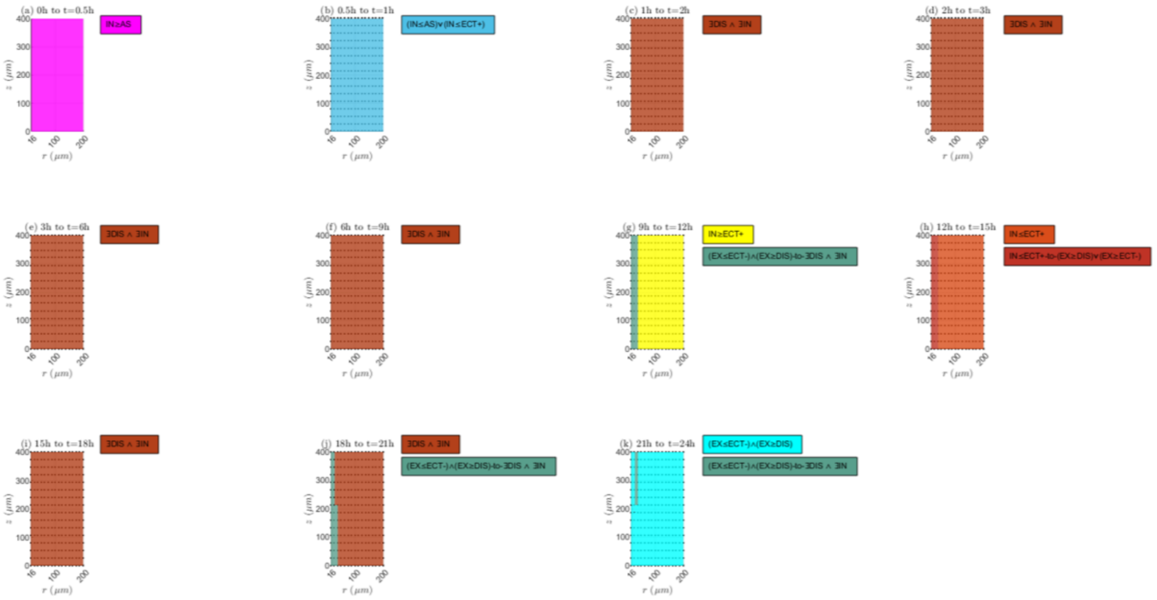

Changes with respect to Reference Case:

Between 3h to 9h $\nexists DIS$ and $\nexists IN$

Between 9h to 12h C1 decreases

Between 12h to 15h $IN \leq ECT+$ instead $IN \leq AS$ and $(IN < ECT+)$ to $\exists AS$ and $\exists EX)$

Between 15h a 18h $(\nexists DIS$ and $\nexists IN)$ and $(EX < ECT-)$ and $(EX \geq DIS)$ to $\exists DIS$ and $\exists IN$

Between 18h to 21h $((EX < ECT-)$ and $(EX \geq DIS))$

$(EX < ECT-)$ and $(EX \geq DIS)$ to $\nexists DIS$ and $\exists IN$

$\boldsymbol{\varepsilon = 1 \times 10^{-1}}$

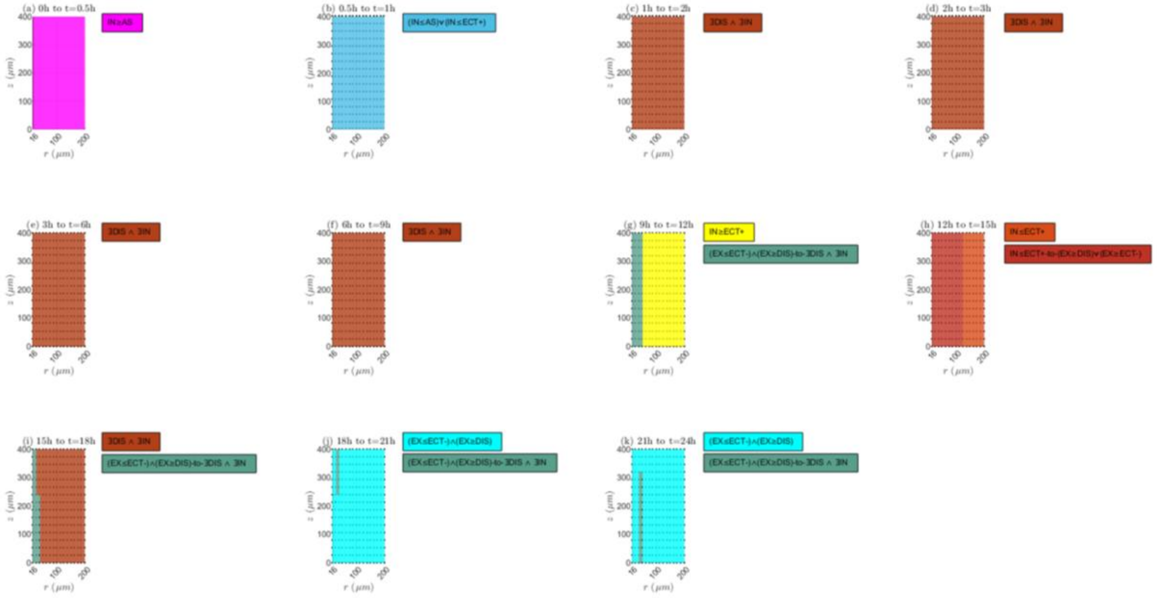

Changes with respect to Reference Case:

Between 3h to 9h $\nexists DIS$ and $\nexists IN$

Between 9h to 12h C1 decreases

Between 12h to 15h $IN \leq ECT+$ and $IN \not\leq ICT+$ to $(EX \geq DIS$ or $EX \geq ECT-)$

Between 15h a 18h $(\nexists DIS$ and $\nexists IN)$ and $(EX \leq ECT-)$ and $(EX \geq DIS)$ to $\exists DIS$ and $\exists IN$

Between 18h to 21h $((EX < ECT-)$ and $(EX \geq DIS))$

$(EX < ECT-)$ and $(EX \geq DIS)$ to $\nexists DIS$ and $\exists IN$

$\boldsymbol{\varepsilon = -1 \times 10^{-1}}$

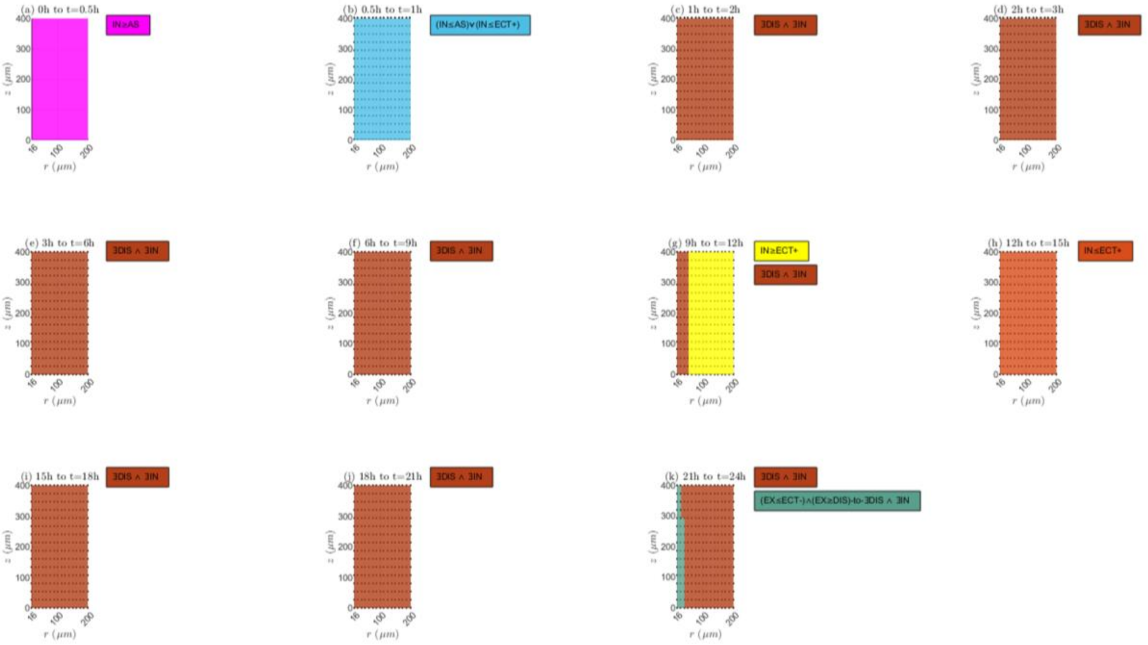

Changes with respect to Reference Case:

Between 3h to 9h $\exists DIS\ and\ \exists IN$

Between 9h to 12h $(IN \geq ECT+)$ $(\exists DIS\ and\ \exists IN)$ C1 decreases

Between 12h to 15h $IN \leq ECT+$

Between 15h a 21h $(\exists DIS\ and\ \exists IN)$

Between 21h to 24h $(\exists DIS\ and\ \exists IN)$ $((EX \leq ECT-)\ and\ (EX \geq DIS)\ to\ \exists DIS\ and\ \exists IN)$

Sensitivity to ε for $E = 70kV/m$, TPK and $\lambda_{inl} = 0.01m/s$.

Reference Case **($\varepsilon = 0$)**

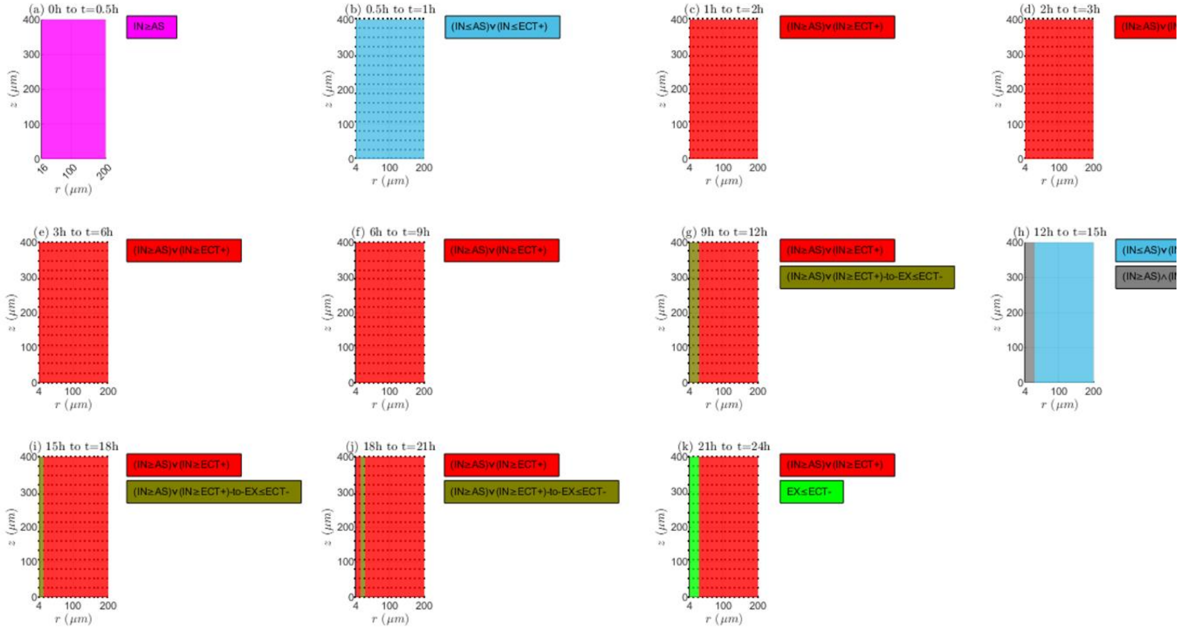

$\varepsilon = 1 \times 10^{-3}$

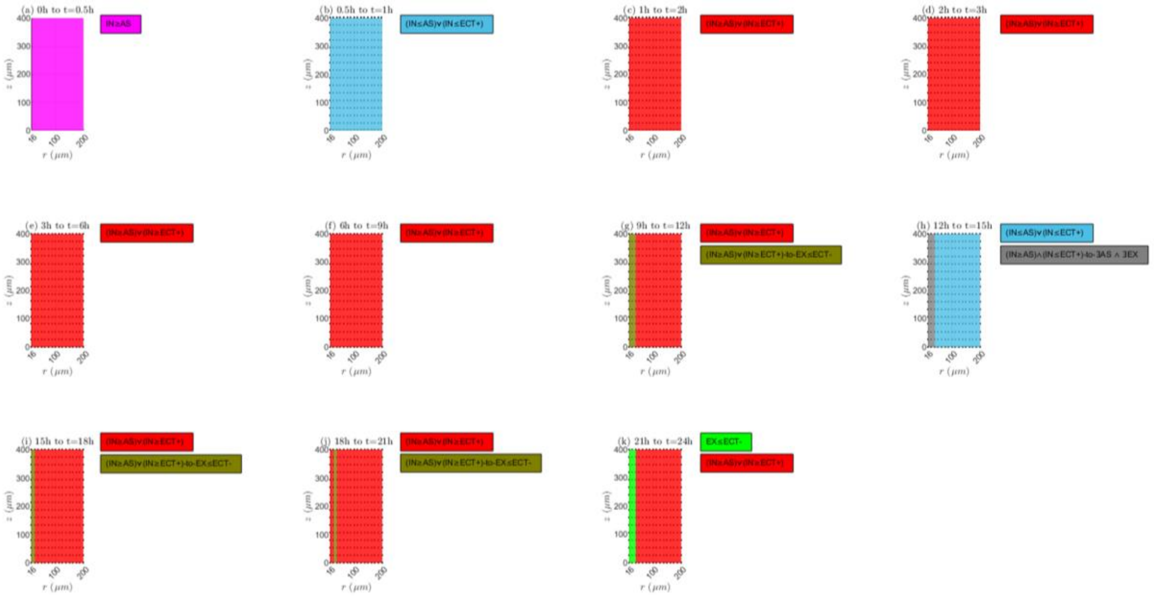

Changes with respect to Reference Case:

There is no changes

$\varepsilon = -1 \times 10^{-3}$

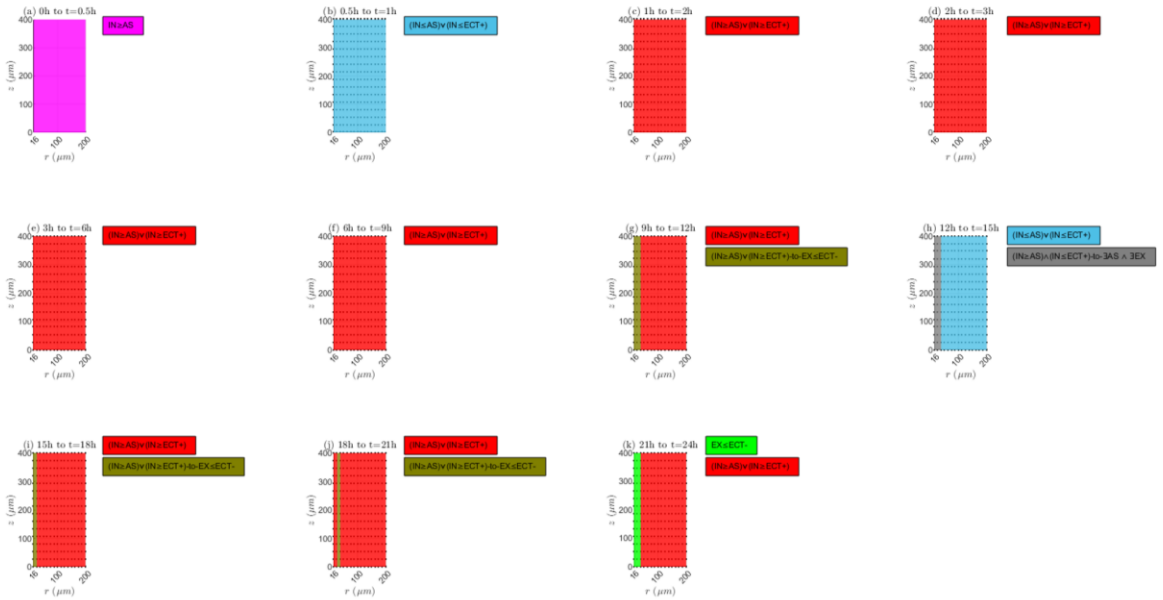

Changes with respect to Reference Case:

Between 12h to 15h $C1$ decreases

$\boldsymbol{\varepsilon = 1 \times 10^{-2}}$

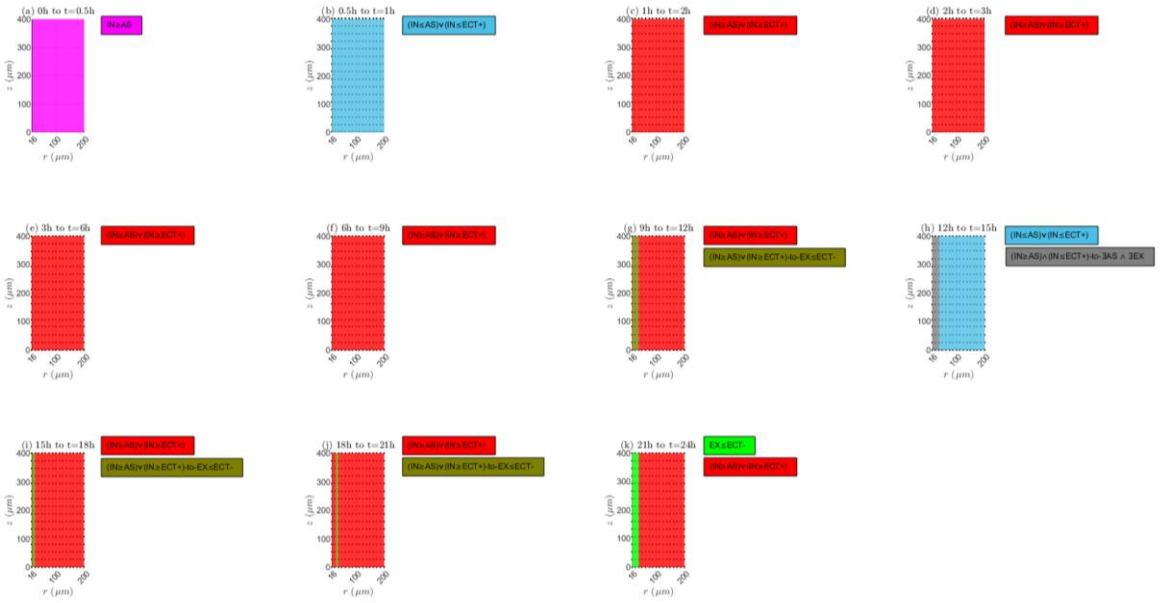

Changes with respect to Reference Case:

Between 12h to 15h $C1$ decreases

$\boldsymbol{\varepsilon = -1 \times 10^{-2}}$

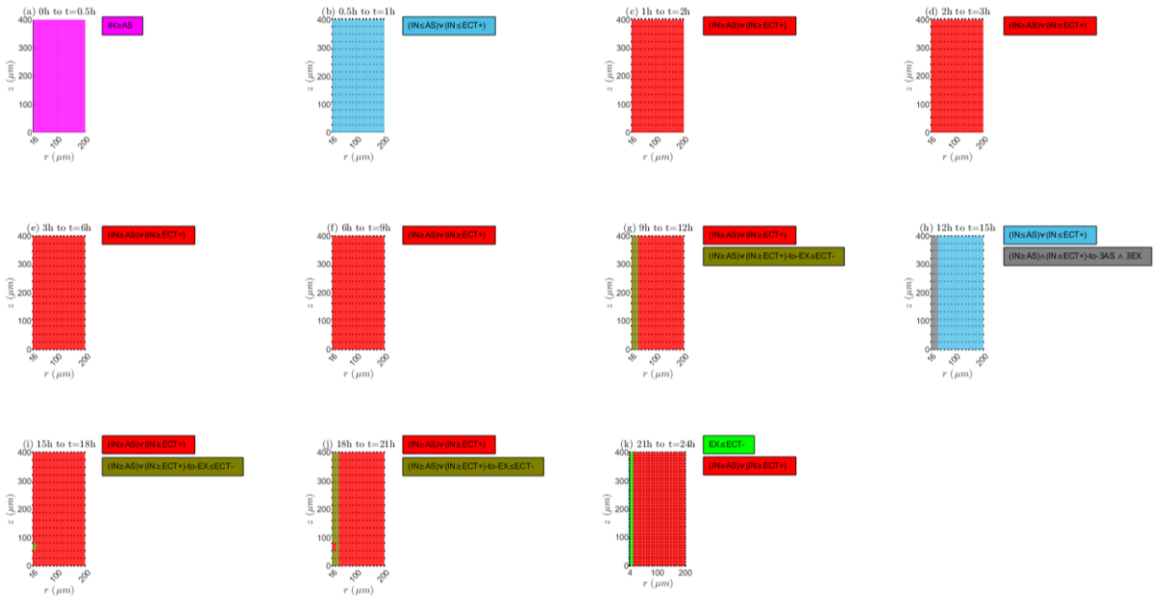

Changes with respect to Reference Case:

Between 12h to 15h $C1$ decreases

Between $(IN{\geq}AS)$ or $(IN{\geq}ECT+)$

$\boldsymbol{\varepsilon} = \mathbf{1} \times \mathbf{10^{-1}}$

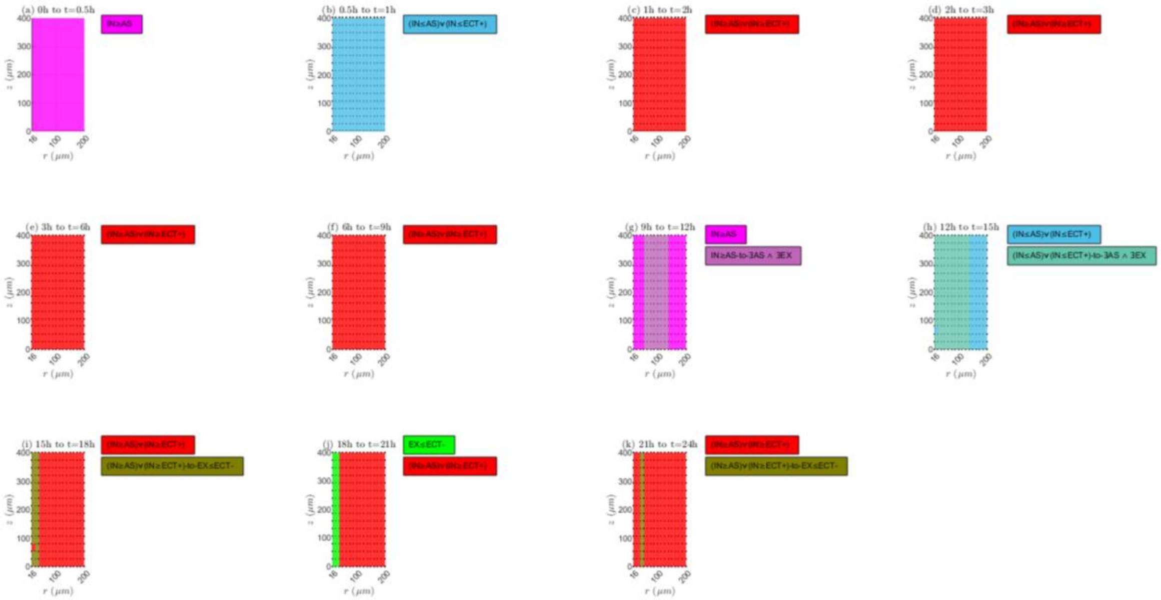

Changes with respect to Reference Case:

Between 9h to 12h $(IN{\geq}AS)$ $(IN{\geq}DIS)$ to $\exists AS$ and $\exists EX)$

Instead $(IN{\geq}AS)$ or $(IN{\geq}ECT+)$ $(IN{\geq}AS)$ $(IN{\geq}AS)$ or $(IN{\geq}ECT+)to$ $EX \leq ECT-)$

Between 12h to 15h $(IN{\leq}AS)$ or $(IN{\leq}ECT+)$ $(IN{\leq}AS)or$ $(IN{\leq}ECT+)$ to $\exists DIS$ and $\exists IN)$

Between 18h a 21h $(EX{\leq}ECT-)$ instead $(IN{\geq}AS)$ or $(IN{\geq}ECT+)to$ $EX \leq ECT-)$

Between 21h to 24h $(IN{\geq}AS)$ $(IN{\geq}AS)$ or $(IN{\geq}ECT+)to$ $EX \leq ECT-)$ instead $(EX{\leq}ECT-)$

$\boldsymbol{\varepsilon} = -\mathbf{1} \times \mathbf{10^{-1}}$

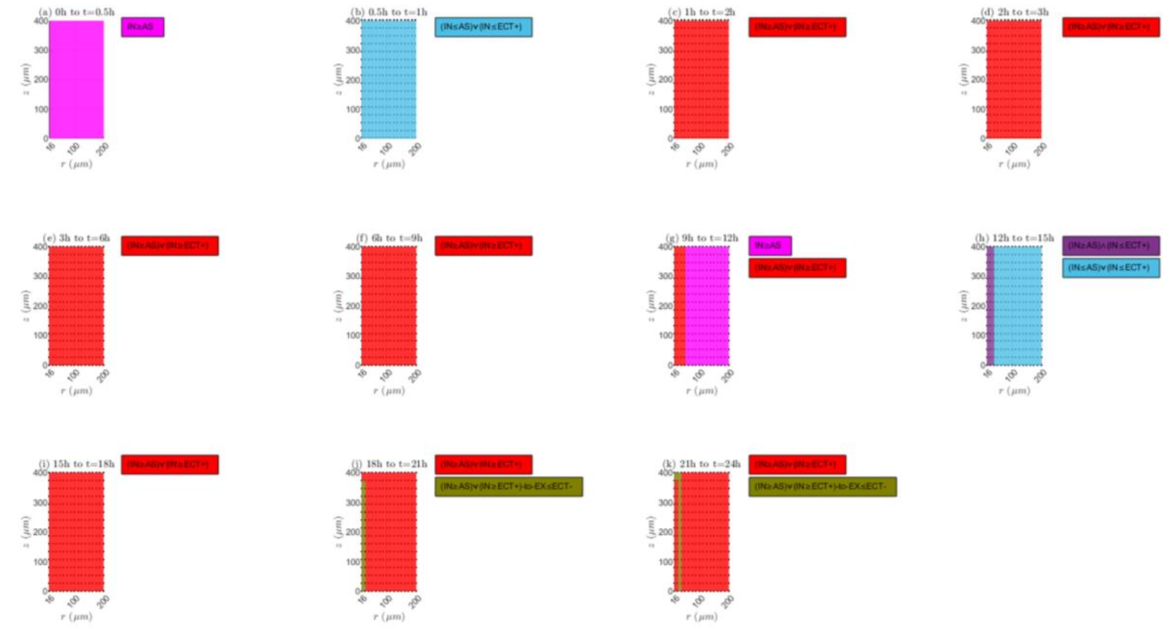

Changes with respect to Reference Case:

Between 9h to 12h $(IN\geq AS)$ $(IN\geq DIS)$ $to\ \exists AS\ and\ \exists EX)$

Instead $(IN\geq AS)$ or $(IN\geq ECT+)$ $(IN\geq AS)$ $(IN\geq AS)$ or $(IN\geq ECT+) to\ EX \leq ECT-)$

Between 12h to 15h $(IN\geq AS)$ and $(IN\leq ECT+)$ instead $(IN\leq AS) or$ $(IN\leq ECT+)$ and C_1 decreases

Between 15h a 18h $(IN\geq AS)$ or $(IN\geq ECT+)$

Between 21h to 24h $(IN\geq AS)$ or $(IN\geq ECT+) to\ EX \leq ECT-)$ instead $(EX\leq ECT-)$

Sensitivity to ε for $E = 70kV/m$, UPK and $\lambda_{inl} = 0.0001m/s$.

Reference Case **($\varepsilon = 0$)**

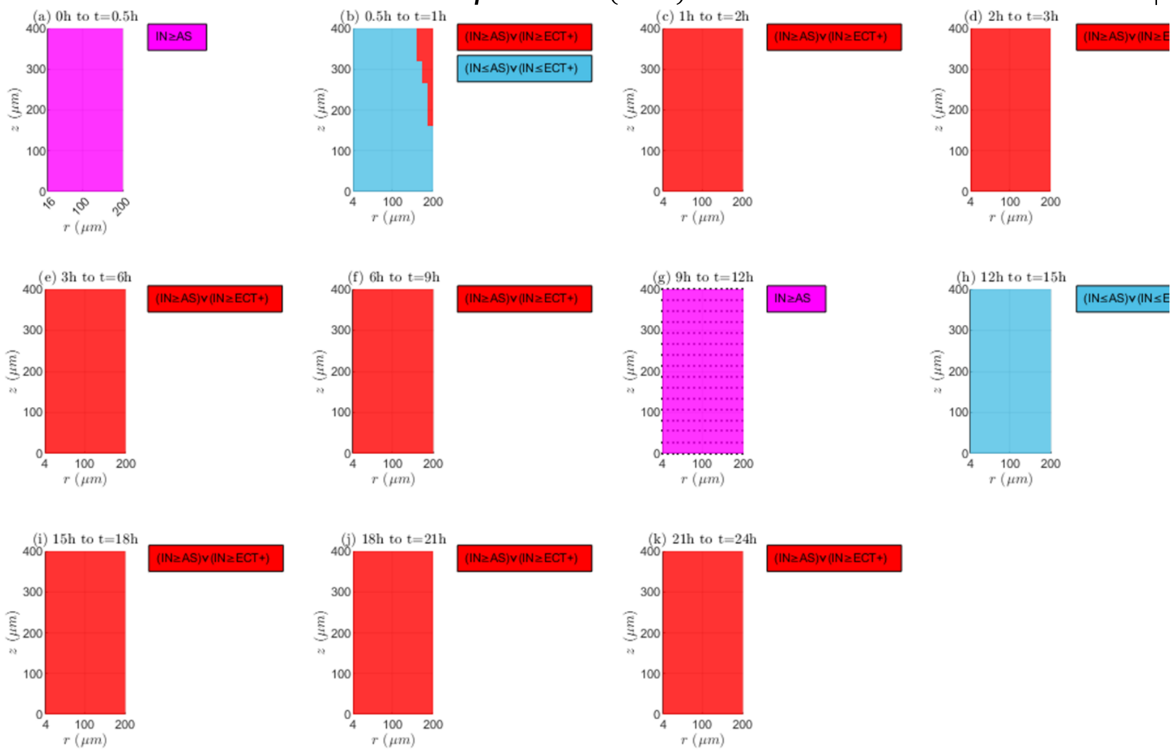

$\varepsilon = 1 \times 10^{-3}$

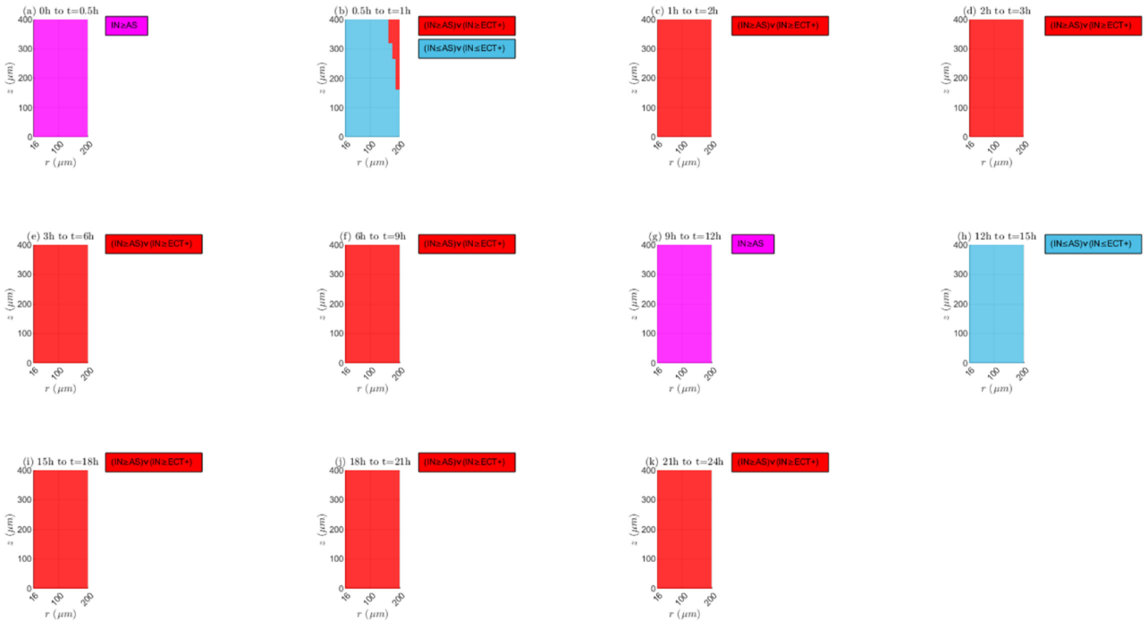

Changes with respect to Reference Case:

Between 9h to 12h C_1 increases

$\varepsilon = -1 \times 10^{-3}$

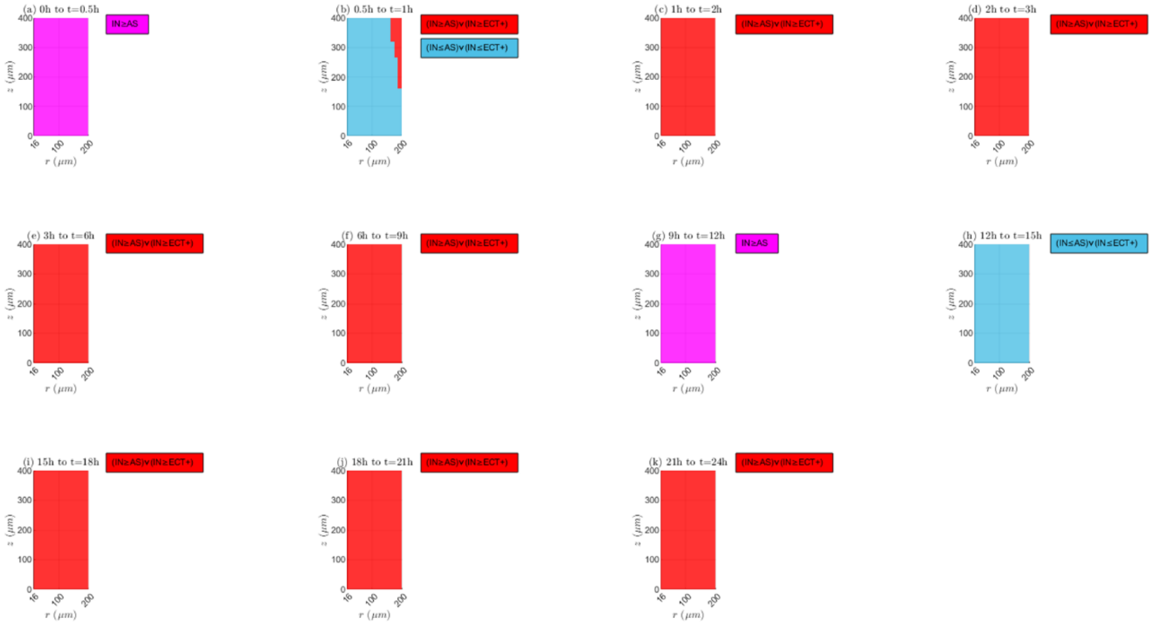

Changes with respect to Reference Case:
Between 9h to 12h C_1 increases

$\boldsymbol{\varepsilon = 1 \times 10^{-2}}$

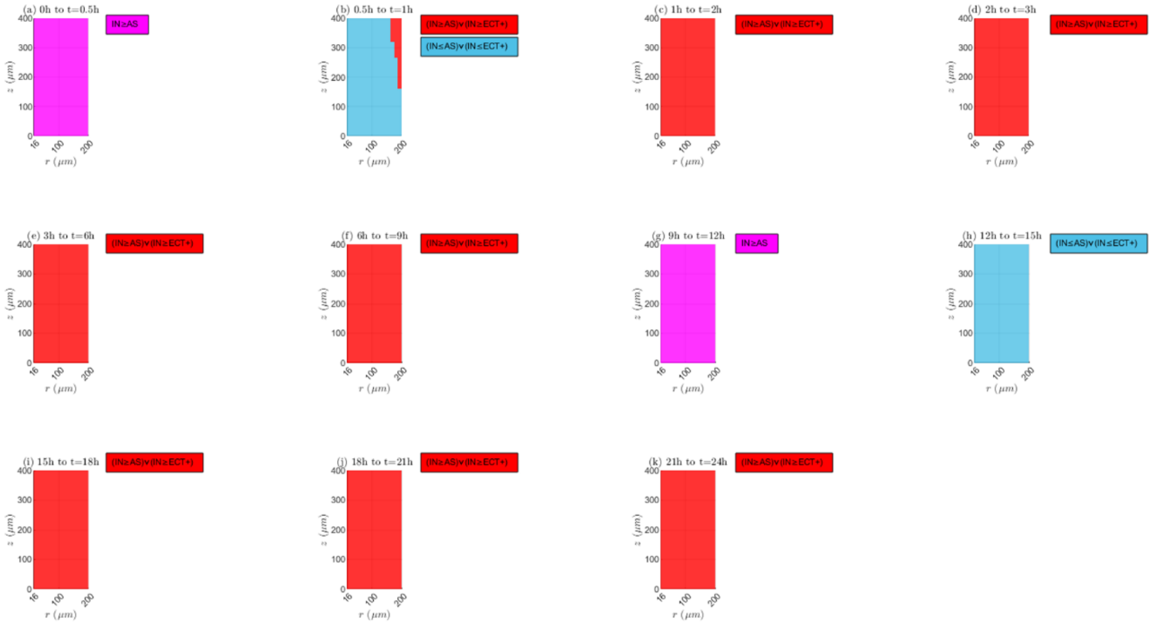

Changes with respect to Reference Case:
Between 9h to 12h C_1 increases

$\boldsymbol{\varepsilon = -1 \times 10^{-2}}$

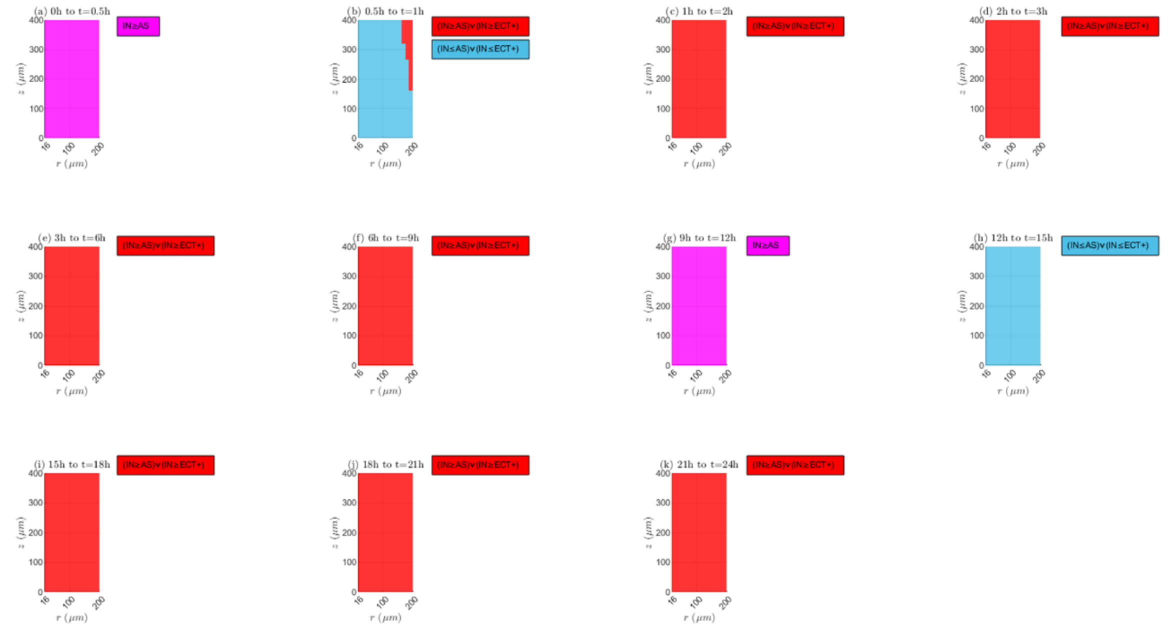

$\boldsymbol{\varepsilon = 1 \times 10^{-1}}$

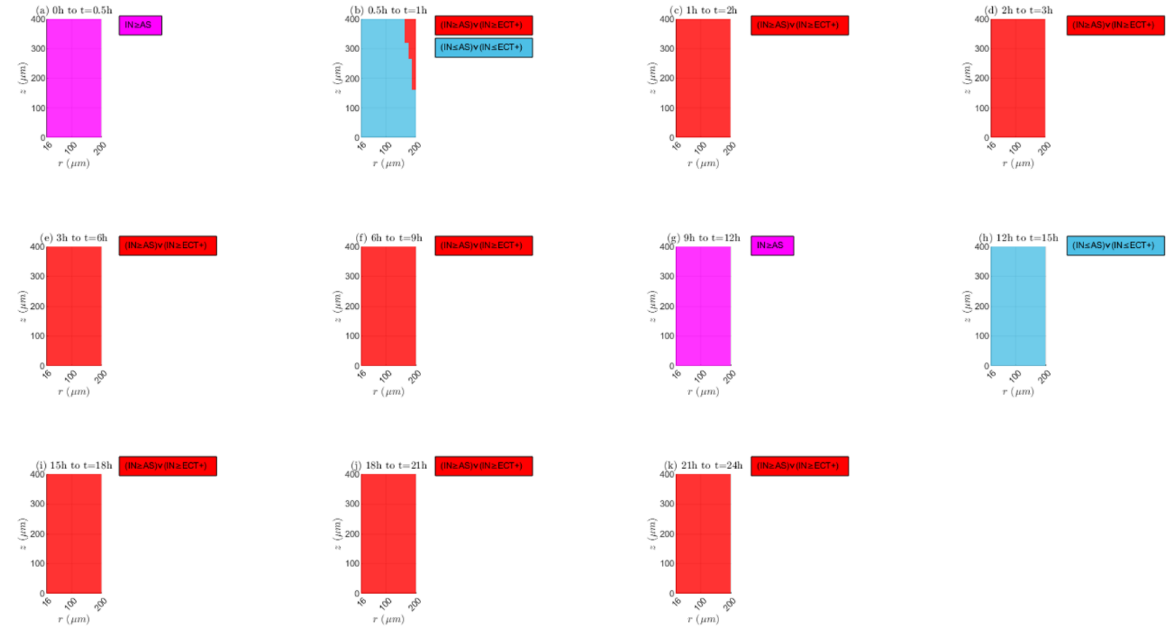

$\boldsymbol{\varepsilon = -1 \times 10^{-1}}$

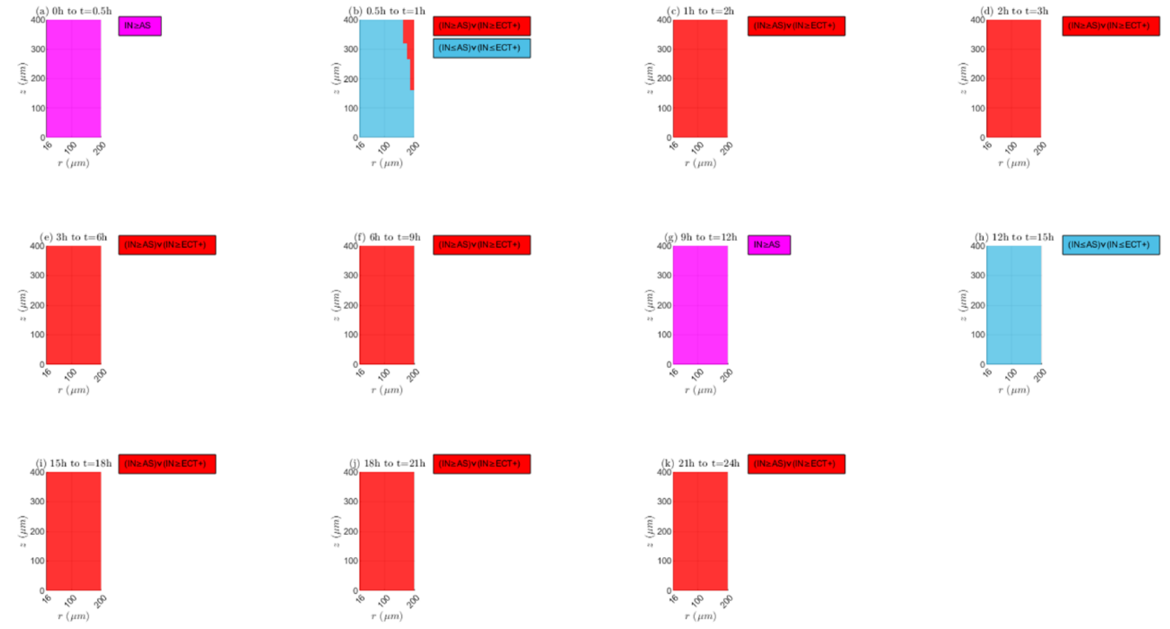

Changes with respect to Reference Case:
There is no changes

Sensitivity to ε for $E = 70kV/m$, UPK and $\lambda_{inl} = 0.001m/s$.

Reference Case **($\varepsilon = 0$)**

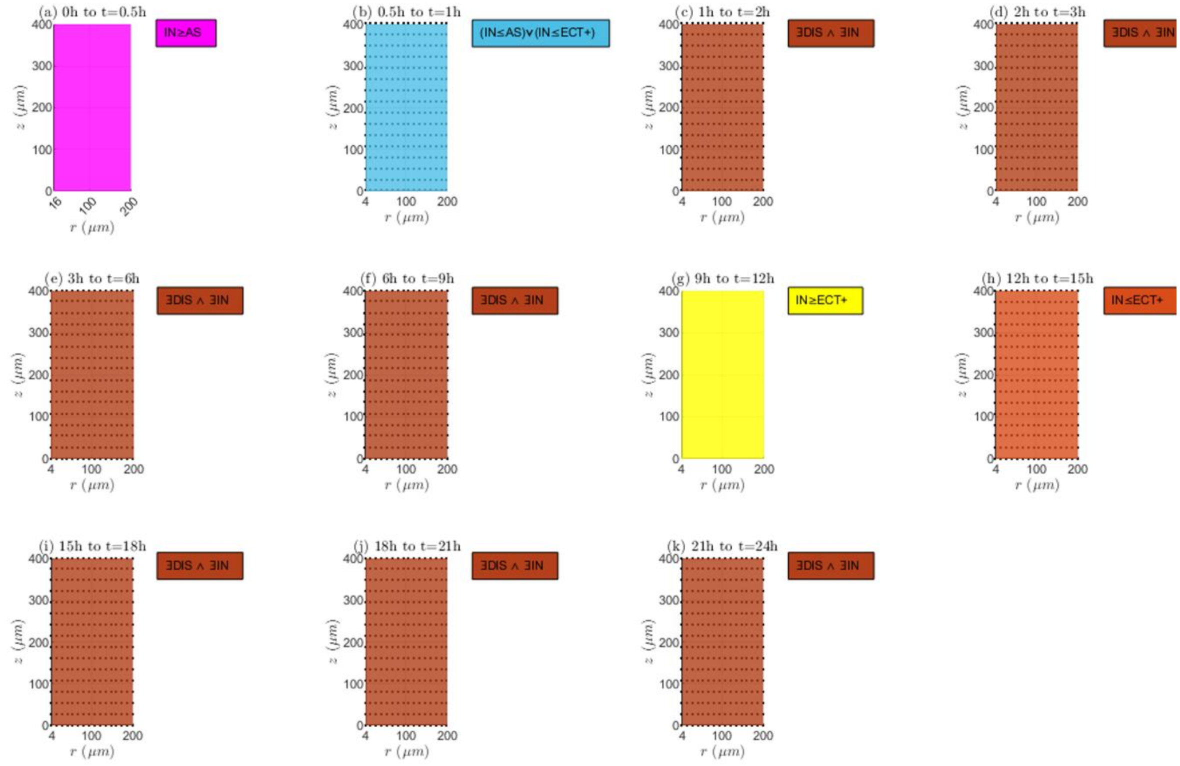

$\varepsilon = 1 \times 10^{-3}$

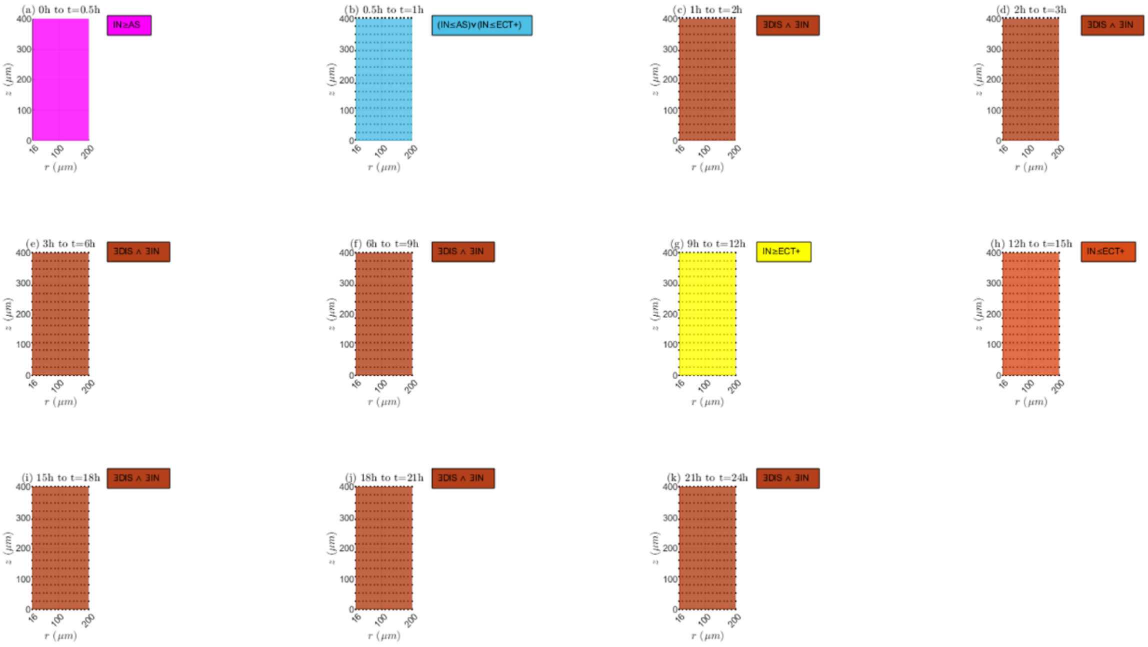

Changes with respect to Reference Case:

Between 9h to 12h C_1 decreases

$\varepsilon = -1 \times 10^{-3}$

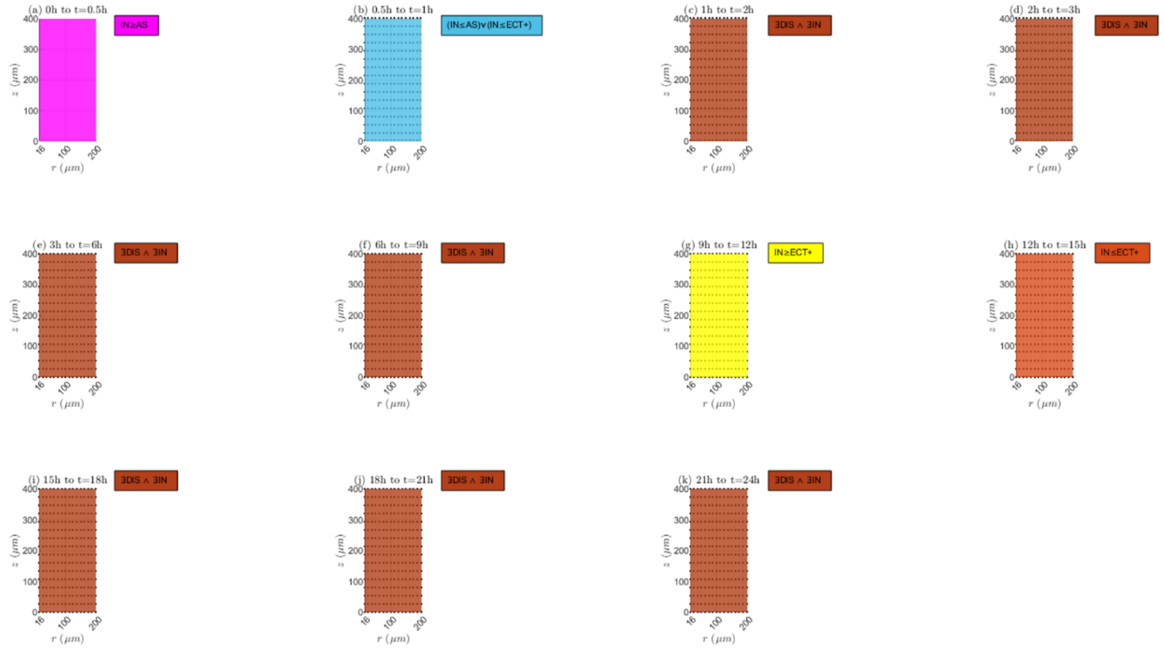

Changes with respect to Reference Case:
Between 9h to 12h C_1 decreases

Reference Case $\boldsymbol{\varepsilon = 1 \times 10^{-2}}$

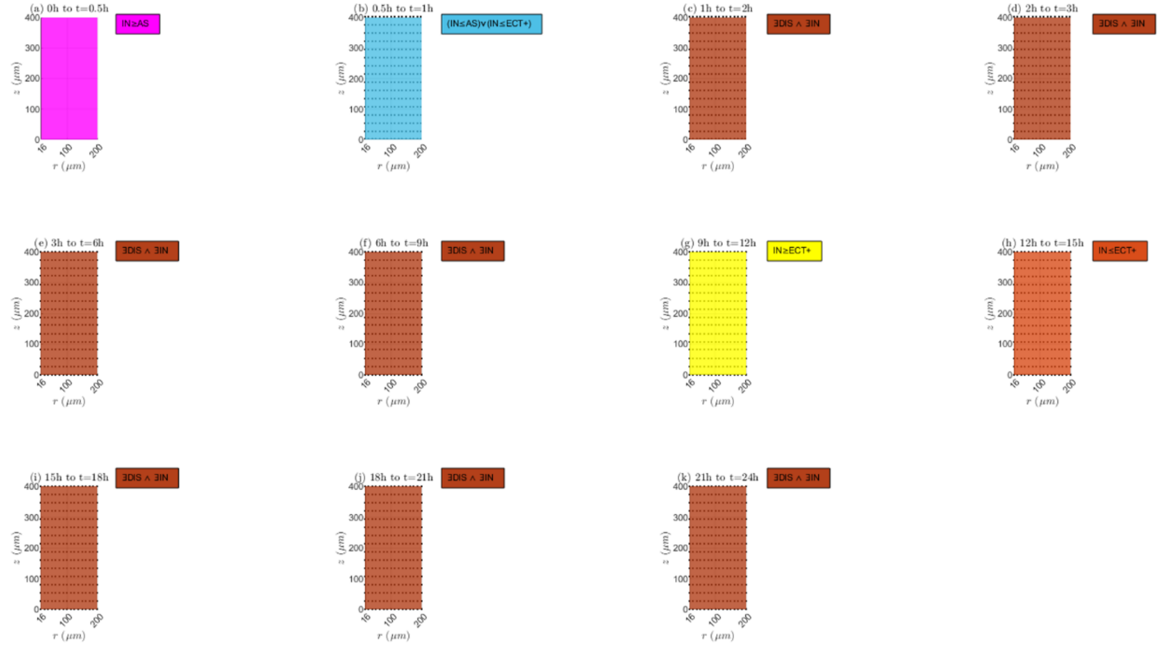

Changes with respect to Reference Case:
Between 9h to 12h C_1 decreases

$\boldsymbol{\varepsilon = -1 \times 10^{-2}}$

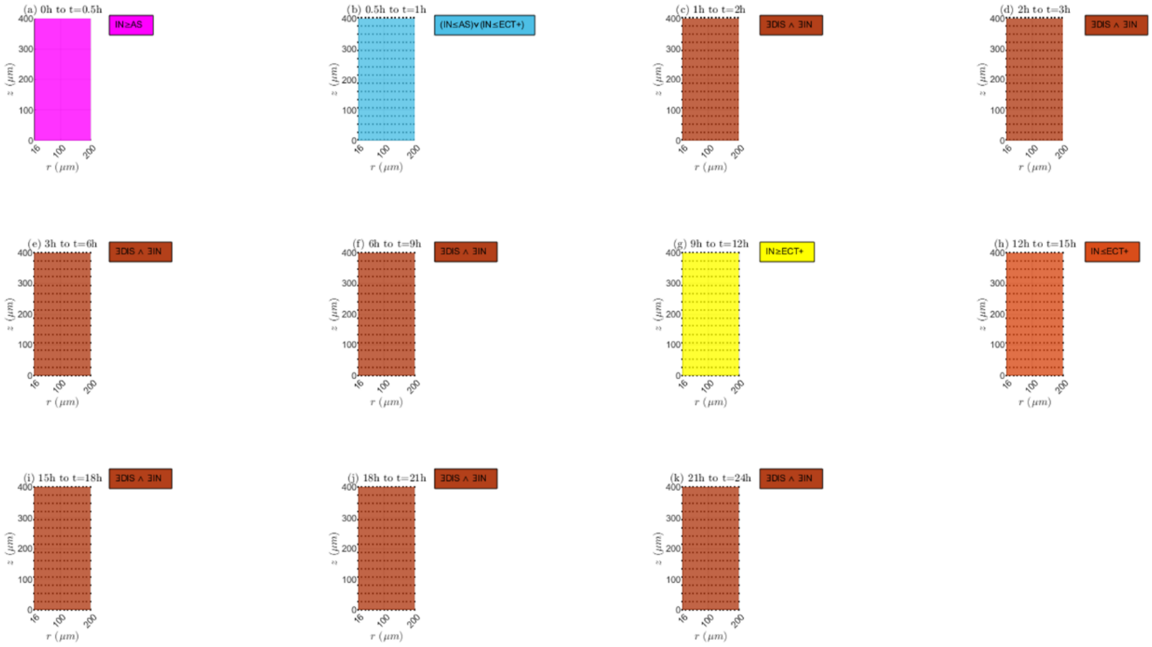

Changes with respect to Reference Case:
Between 9h to 12h C_1 decreases

$\boldsymbol{\varepsilon = 1 \times 10^{-1}}$

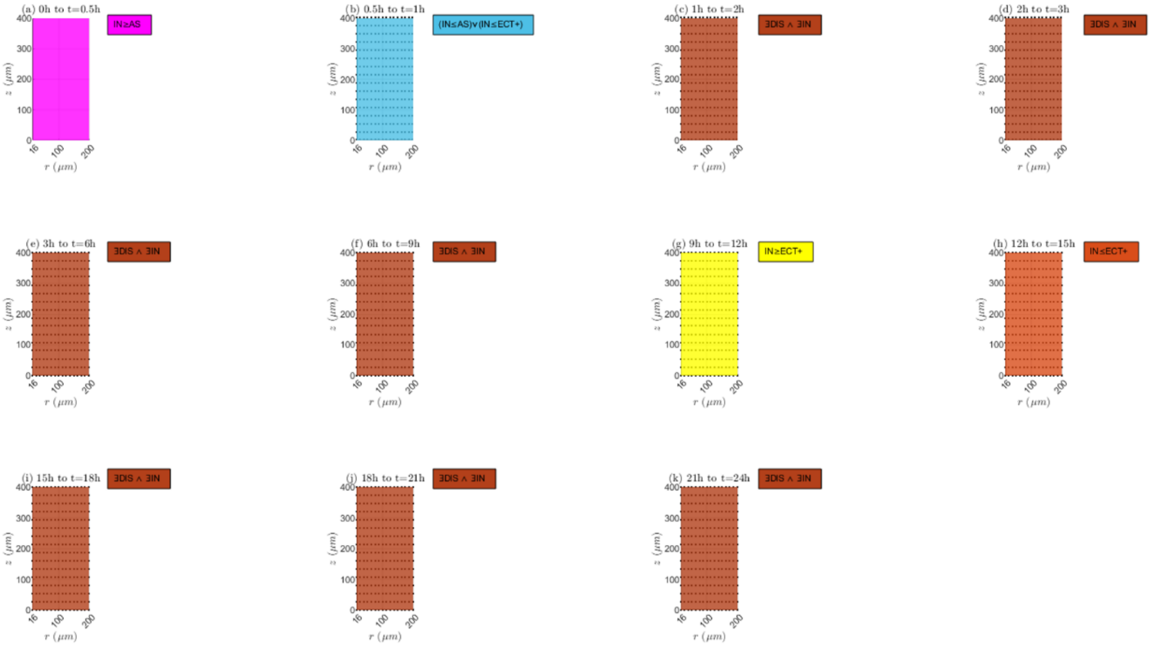

Changes with respect to Reference Case:
Between 9h to 12h C_1 decreases

$\boldsymbol{\varepsilon = -1 \times 10^{-1}}$

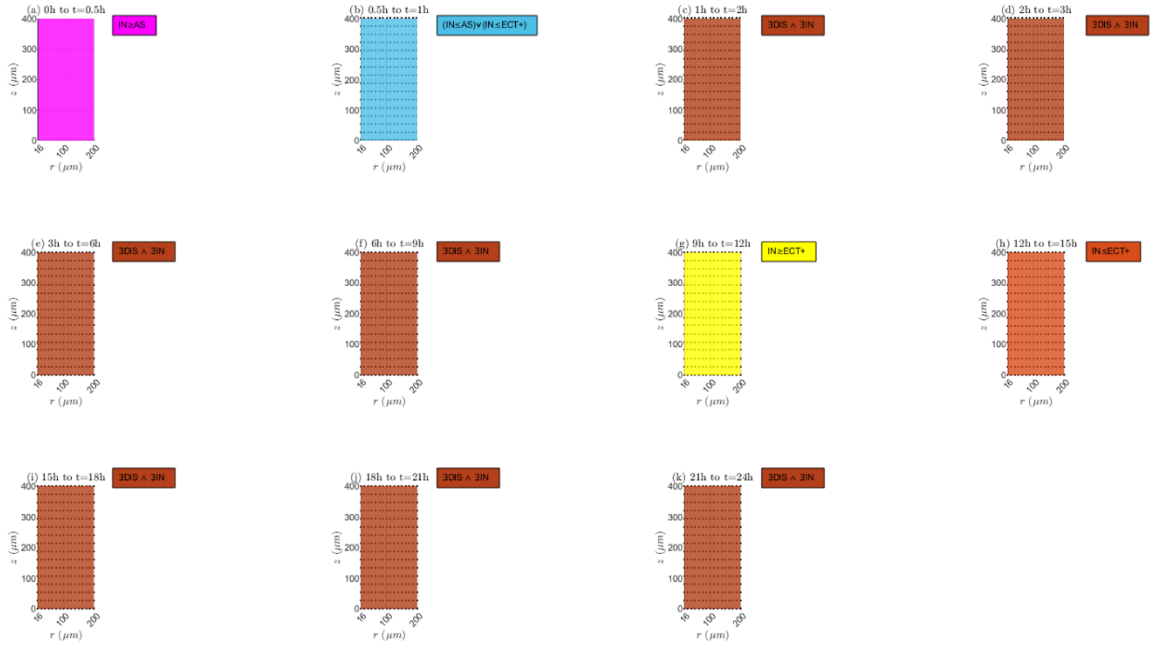

Changes with respect to Reference Case:
Between 9h to 12h C_1 decreases

Sensitivity to ε for $E = 70kV/m$, UPK and $\lambda_{inl} = 0.01m/s$.

Reference Case $(\boldsymbol{\varepsilon} = \mathbf{0})$

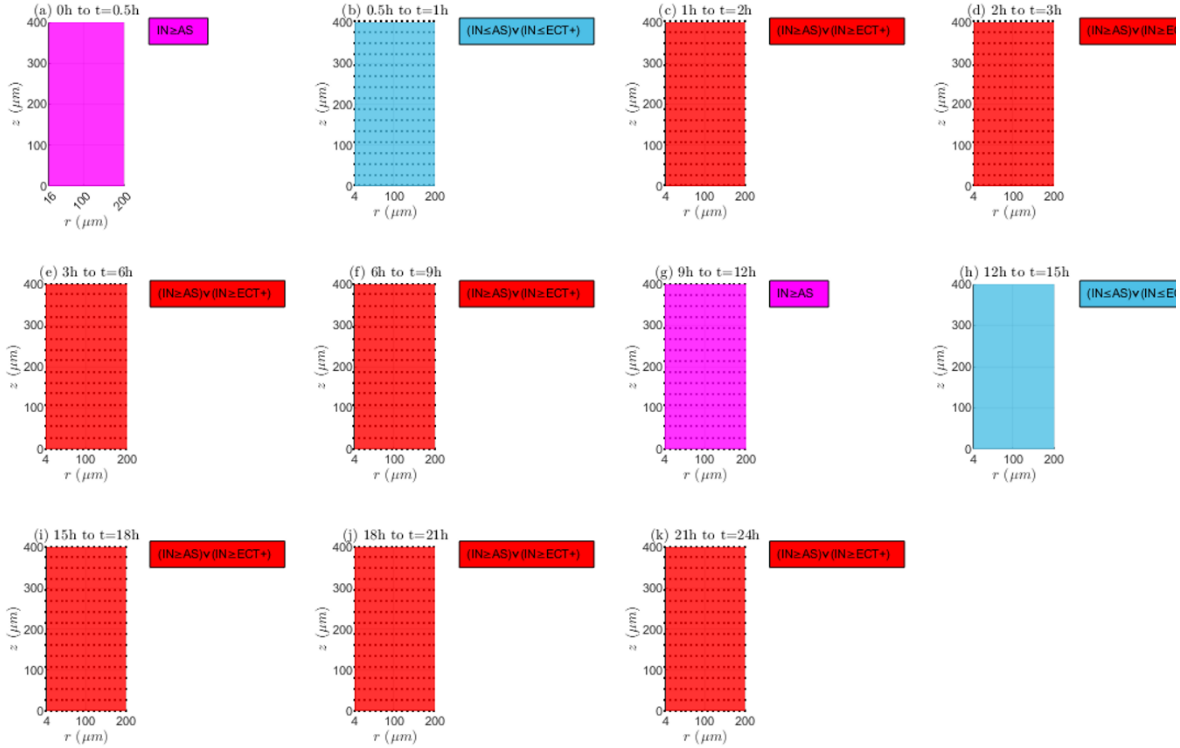

$\boldsymbol{\varepsilon = 1 \times 10^{-3}}$

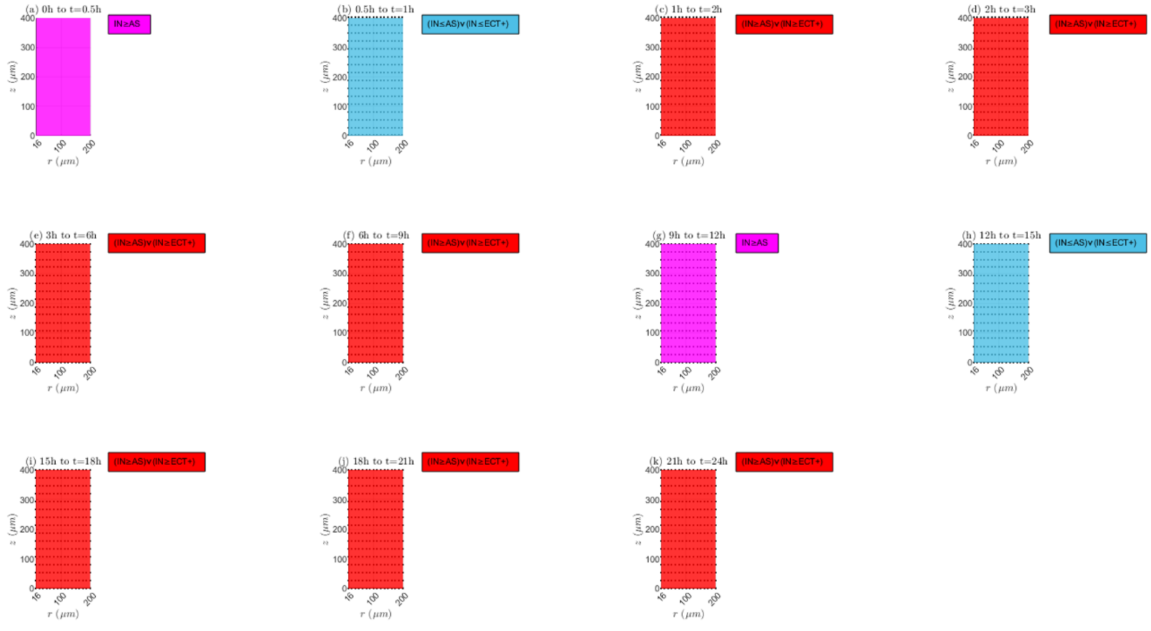

Changes with respect to Reference Case:

Between 12h to 15h and C_1 decreases

$\boldsymbol{\varepsilon = -1 \times 10^{-3}}$

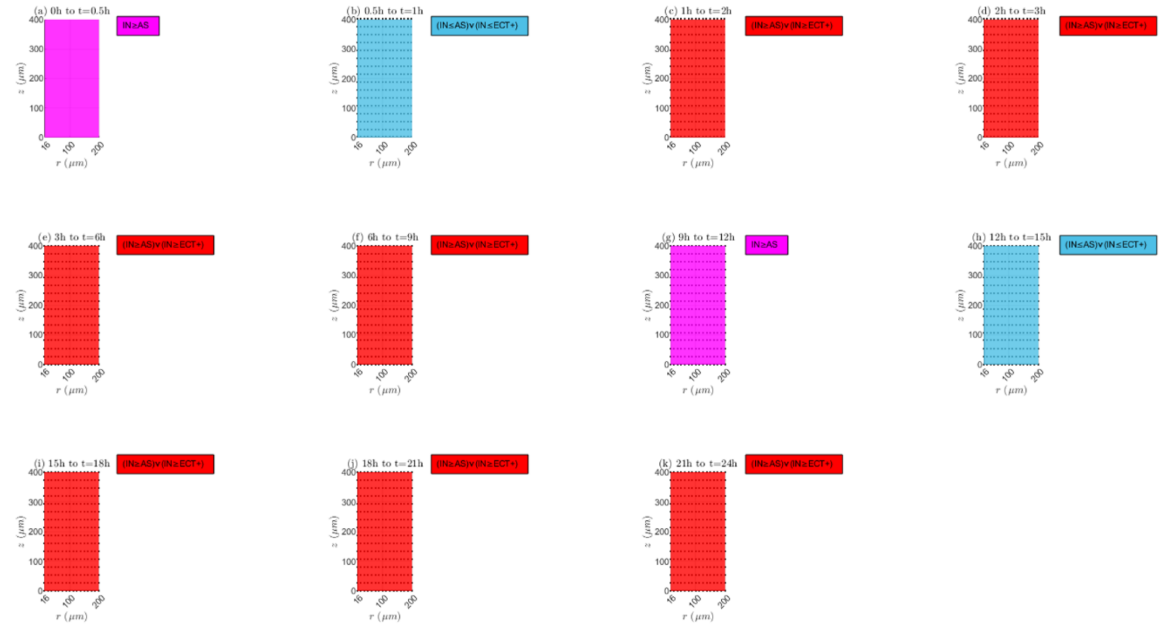

Changes with respect to Reference Case:
Between 12h to 15h C_1 decreases

$\boldsymbol{\varepsilon = 1 \times 10^{-2}}$

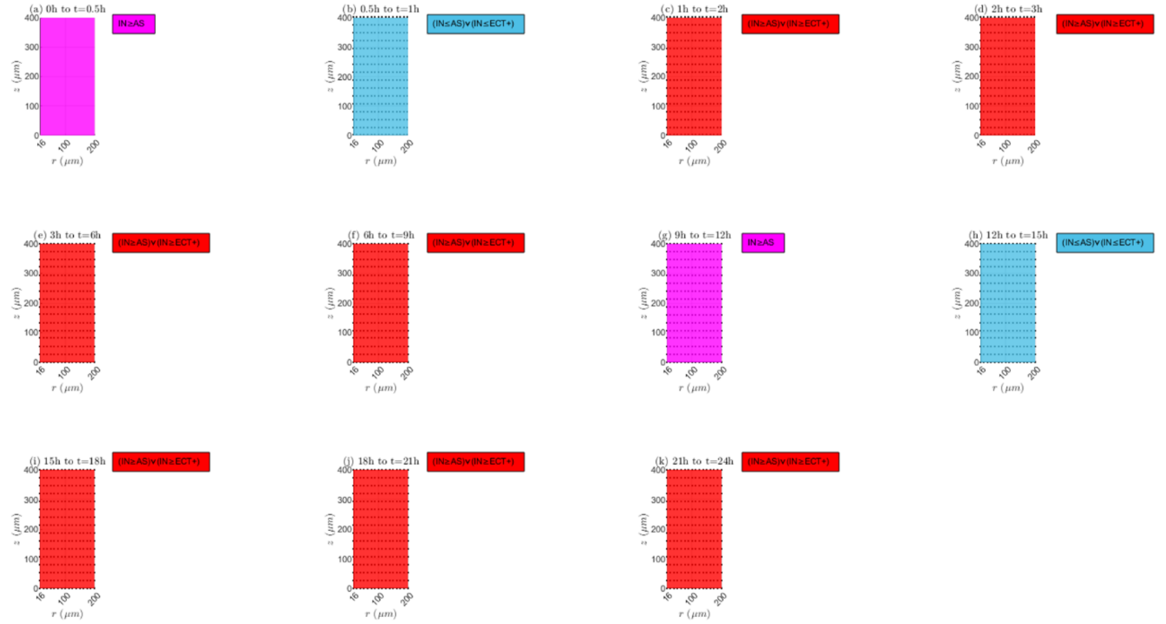

Changes with respect to Reference Case:
Between 12h to 15h C_1 decreases

$\boldsymbol{\varepsilon = -1 \times 10^{-2}}$

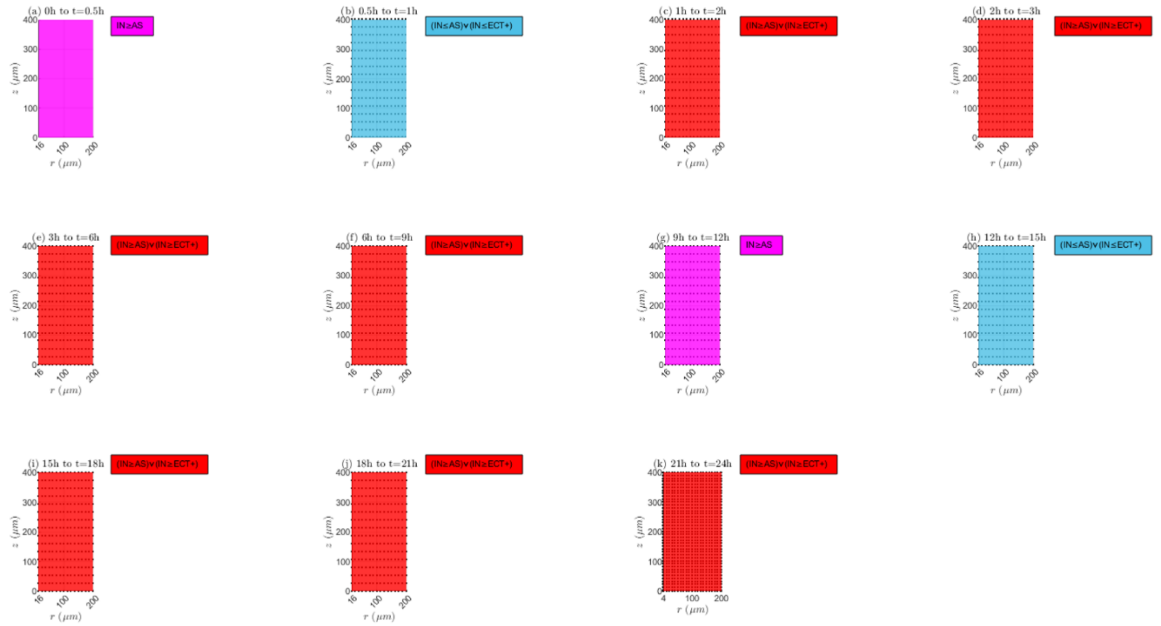

Changes with respect to Reference Case:
Between 12h to 15h C_1 decreases

$\boldsymbol{\varepsilon = 1 \times 10^{-1}}$

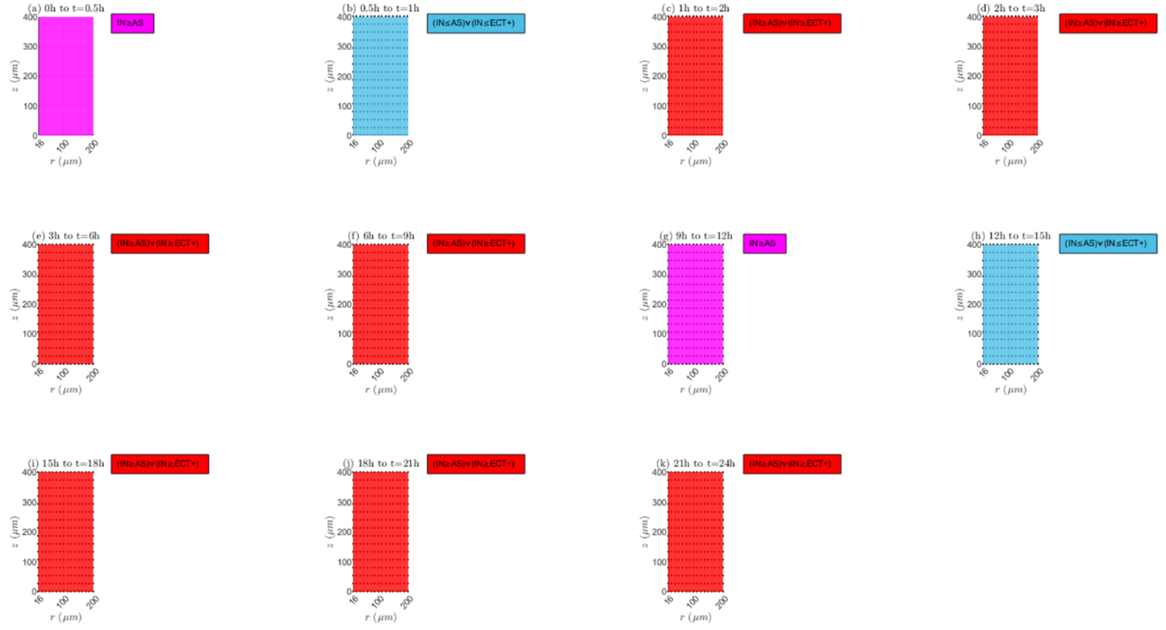

Changes with respect to Reference Case:
Between 12h to 15h C_1 decreases

$\boldsymbol{\varepsilon = -1 \times 10^{-1}}$

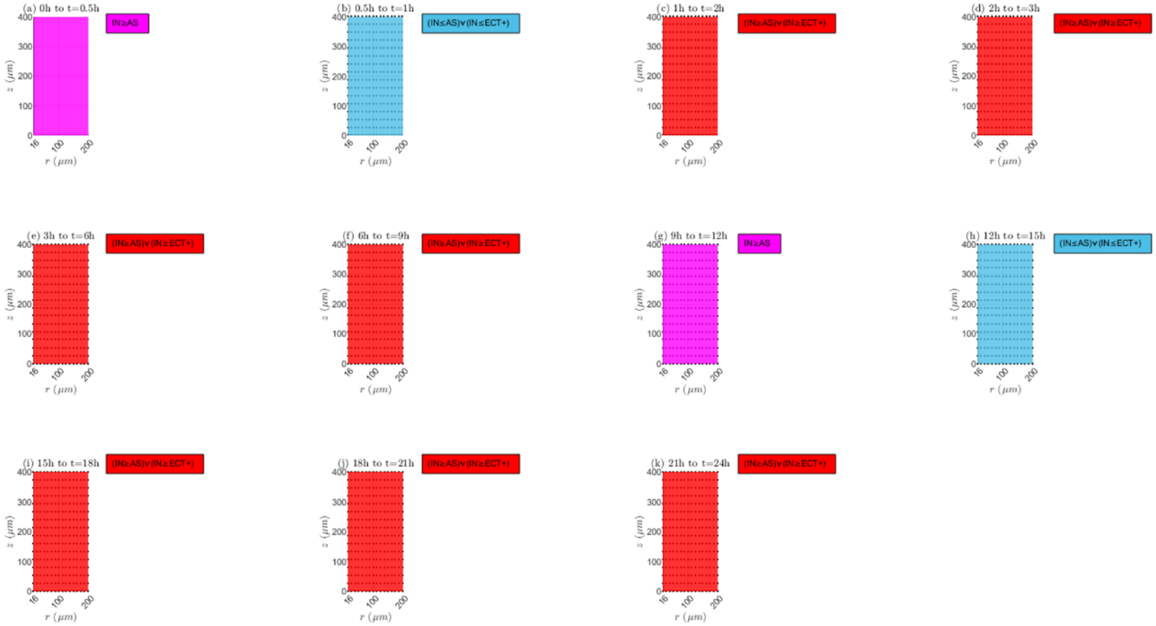

Changes with respect to Reference Case:

Between 12h to 15h C_1 decreases
