# Supplementary material for: Boolean network and meshless simulations for the comparison of transport and reaction mechanisms arising in one-short tri-exponential and uniform infusion electrochemotherapeutic treatments
Source: Front Bioinform. 2026 Mar 25;6:1719700. doi: 10.3389/fbinf.2026.1719700 (PMC13057505; doi:10.3389/fbinf.2026.1719700)
Supplement: Supplementary file 2 [file DataSheet1.pdf]

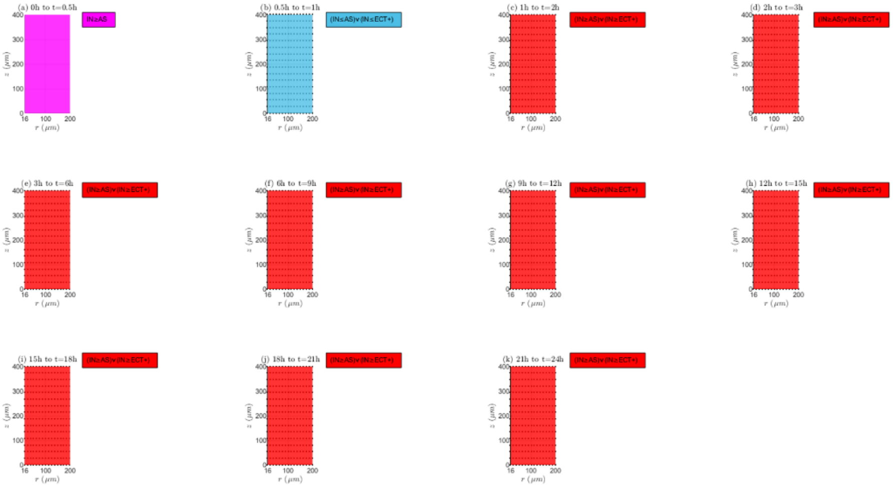

Figure 1. Reaction and transport mechanisms - $E = 0kV/m$, $\lambda_{inl} = 0.01m/s$ and TPK.

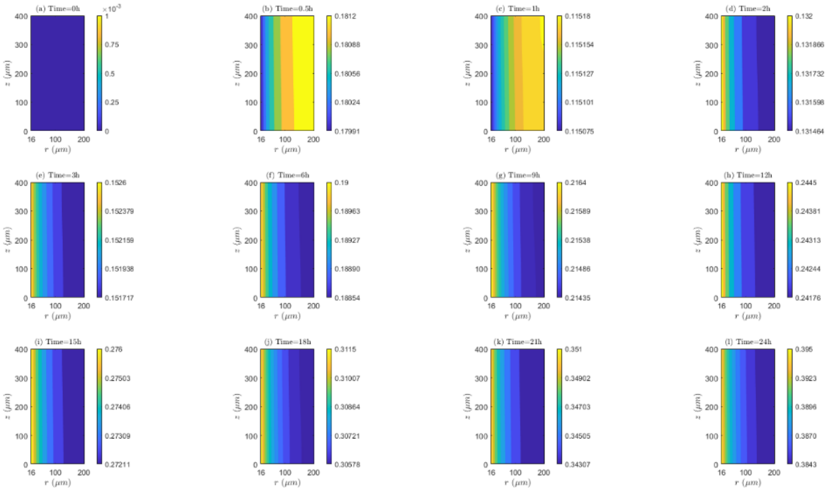

Figure 2. Contour plots of C_2/C_1 ratio - $E = 0kV/m$, $\lambda_{inl} = 0.001m/s$ and TPK.

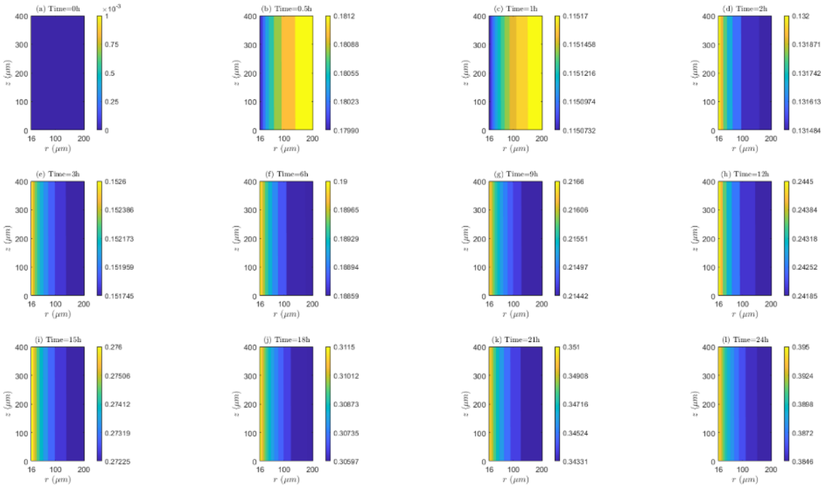

Figure 3. Contour plots of C_2/C_1 ratio - $E = 0kV/m$, $\lambda_{inl} = 0.01m/s$ and TPK.

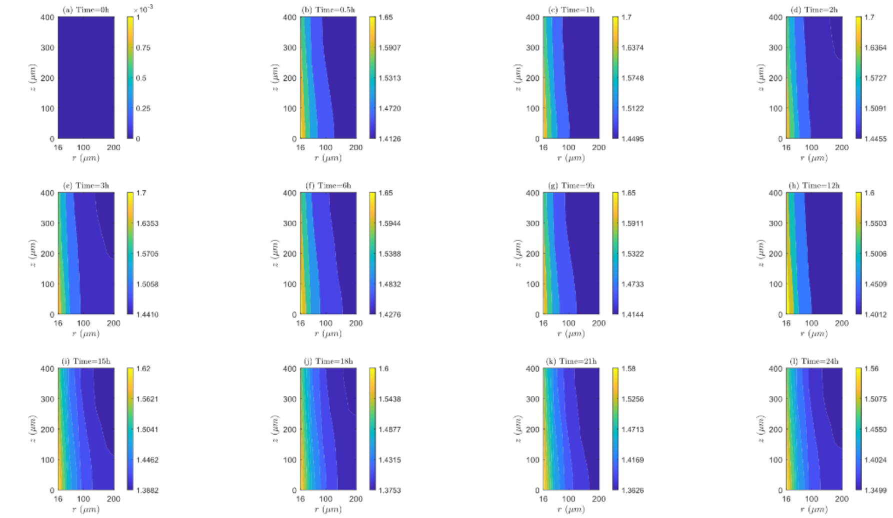

Figure 4. Bound intracellular concentration C_3 - $E = 0kV/m$, $\lambda_{inl} = 0.001m/s$ and TPK.

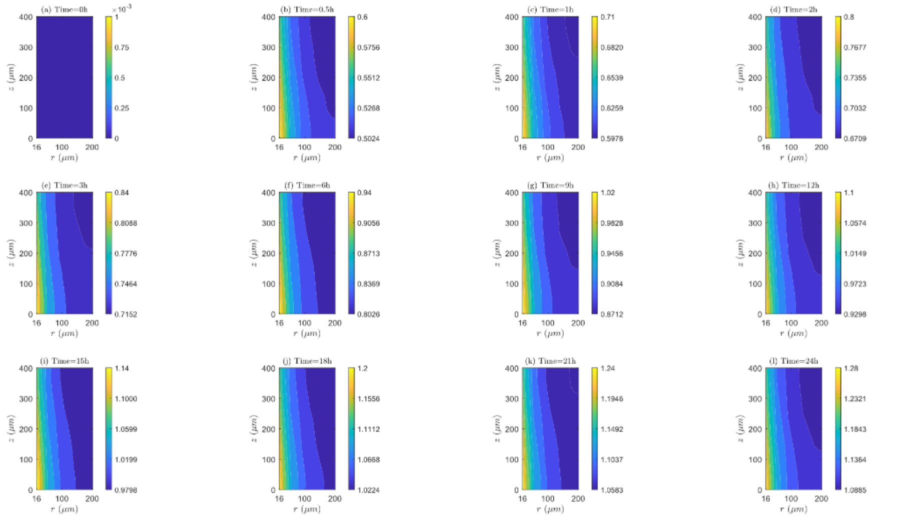

Figure 5. Bound intracellular concentration C_3 - $E = 0kV/m$, $\lambda_{inl} = 0.01m/s$ and TPK.

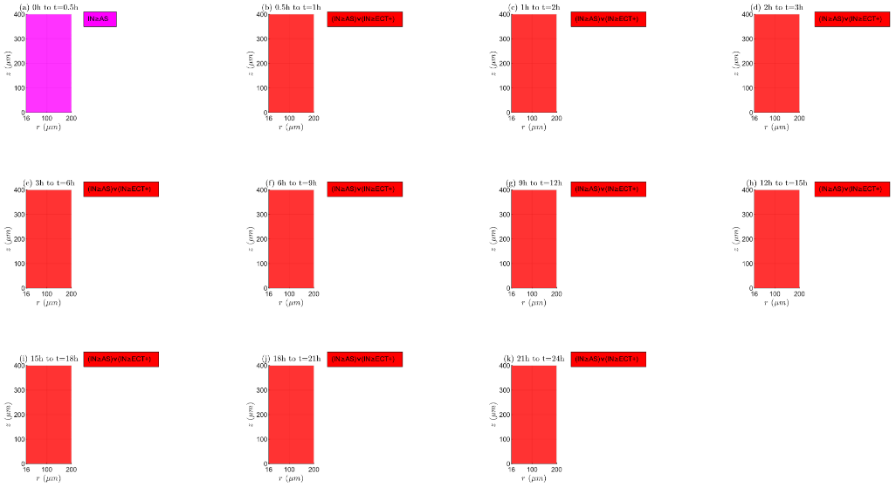

Figure 6. Reaction and transport mechanisms – $E = 0kV/m$, $\lambda_{inl} = 0.0001m/s$ and UPK.

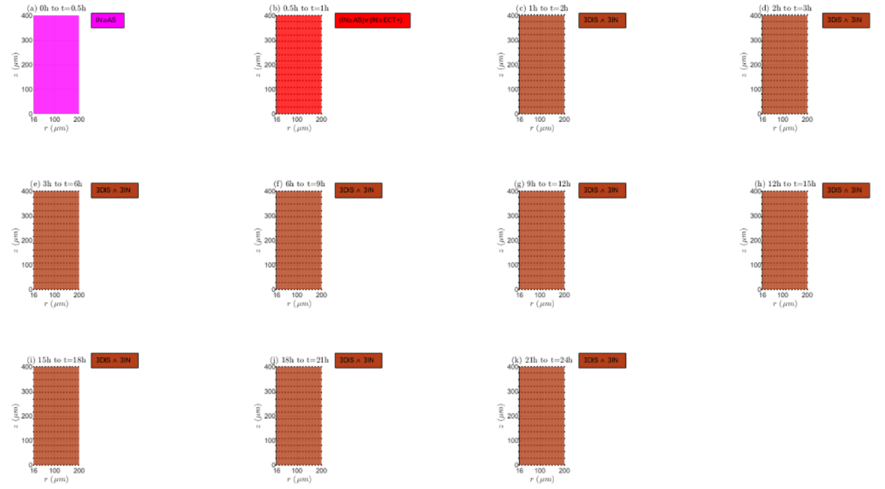

Figure 7. Reaction and transport mechanisms – $E = 0kV/m$, $\lambda_{inl} = 0.001m/s$ and UPK.

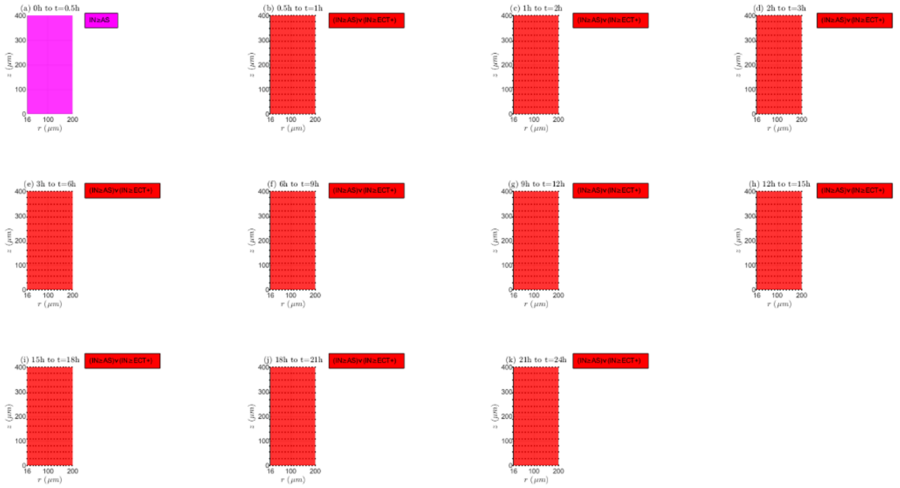

Figure 8. Reaction and transport mechanisms – $E = 0kV/m$, $\lambda_{inl} = 0.01m/s$ and UPK.

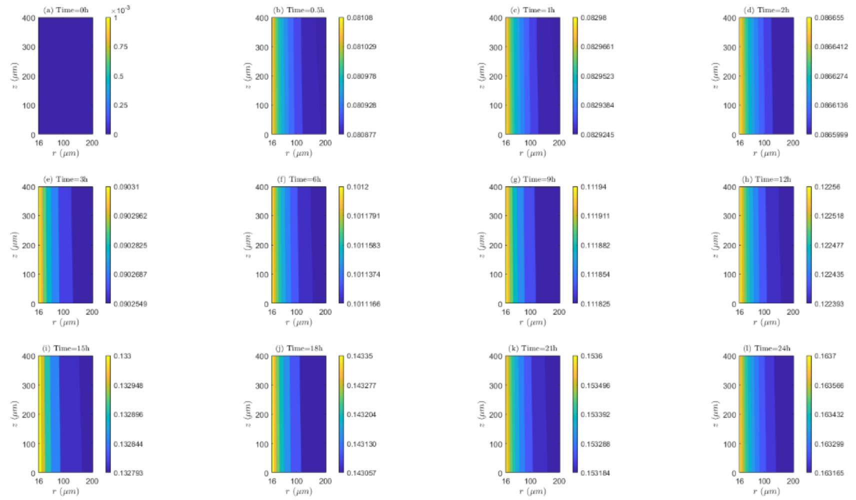

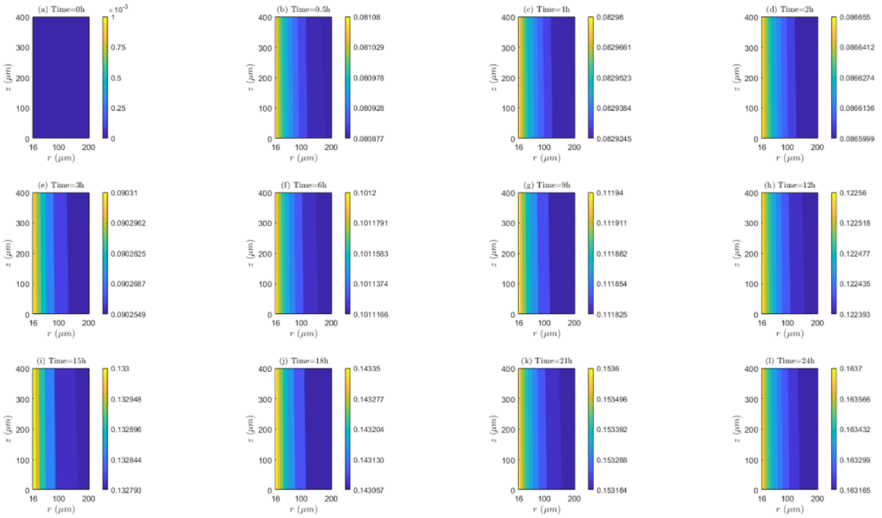

Figure 9. Contour plots of C_2/C_1 values - $E = 0kV/m$, $\lambda_{inl} = 0.001m/s$ and UPK.

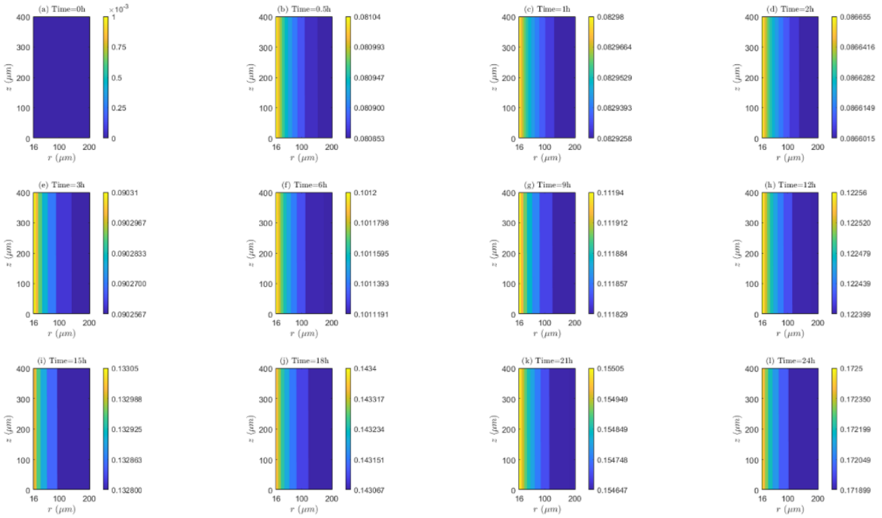

Figure 10. Contour plots of C_2/C_1 values - $E = 0kV/m$, $\lambda_{inl} = 0.01m/s$ and UPK.

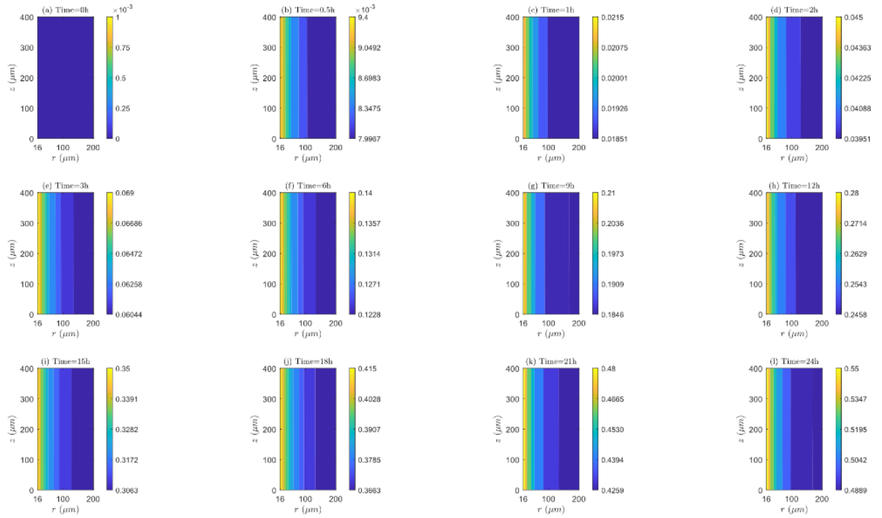

Figure 11. Bound intracellular concentration C_3 - $E = 0kV/m$, $\lambda_{inl} = 0.0001m/s$ and UPK.

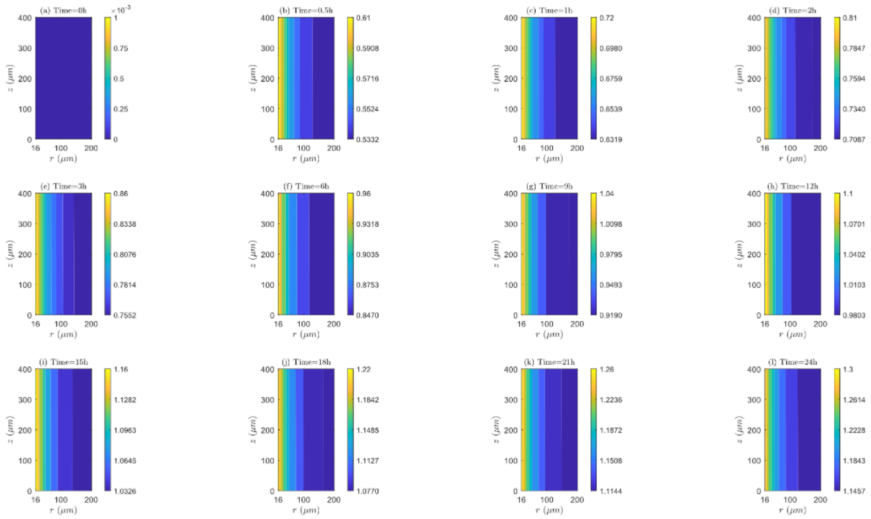

Figure 12. Bound intracellular concentration C_3 - $E = 0kV/m$, $\lambda_{inl} = 0.01m/s$ and UPK.

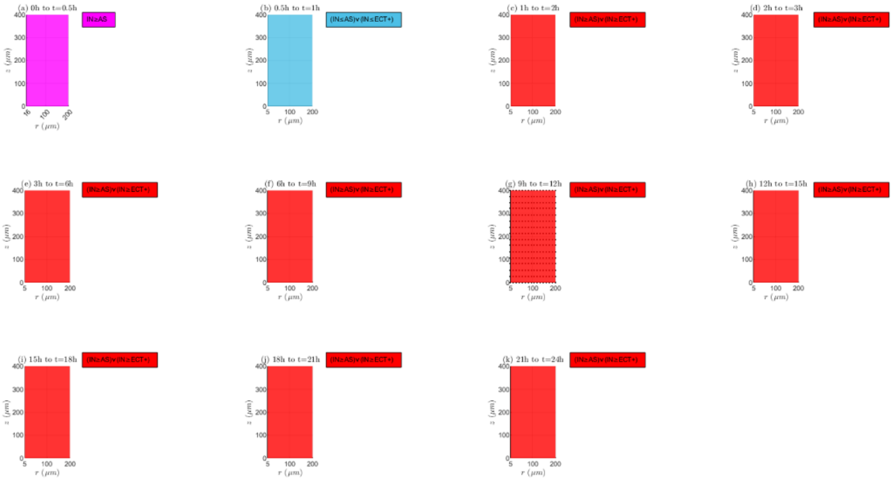

Figure 13. Reaction and transport mechanisms - $E = 46kV/m$, $\lambda_{inl} = 0.0001m/s$ and TPK.

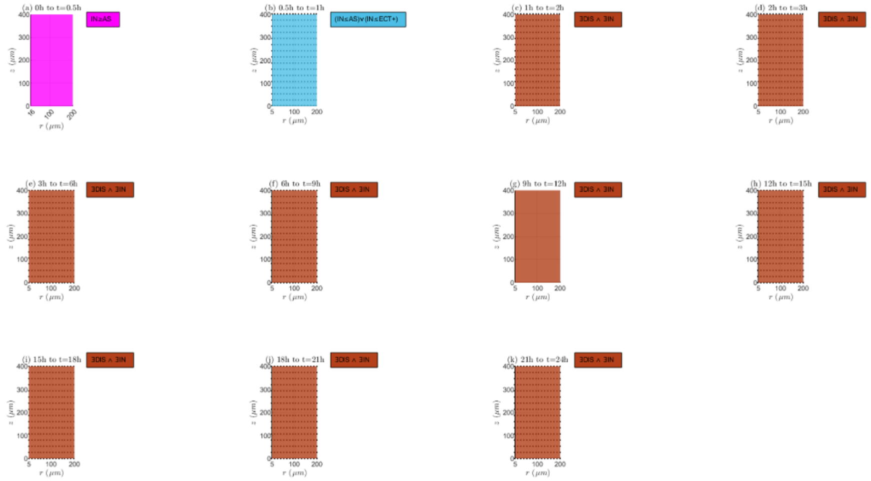

Figure 14. Reaction and transport mechanisms - $E = 46kV/m$, $\lambda_{inl} = 0.001m/s$ and TPK.

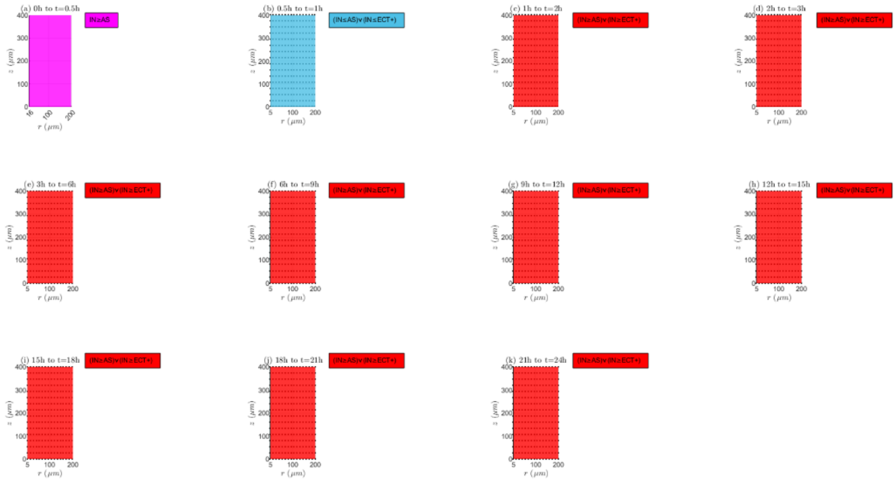

Figure 15. Reaction and transport mechanisms - $E = 46kV/m$, $\lambda_{inl} = 0.01m/s$ and TPK.

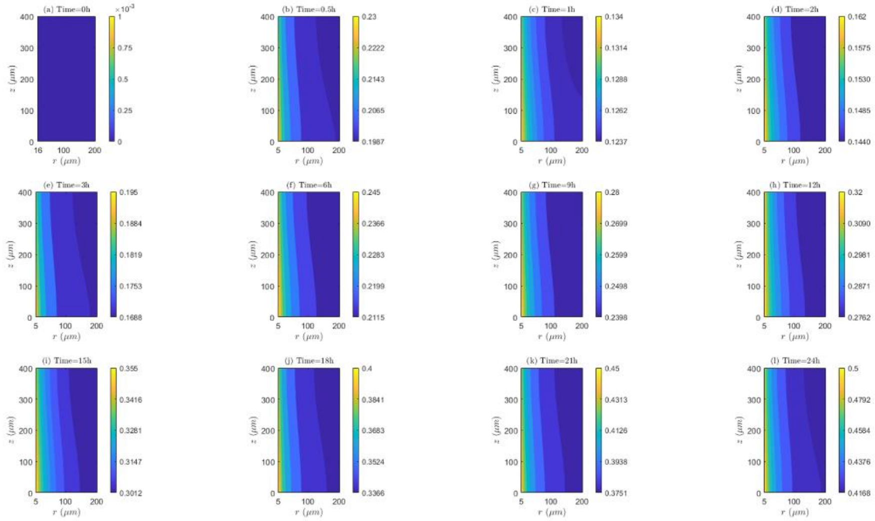

Figure 16. Contour plots of C_2/C_1 values - $E = 46kV/m$, $\lambda_{inl} = 0.001m/s$ and TPK.

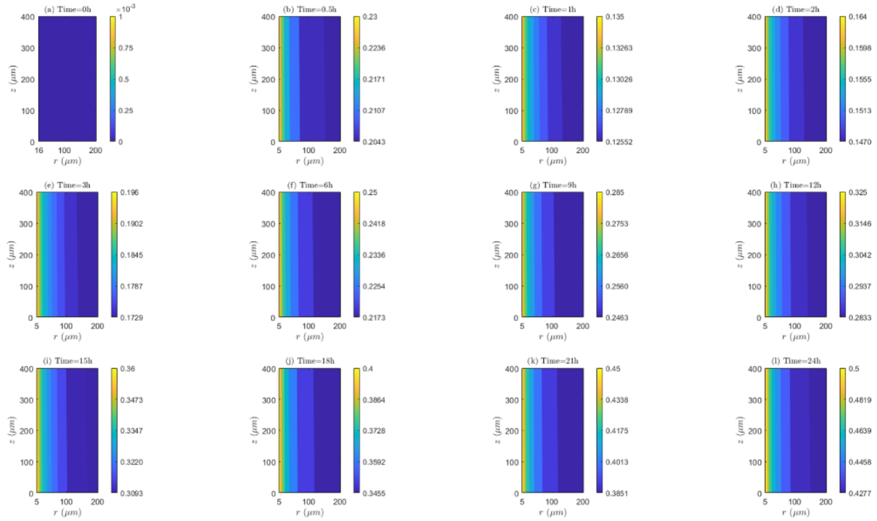

Figure 17. Contour plots of C_2/C_1 values - $E = 46kV/m$, $\lambda_{inl} = 0.01m/s$ and TPK.

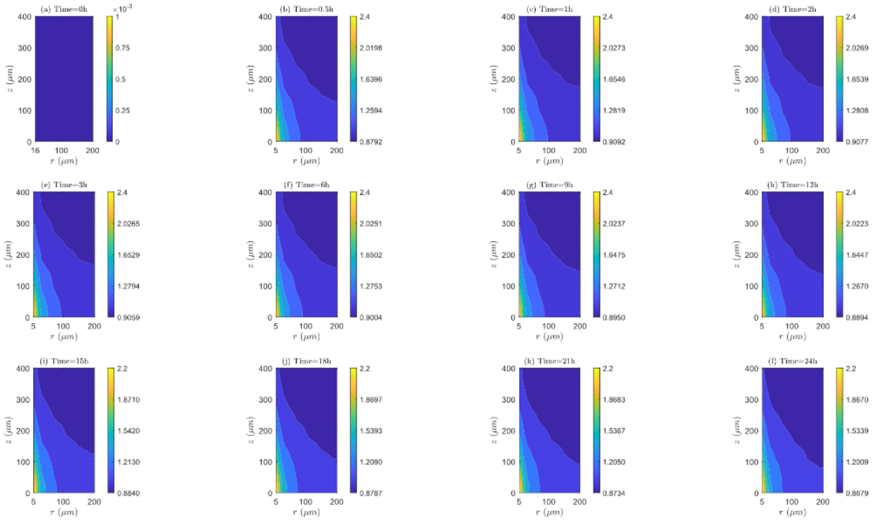

Figure 18. Bound intracellular concentration C_3 - $E = 46kV/m$, $\lambda_{inl} = 0.001m/s$ and TPK.

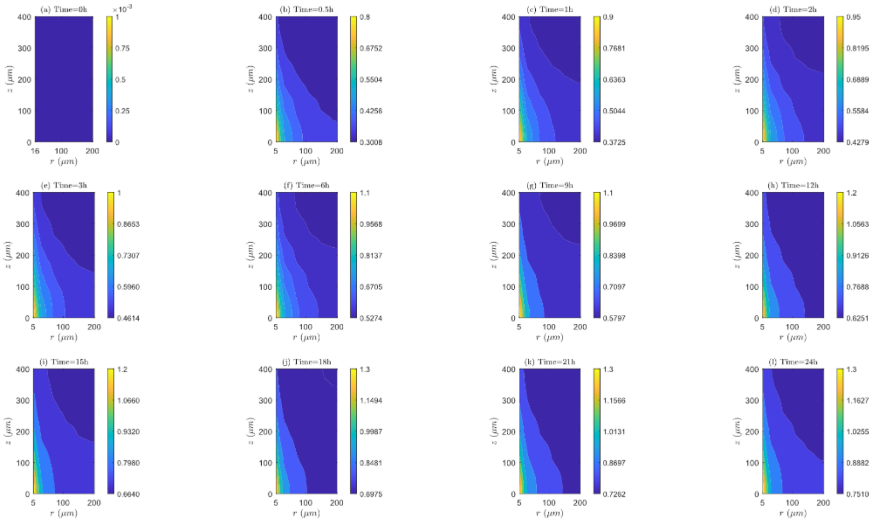

Figure 19. Bound intracellular concentration C_3 - $E = 46kV/m$, $\lambda_{inl} = 0.01m/s$ and TPK.

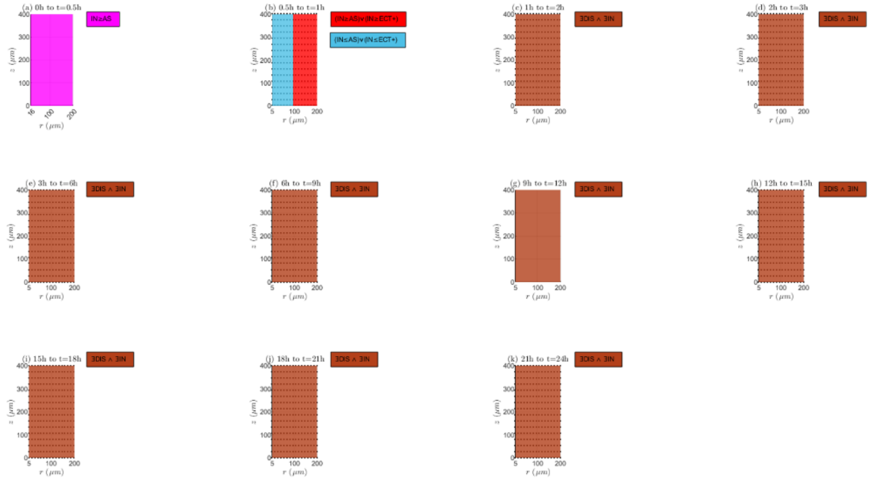

Figure 20. Reaction and transport mechanisms - $E = 46kV/m$, $\lambda_{inl} = 0.001m/s$ and UPK.

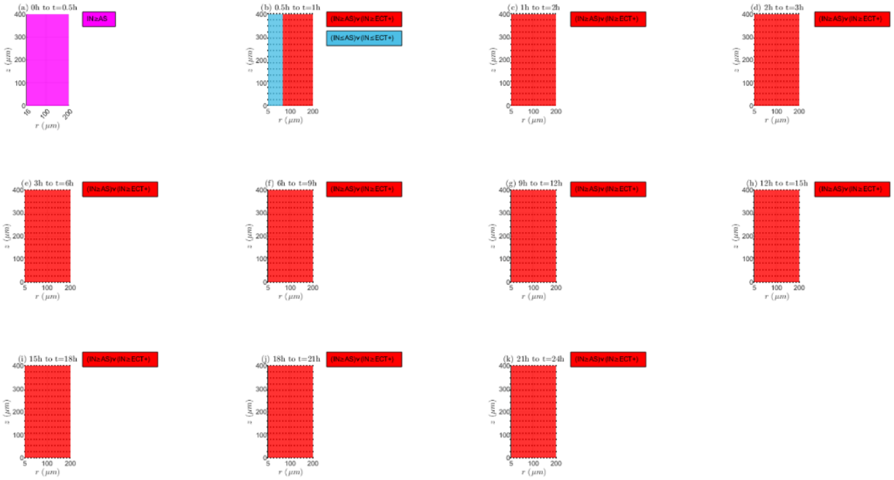

Figure 21. Reaction and transport mechanisms - $E = 46kV/m$, $\lambda_{inl} = 0.01m/s$ and UPK.

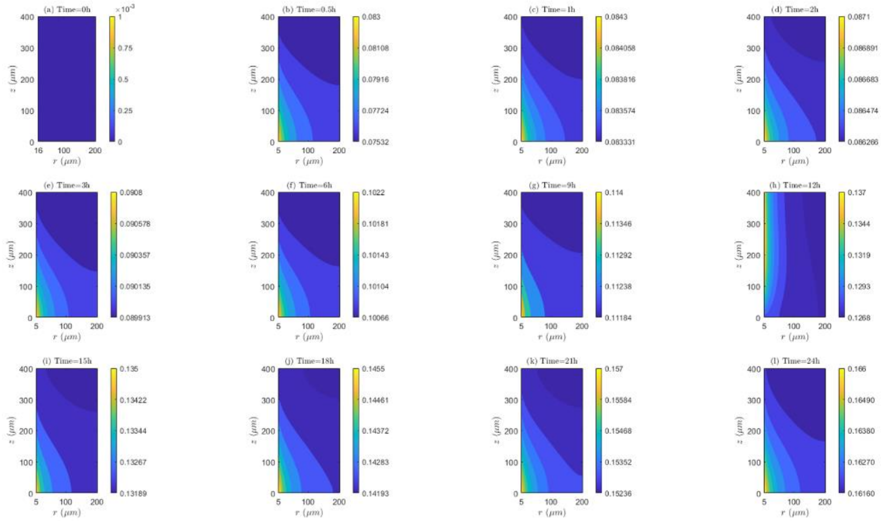

Figure 22. Contour plots of C_2/C_1 values - $E = 46kV/m$, $\lambda_{inl} = 0.0001m/s$ and UPK.

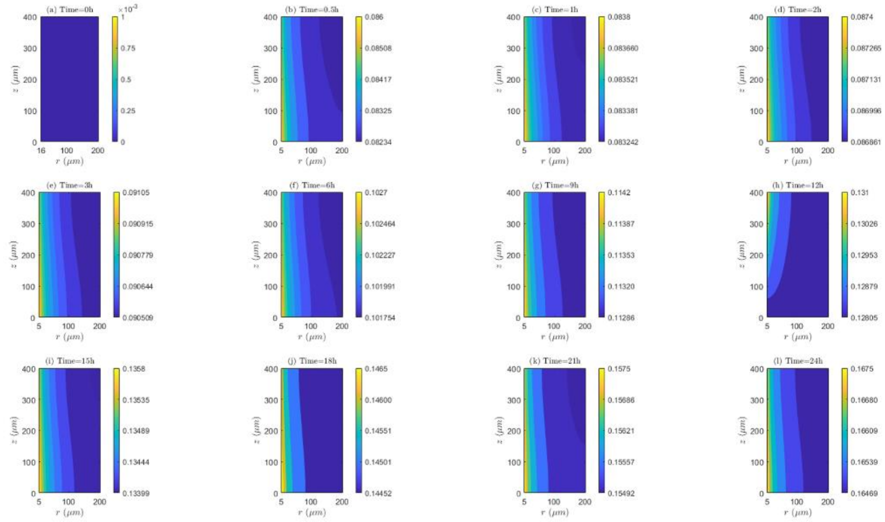

Figure 23. Contour plots of C_2/C_1 values - $E = 46kV/m$, $\lambda_{inl} = 0.001m/s$ and UPK.

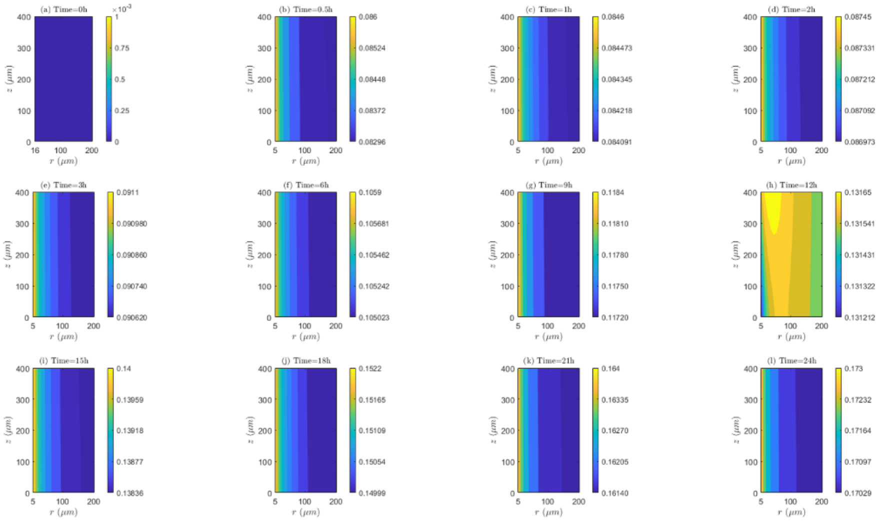

Figure 24. Contour plots of C_2/C_1 values - $E = 46kV/m$, $\lambda_{inl} = 0.01m/s$ and UPK.

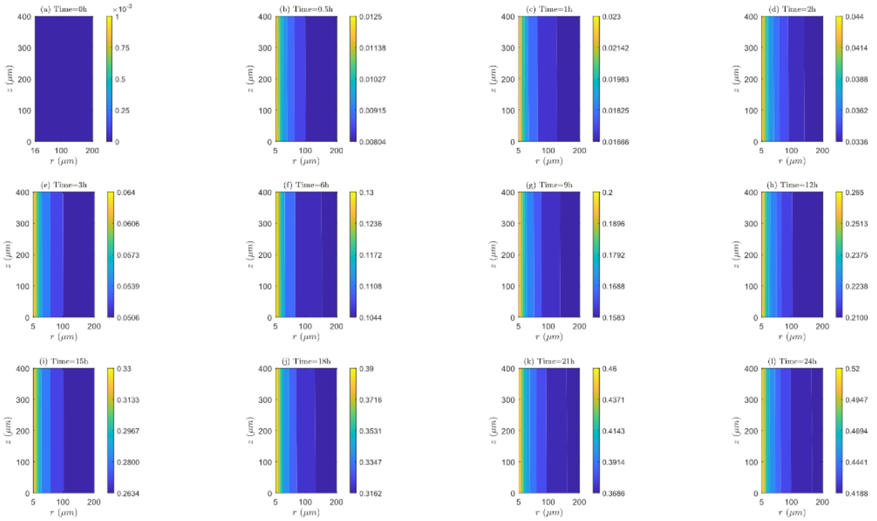

Figure 25. Bound intracellular concentration C_3 - $E = 46kV/m$, $\lambda_{inl} = 0.0001m/s$ and UPK.

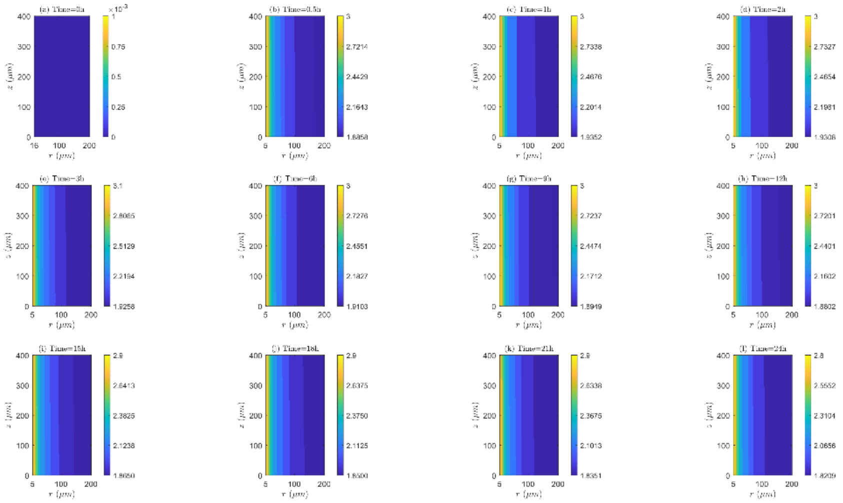

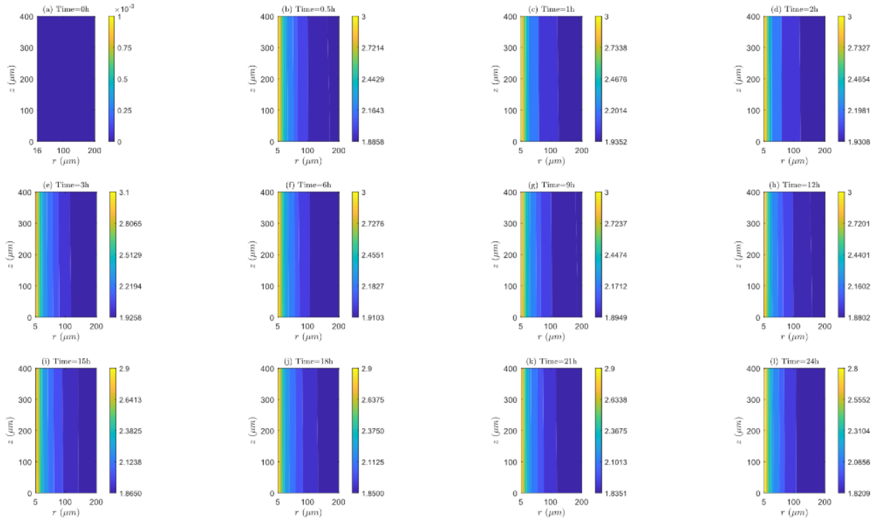

Figure 26. Bound intracellular concentration C_3 - $E = 46kV/m$, $\lambda_{inl} = 0.001m/s$ and UPK.

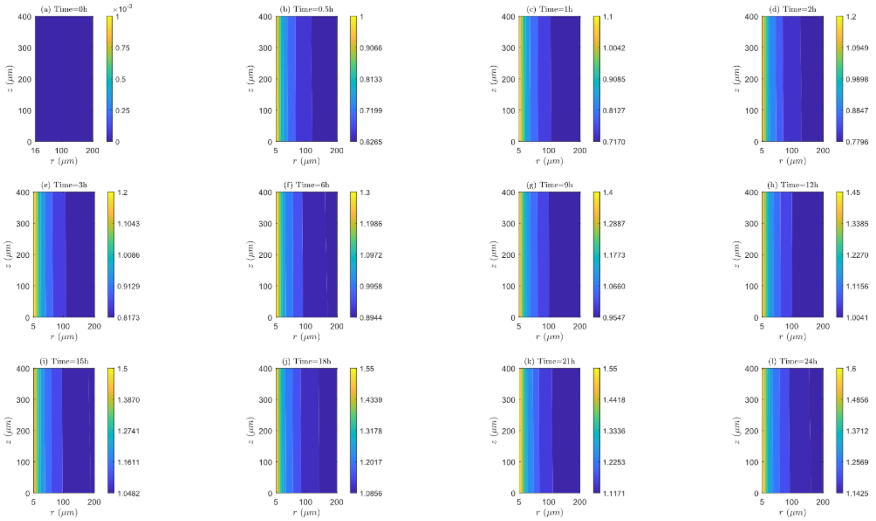

Figure 27. Bound intracellular concentration C_3 - $E = 46kV/m$, $\lambda_{inl} = 0.01m/s$ and UPK.

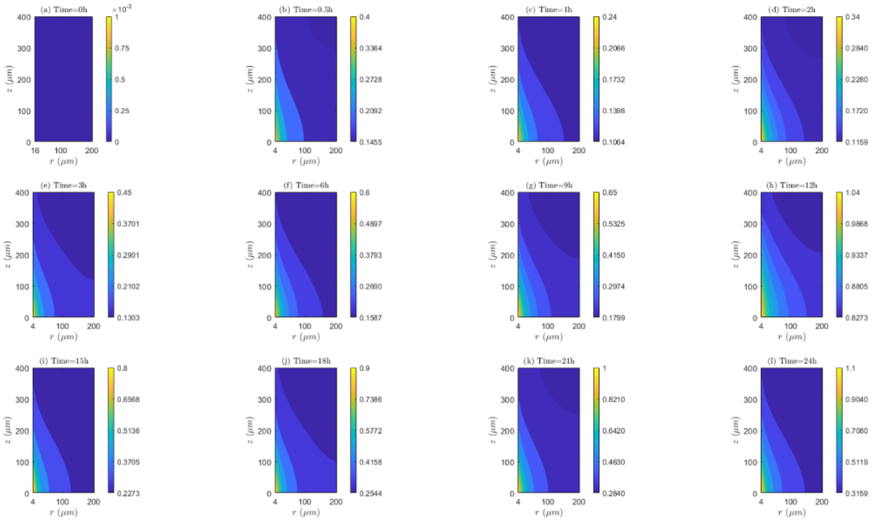

Figure 28. Contour plots of C_2/C_1 values - $E = 70kV/m$, $\lambda_{inl} = 0.0001m/s$ and TPK.

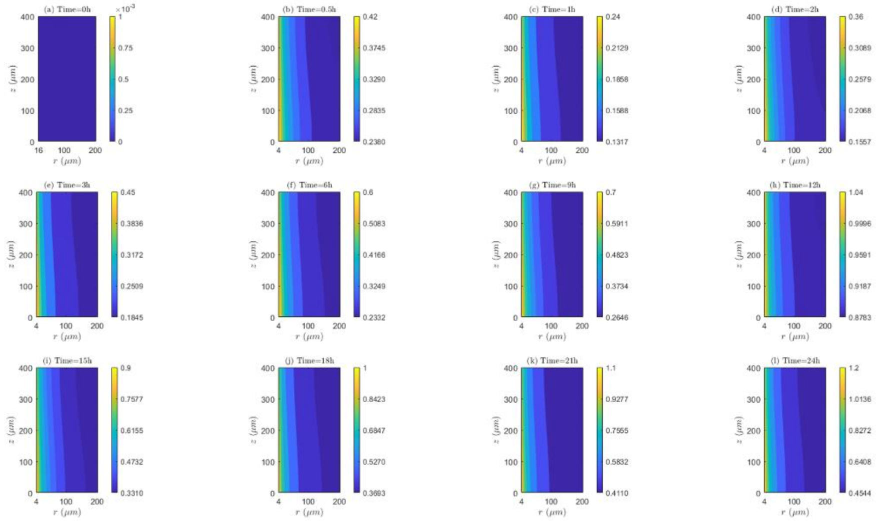

Figure 29. Contour plots of C_2/C_1 values - $E = 70kV/m$, $\lambda_{inl} = 0.001m/s$ and TPK.

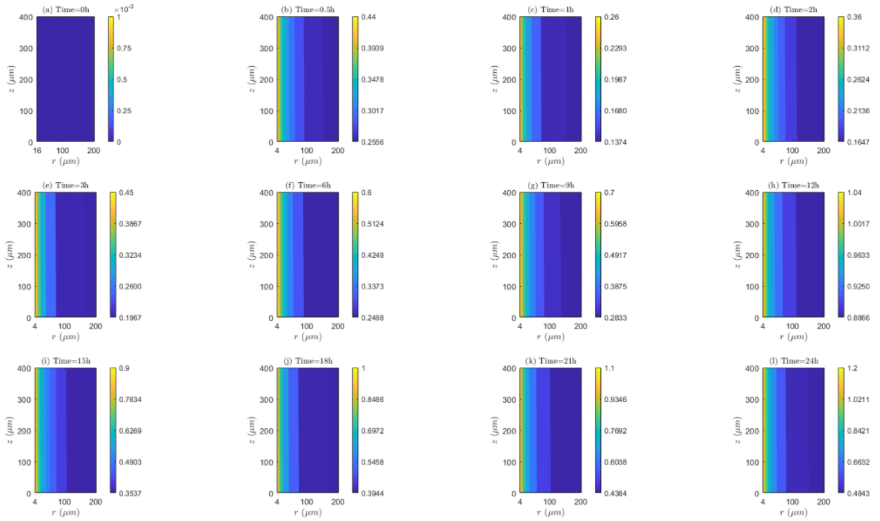

Figure 30. Contour plots of C_2/C_1 values - $E = 70kV/m$, $\lambda_{inl} = 0.01m/s$ and TPK.

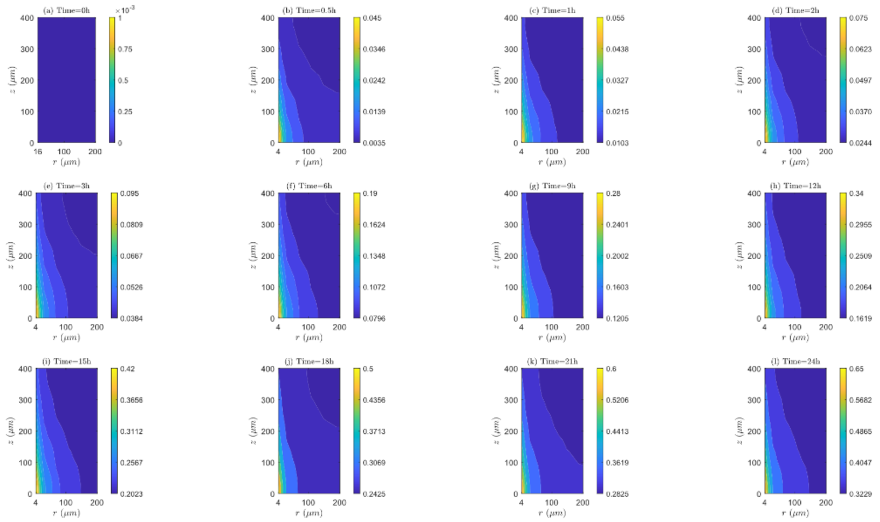

Figure 31. Bound intracellular concentration C_3 - $E = 70kV/m$, $\lambda_{inl} = 0.0001m/s$ and TPK.

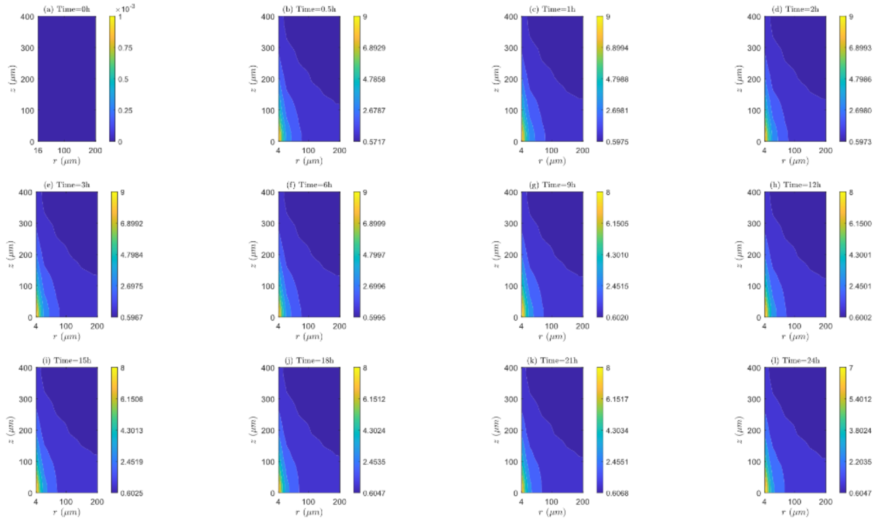

Figure 32. Bound intracellular concentration C_3 - $E = 70kV/m$, $\lambda_{inl} = 0.001m/s$ and TPK.

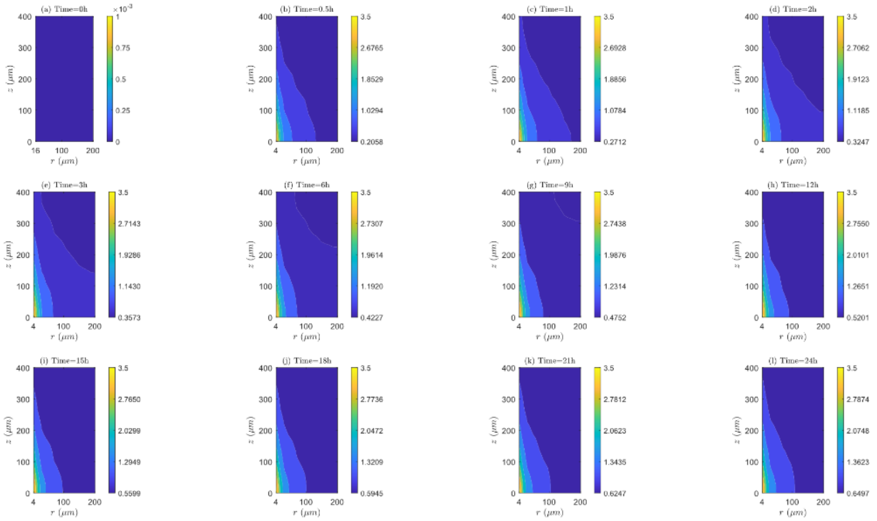

Figure 33. Bound intracellular concentration C_3 - $E = 70kV/m$, $\lambda_{inl} = 0.01m/s$ and TPK.

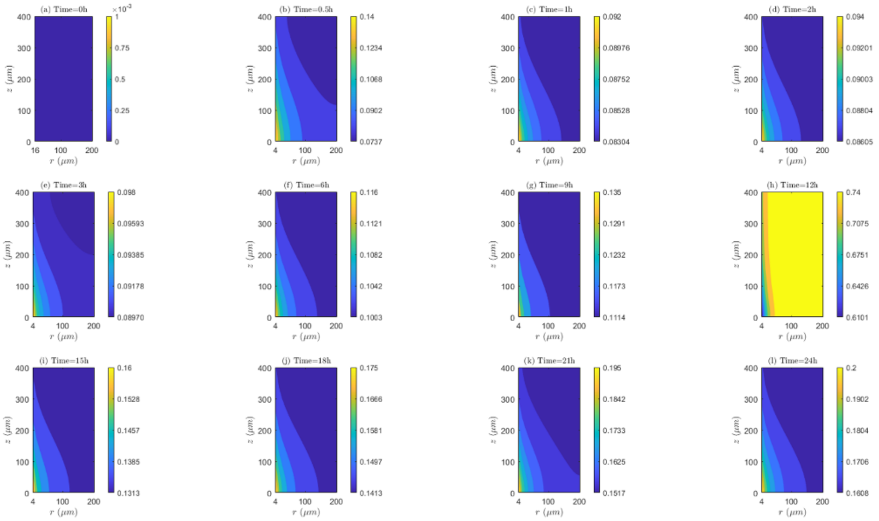

Figure 34. Contour plots of C_2/C_1 values - $E = 70kV/m$, $\lambda_{inl} = 0.0001m/s$ and UPK.

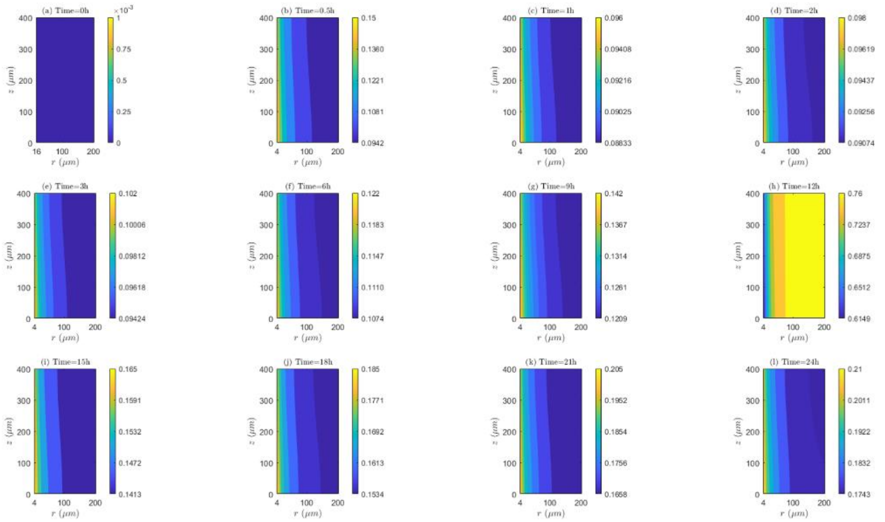

Figure 35. Contour plots of C_2/C_1 values - $E = 70kV/m$, $\lambda_{inl} = 0.001m/s$ and UPK.

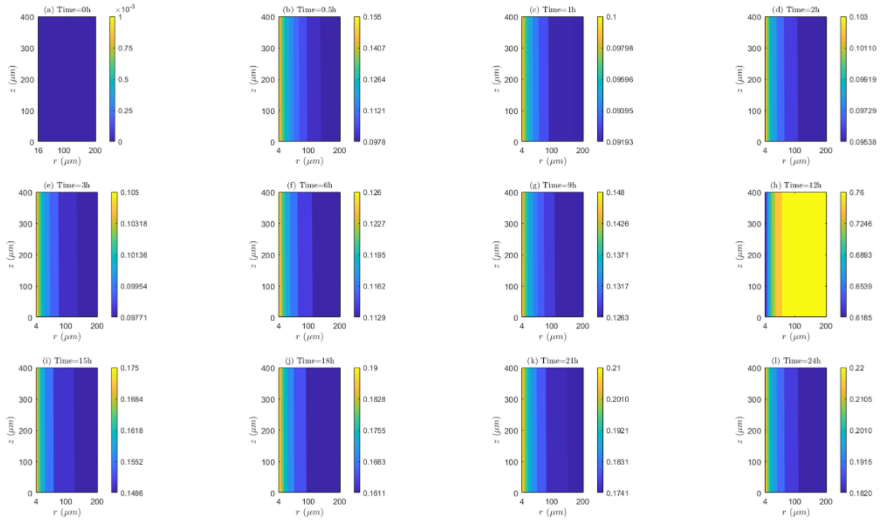

Figure 36. Contour plots of C_2/C_1 values - $E = 70kV/m$, $\lambda_{inl} = 0.01m/s$ and UPK.

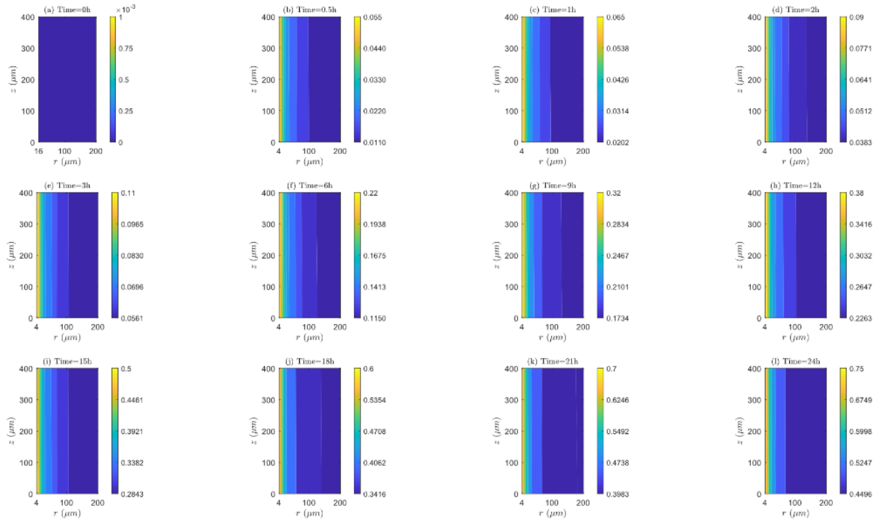

Figure 37. Bound intracellular concentration C_3 - $E = 70kV/m$, $\lambda_{inl} = 0.0001m/s$ and UPK.

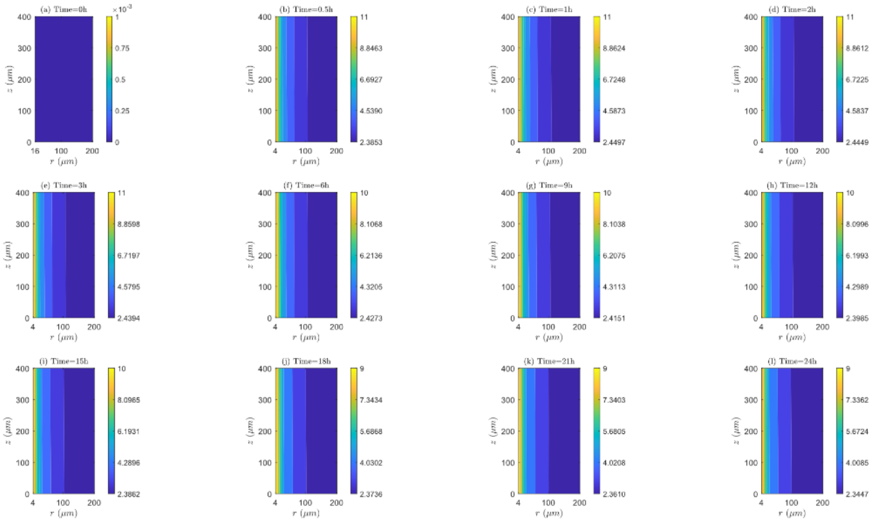

Figure 38. Bound intracellular concentration C_3 - $E = 70kV/m$, $\lambda_{inl} = 0.001m/s$ and UPK.

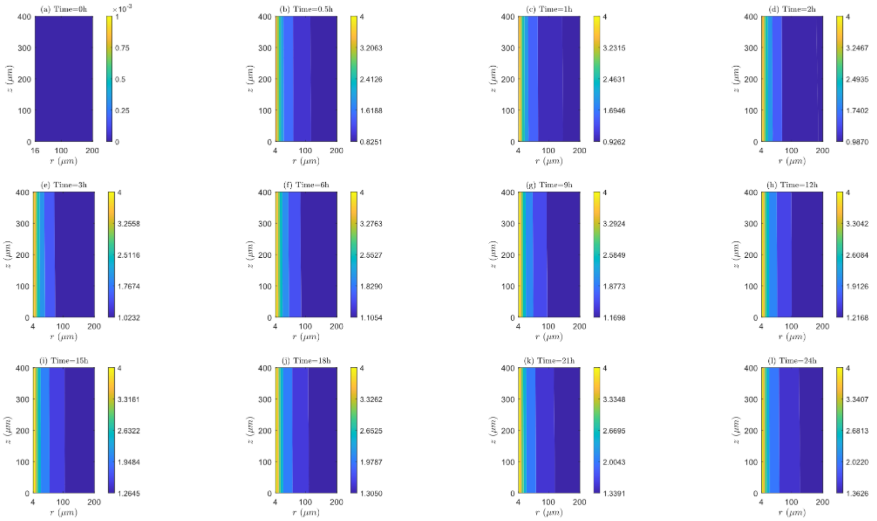

Figure 39. Bound intracellular concentration C_3 - $E = 70kV/m$, $\lambda_{inl} = 0.01m/s$ and UPK.

- ***Algorithm 1: Loading of concentration fields and invocation of main functions.***

```
clear all
close all


% Load of concentration field
% Concentration_field is a data file with information of extracellular, free
intracellular and bound intracellular concentrations at several points and
time instants, with the following outputs:

TIME_TOTAL_ALL: Time instants of evaluation of the concentration fields.

POINTSCOLLOCATION_TOTAL_ALL: Matrix with information of indexing, global
coordinates, and normal vectors (when applied) of boundary and interior points
of the tissue domain at several time instants.


C1_TOTAL_ALL: Matrix with information of extracellular concentration in the
interior and boundary points of the domain.

C2_TOTAL_ALL: Matrix with information of free intracellular concentration in
the interior and boundary points of the domain.

C3_TOTAL_ALL: Matrix with information of bound intracellular concentration in
the interior and boundary points of the domain.

E: Electric field magnitude
dpulses: Pulse spacing
Npulses: Number of continuous pulses
Time_elec: Duration of each electroporation protocol
Nelec: Number of electroporation protocols
id_profile: Parameters of pharmacokinetic profile
lambda: Bloodstream velocity


load('Concentration_field.mat');

% FUNCTION FOR GENERATION OF CONTOURS OF CONCENTRATION RATIOS
[Time,DataX,DataY,Concentration_Ratio,t1,t2]=Data_subplots_generator_Contour_
Rex_int_time_7(POINTSCOLLOCATION_TOTAL_ALL,C1_TOTAL_ALL,C2_TOTAL_ALL,C3_TOTAL
_ALL,E,dpulses,Npulses,Time_elec,Nelec,id_profile,lambda);

% FUNCTION FOR GENERATION OF CONTOURS OF TRANSPORT AND REACTION MECHANISMS
[INDEX,mechanism]=contour_transport_mechanisms_7(E,dpulses,
,Npulses,Time_elec,Nelec,id_profile,lambda,Time,DataX,DataY,Concentration_Rat
io,t1,t2,TIME_TOTAL_ALL,POINTSCOLLOCATION_TOTAL_ALL,C3_TOTAL_ALL,C1_TOTAL_ALL
,C2_TOTAL_ALL);
```

- ***Algorithm 2 (Invoked by Algorithm 1): Generation of contours of concentration ratios.***

```
function

[Time,DataX,DataY,Concentration_Ratio,t1,t2]=Data_subplots_generator_Contour_
Rex_int_time_7(POINTSCOLLOCATION_TOTAL_ALL,C1_TOTAL_ALL,C2_TOTAL_ALL,C3_TOTAL
_ALL,E,dpulses,Npulses,Time_elec,Nelec,id_profile,lambda)

close all


% DISTRIBUTION OF PLOT
number_rows=3;
number_columns=4;

% Variables for plot identification
Letters=["(a) Time=","(b) Time=","(c) Time=","(d) Time=","(e) Time=","(f)
Time=","(g) Time=","(h) Time=","(i) Time=","(j) Time=","(k) Time=","(l)
Time=","(m) Time=","(n) Time=","(o) Time=","(p) Time=","(q) Time=","(r)
Time="];

% Creation of initial contours
for i=2:3
    [COORDR,COORDZ]=meshgrid(16:1:200,0:1:400);
    Rext_int_contour=zeros(size(COORDR));
    figure
    contourf(COORDR,COORDZ,Rext_int_contour,[0,0.0001],'LineStyle','none');
    set(gca,'FontSize',14)
    axis equal
    colormap parula
    cp=colorbar;
    clim([0,1e-3]);
    cp.Label.String = ['$C_',num2str(i),'/C_1$'];%'$C_2/C_1$';
    cp.Label.Interpreter = 'latex';
    cp.Label.FontSize = 18;
    title({'Time=0h'},'FontSize',18,'interpreter','latex')
    xlabel('$r \ (\mu m)$','FontSize',20,'interpreter','latex')
    ylabel('$z \ (\mu m)$','FontSize',20,'interpreter','latex')
    name_ref=['Fig_ref_',num2str(i),'.fig'];
    savefig(name_ref);
    close
end

% Creation of initial contours for bound intracellular concentration
[COORDR,COORDZ]=meshgrid(16:(200-16)/100:200,0:(400-0)/100:400);
C3_contour=zeros(size(COORDR));
figure
contourf(COORDR,COORDZ,C3_contour,[0,0.0001],'LineStyle','none');
set(gca,'FontSize',14)
axis equal
colormap parula
cp=colorbar
clim([0,1e-3]);
cp.Label.String = ['$C_3  (\mu M)$'];%'$C_2/C_1$';
```

```
cp.Label.Interpreter = 'latex'
cp.Label.FontSize = 18;
title({['Time=0h']},'FontSize',18,'interpreter','latex')
xlabel('$r \ (\mu m)$','FontSize',20,'interpreter','latex')
ylabel('$z \ (\mu m)$','FontSize',20,'interpreter','latex')
name_ref=['Fig_ref_',num2str(4),'.fig'];
savefig(name_ref);
close
%---------------------------------------------------------------------------

% Generation of data and contour plots
for l=2:4 % Type of contour
    for i=1:length(E) % Cycle for Electric field magnitude
        for j=1:length(lambda) % Cycle for the blood velocity (lambda)
            for id=1:length(id_profile) % Cycle for the pharmakocinetic
                                          profile (id_profile)
                s=1;
                fnew=figure;
                for k=1:length(Time) % Cycle for the time
                    % Generation of contour plot
                    if s==1
                        name_ref=['Fig_ref_',num2str(l),'.fig'];
                        fig1=openfig(name_ref);
                    else
                        if or(l==2,l==3)
                            Name_identifier=['Contour
                            C',num2str(l),'overC1_',num2str(Time(k)),'_',num2
                            str(E(i)),'_10_0.16667_6_5_24_',num2str(lambda(j)
                            ),'_',num2str(id_profile(id))];
                            fig1 = openfig([Name_identifier,'.fig'],'reuse');
                        else
                            % Obtention of collocation points and
                            concentration
                            POINTSCOLLOCATION(1:length(POINTSCOLLOCATION_TOTA
                            L_ALL),2:3)=POINTSCOLLOCATION_TOTAL_ALL(i,1,1,1,1
                            ,j,id,Time(k)/0.5,1:length(POINTSCOLLOCATION_TOTA
                            L_ALL),1:2);

                            C3(1:length(C3_TOTAL_ALL))=C3_TOTAL_ALL(i,1,1,1,1
                            ,j,id,1:length(C3_TOTAL_ALL),Time(k)/0.5);
                            ymin=min(find(C3==0));
                            if isempty(ymin)==0

                               POINTSCOLLOCATION=POINTSCOLLOCATION(1:ymin-
                               1,1:3);
                                C3=C3(1:ymin-1);
                            end

                            % Calculation of concentration field.
                            limitinfr=min(POINTSCOLLOCATION(:,2));

                            [COORDR,COORDZ]=meshgrid(limitinfr:(200-
                            limitinfr)/15:200,0:(400-0)/15:400);

                            [C3_field]=gen_contours(COORDR,COORDZ,POINTSCOLLO
                            CATION,C3,1);
```

```
        [C3_field]=correction_contour_plots(COORDR,COORDZ
        ,C3_field);

        % Graphic of concentration
        figure

         contourf(COORDR,COORDZ,C3_field,'LineStyle','non
         e');
        fig1=gcf;
        set(gca,'FontSize',14)
        axis equal
        colormap parula
        cp=colorbar
        cp.Label.String = '$C_3  (\mu M)$';
        cp.Label.Interpreter = 'latex'
        cp.Label.FontSize = 18;

        title({['Time=',num2str(Time(k)),'h']},'FontSize'
        ,18,'interpreter','latex')
        xlabel('$r \ (\mu
        m)$','FontSize',20,'interpreter','latex')
        ylabel('$z \ (\mu
        m)$','FontSize',20,'interpreter','latex')
        clear C3 POINTSCOLLOCATION
    end

end
pause(5);
h=gca;
h1=findobj(gca);

 % Extraction of data of contours
[t1(i,j,k),t2(i,j,k)]=size(h1(2).XData);
DataX(i,j,k,1:t1(i,j,k),1:t2(i,j,k))=h1(2).XData;
DataY(i,j,k,1:t1(i,j,k),1:t2(i,j,k))=h1(2).YData;
limit_inf_x=min(min(h1(2).XData));


Concentration_Ratio(l,i,j,id,k,1:t1(i,j,k),1:t2(i,j,k))=h
1(2).ZData;

% Fitting of curves previous to subplot

% Creation of subplot
h_copy = copyobj(h,fnew);
hsub=subplot(number_rows,number_columns,s,h_copy);
close(fig1);

% Change of FontSize
hsub.FontSize=10;

 hsub.Title.String=strcat(Letters(s),num2str(Time(k)),"h"
 );
hsub.Title.Interpreter='latex';
%hsub.Title.Position(2)=-150;
```

```
                    hsub.Title.FontSize=10;
                    colorbar

                    % Fitting of XThick and YThick
                    hsub.XTick=[limit_inf_x,100,200];
                    xtickformat('%.0f');
                    %hsub.YTick=0:0.5e-8:3e-8;
                    h=colorbar;
                    t=get(h,'Limits');
                    set(h,'Ticks',linspace(t(1),t(2),5));
                    s=s+1;
                end
                if or(l==2,l==3)
                       savefig(['Contour
                       C',num2str(l),'overC1_',num2str(E(i)),'_',num2str(lamb
                       da(j)),'_',num2str(id_profile(id)),'.fig']);
                else
                       savefig(['Contour
                       C3_',num2str(E(i)),'_',num2str(lambda(j)),'_',num2str(
                       id_profile(id)),'.fig']);
                end
            end
        end
    end
end

%--------------------------------------------------------------------------
```

- ***Algorithms 3 and 4 (Invoked by Algorithm 2): Generation of interpolated data for contours of concentration ratios***

```
function [Var_field] =
gen_contours(COORDR,COORDZ,POINTSCOLLOCATION,Var,negative_correction)

% This function generates data for the contour of the field variable

[t1,t2]=size(COORDR);
for i=1:t1
    for j=1:t2
         [y1,y2]=sort(sqrt((POINTSCOLLOCATION(:,2)-
         COORDR(i,j)).^2+(POINTSCOLLOCATION(:,3)-COORDZ(i,j)).^2),'ascend');
         if
             or(or(and(i==t1,j==1),and(i==t1,j==t2)),or(and(i==1,j==1),and(i=
             =1,j==t2)))
                   syms r z y
             M=[r-POINTSCOLLOCATION(y2(1),2),z-POINTSCOLLOCATION(y2(1),3),y-
             Var(y2(1));POINTSCOLLOCATION(y2(2),2)-
             POINTSCOLLOCATION(y2(1),2),POINTSCOLLOCATION(y2(2),3)-
             POINTSCOLLOCATION(y2(1),3),Var(y2(2))-Var(y2(1));...
             POINTSCOLLOCATION(y2(3),2)-
             POINTSCOLLOCATION(y2(1),2),POINTSCOLLOCATION(y2(3),3)-
             POINTSCOLLOCATION(y2(1),3),Var(y2(3))-Var(y2(1))];
             eqn=subs(subs(det(M),r,COORDR(i,j)),z,COORDZ(i,j))==0;
             Var_field(i,j)=double(solve(eqn));

        else
            Var_field(i,j)=(Var(y2(1)));
        end

        % Correction of small negative values
        if negative_correction==1
            if Var_field(i,j)<0
                Var_field(i,j)=0;
            end
        end
    end
end

%-------------------------------------------------------------------------

function [Var_field]=correction_contour_plots(COORDR,COORDZ,Var_field)

% This function corrects contour plots using linear RBF interpolation

% Size of field
[t1,t2]=size(COORDR);

% Detection of Infinite and Undetermined values in Var_field
[yinf1,yinf2]=find(isinf(Var_field)==1);
[ynan1,ynan2]=find(isnan(Var_field)==1);
y1=[yinf1,ynan1];
y2=[yinf2,ynan2];
```

```
% Cycle for calculation of correction points
for i=1:length(y1)

 % Vector of problematic positions
 posrow=[];
 poscol=[];

 % Point 1
 if y2(i)>1
    posrow=[posrow,y1(i)];
    poscol=[poscol,y2(i)-1];
 end

 % Point 2
 if y2(i)<t2
    posrow=[posrow,y1(i)];
    poscol=[poscol,y2(i)+1];
 end

 if y1(i)>1
     % Point 3
     posrow=[posrow,y1(i)-1];
     poscol=[poscol,y2(i)];
     if y2(i)>1
         % Point 4
         posrow=[posrow,y1(i)-1];
         poscol=[poscol,y2(i)-1];
     end
     if y2(i)<t2
         % Point 5
         posrow=[posrow,y1(i)-1];
         poscol=[poscol,y2(i)+1];
     end
 end

 if y1(i)<t2
     % Point 6
     posrow=[posrow,y1(i)+1];
     poscol=[poscol,y2(i)];
     if y2(i)>1
         % Point 7
         posrow=[posrow,y1(i)+1];
         poscol=[poscol,y2(i)-1];
     end
     if y2(i)<t2
         % Point 8
         posrow=[posrow,y1(i)+1];
         poscol=[poscol,y2(i)+1];
     end
 end

 % Cycle for filtering of data
 for j=1:length(posrow)
     if
        or(isnan(Var_field(posrow(j),poscol(j)))==1,isinf(Var_field(posrow(j)
        ,poscol(j)))==1)
```

```
            posrow(j)=0;
            poscol(j)=0;
        end
    end
    posrow(find(posrow==0))=[];
    poscol(find(poscol==0))=[];

    % Cycle for calculation of RBF interpolation
    for j=1:length(posrow)
        for k=j+1:length(posrow)
             R(j,k)=sqrt((COORDR(posrow(j),poscol(j))-
             COORDR(posrow(k),poscol(k))).^2+...
                                    (COORDZ(posrow(j),poscol(j))-
             COORDZ(posrow(k),poscol(k))).^2);
        end
        V(j,1)=Var_field(posrow(j),poscol(j));
        R(j+1:length(posrow),j)=R(j,j+1:length(posrow))';
        A(1,j)=sqrt((COORDR(posrow(j),poscol(j))-COORDR(y1(i),y2(i))).^2+...
       (COORDZ(posrow(j),poscol(j))-COORDZ(y1(i),y2(i))).^2);
    end

    Var_field(y1(i),y2(i))=A*(inv(R)*V);

    clear R V A
end

%--------------------------------------------------------------------------
```

- ***Algorithms 5 (Invoked by Algorithm 1): Generation of contours of reaction and transport mechanisms at several time instants.***

```
function [INDEX,mechanism]=contour_transport_mechanisms_7(E,dpulses,
,Npulses,Time_elec,Nelec,id_profile,lambda,Time,DataX,DataY,Concentration_Rat
io,t1,t2,TIME_TOTAL_ALL,POINTSCOLLOCATION_TOTAL_ALL,C3_TOTAL_ALL,C1_TOTAL_ALL
,C2_TOTAL_ALL)

close all

% CONTOURS OF TRANSPORT MECHANISM
Letters=["(a) ","(b) ","(c) ","(d) ","(e) ","(f) ","(g) ","(h) ","(i) ","(j)
","(k) ","(l) ","(m) ","(n) ","(o) ","(p) ","(q) ","(r) "];
for i=1:length(E) % Cycle for Electric field magnitude
   for j=1:length(lambda) % Cycle for the pulse spacing (dpulses)
        for id=1:length(id_profile)
            s=1;
            fnew=figure;
            incre_x=0;
            incre_y=0;

            for k=1:length(Time)-1 % Cycle for the time
                TimeAux(1:48)=TIME_TOTAL_ALL(i,1,1,1,1,j,id,1:48)/3600;
                [y1to]=find(Time(k)==TimeAux);
                if isempty(y1to)==1
                   y1to=1;
                   PointsCollocationto(1:length(POINTSCOLLOCATION_TOTAL_ALL),
                   1:2)=POINTSCOLLOCATION_TOTAL_ALL(i,1,1,1,1,j,id,y1to,1:len
                   gth(POINTSCOLLOCATION_TOTAL_ALL),1:2);
                   Concentration1to(1:length(C1_TOTAL_ALL))=0;
                   Concentration3to(1:length(C3_TOTAL_ALL))=0;
                else

                     PointsCollocationto(1:length(POINTSCOLLOCATION_TOTAL_ALL
                     ),1:2)=POINTSCOLLOCATION_TOTAL_ALL(i,1,1,1,1,j,id,y1to,1
                     :length(POINTSCOLLOCATION_TOTAL_ALL),1:2);

                     Concentration1to(1:length(C1_TOTAL_ALL))=C1_TOTAL_ALL(i,
                     1,1,1,1,j,id,1:length(C1_TOTAL_ALL),y1to);

                     Concentration3to(1:length(C3_TOTAL_ALL))=C3_TOTAL_ALL(i,
                     1,1,1,1,j,id,1:length(C3_TOTAL_ALL),y1to);
                end

                % For time step tf
                [y1tf]=find(Time(k+1)==TimeAux);
                if isempty(y1tf)==1
                    y1tf=1;
                    PointsCollocationtf(1:length(POINTSCOLLOCATION_TOTAL_ALL)
                    ,1:2)=POINTSCOLLOCATION_TOTAL_ALL(i,1,1,1,1,j,id,y1tf,1:l
                    ength(POINTSCOLLOCATION_TOTAL_ALL),1:2);
                    Concentration1tf(1:length(C1_TOTAL_ALL))=0;
                    Concentration3tf(1:length(C3_TOTAL_ALL))=0;
                else
```

```
    PointsCollocationtf(1:length(POINTSCOLLOCATION_TOTAL_ALL)
    ,1:2)=POINTSCOLLOCATION_TOTAL_ALL(i,1,1,1,1,1,j,id,y1tf,1:l
    ength(POINTSCOLLOCATION_TOTAL_ALL),1:2);

    Concentration1tf(1:length(C1_TOTAL_ALL))=C1_TOTAL_ALL(i,1
    ,1,1,1,j,id,1:length(C1_TOTAL_ALL),y1tf);

    Concentration3tf(1:length(C3_TOTAL_ALL))=C3_TOTAL_ALL(i,1
    ,1,1,1,j,id,1:length(C3_TOTAL_ALL),y1tf);
end

 % Obtention of contour of concentration ratios

X_Data(1:t1(i,j,k),1:t2(i,j,k))=DataX(i,j,k,1:t1(i,j,k),1:t2
(i,j,k));

Y_Data(1:t1(i,j,k),1:t2(i,j,k))=DataY(i,j,k,1:t1(i,j,k),1:t2
(i,j,k));

C2_over_C1_to(1:t1(i,j,k),1:t2(i,j,k))=Concentration_Ratio(2
,i,j,id,k,1:t1(i,j,k),1:t2(i,j,k));

C2_over_C1_tf(1:t1(i,j,k),1:t2(i,j,k))=Concentration_Ratio(2
,i,j,id,k+1,1:t1(i,j,k),1:t2(i,j,k));

C3_over_C1_to(1:t1(i,j,k),1:t2(i,j,k))=Concentration_Ratio(3
,i,j,id,k,1:t1(i,j,k),1:t2(i,j,k));

C3_over_C1_tf(1:t1(i,j,k),1:t2(i,j,k))=Concentration_Ratio(3
,i,j,id,k+1,1:t1(i,j,k),1:t2(i,j,k));

% Plot of contour of mechanism
[INDEX,Index_Diff,mechanism,colorin,CHANGE_EXTRA]=generation
_contour_mechanisms_7_mod(X_Data,Y_Data,C2_over_C1_to,C2_ove
r_C1_tf,C3_over_C1_to,C3_over_C1_tf,PointsCollocationto,Conc
entration3to,PointsCollocationtf,Concentration3tf,Concentrat
ion1to,Concentration1tf);

% Plot of contour of mechanism
hsub(s)=subplot(3,4,s);
surf(X_Data,Y_Data,INDEX,'EdgeColor','none','FaceAlpha',0.8)
hold on
[p1,p2]=find(CHANGE_EXTRA==1);
plot(X_Data(p1,p2),Y_Data(p1,p2),'.k','MarkerSize',4);
view([0 90]);
axis equal
axis([min(min(X_Data)) max(max(X_Data)) min(min(Y_Data))
max(max(Y_Data))]);
colormap(hsub(s),colorin);

for m=1:length(Index_Diff)
     annotation('textbox', [0.238+incre_x 0.863-incre_y 0.85
     0.05],
     'String',mechanism(m),'FitBoxToText','on','BackgroundCol
     or',colorin(m,1:3),'FontSize',8)
```

```
            end
            hsub(s).XTick=[min(min(X_Data)), 100, 200]
            xtickformat('%.0f');
            set(gca,'FontSize',8)
            title(strcat(Letters(s),num2str(Time(k)),"h to
            t=",num2str(Time(k+1)),"h"),'FontSize',10,'interpreter','lat
            ex')
            xlabel('$r \ (\mu m)$','FontSize',10,'interpreter','latex')
            ylabel('$z \ (\mu m)$','FontSize',10,'interpreter','latex')
            s=s+1;
            hold on
            incre_x=incre_x+0.207;
            if mod(k,4)==0
                incre_y=incre_y+0.29;
                incre_x=0;
            end

            clear TimeAux PointsCollocationto Concentration3to
            PointsCollocationtf Concentration3tf X_Data Y_Data
            C2_over_C1_to C2_over_C1_tf C3_over_C1_to C3_over_C1_tf

        end
        savefig(['Contour Transport
        Mechanisms',num2str(E(i)),'_',num2str(lambda(j)),'_',num2str(id_
        profile(id)),'.fig'
    end
  end
end
%------------------------------------------------------------------------
```

- ***Algorithms 6 (Invoked by Algorithm 5): Application of Boolean model.***

```
function
[INDEX,Index_Diff,mechanism,colorin,CHANGE_EXTRA]=generation_contour_mechanis
ms_7_mod(X_Data,Y_Data,C2_over_C1_to,C2_over_C1_tf,C3_over_C1_to,C3_over_C1_t
f,PointsCollocationto,Concentration3to,PointsCollocationtf,Concentration3tf,C
oncentration1to,Concentration1tf)

% Generation of initial logic vector and matrices
[t1,t2]=size(X_Data);
COMPARISON(1)="(IN≥AS)∨(IN≥ECT+)";
COMPARISON(2)="EX≤ECT-";
COMPARISON(3)="∃AS ∧ ∃IN";
COMPARISON(4)="(EX≤ECT-)∧(EX≥DIS)";
COMPARISON(5)="IN≥AS";
COMPARISON(6)="IN≥ECT+";
COMPARISON(7)="(IN≥AS)⇔(IN≤ECT+)";
COMPARISON(8)="IN≤ECT+";
COMPARISON(9)="(EX≤DIS)⇔(EX≥ECT-)";
COMPARISON(10)="(IN≥AS)∧(IN≤ECT+)";
COMPARISON(11)="(EX≤DIS)∨(EX≤ECT-)";
COMPARISON(12)="(IN≤AS)∨(IN≤ECT+)";
COMPARISON(13)="(EX≥DIS)∨(EX≥ECT-)";
COMPARISON(14)="∃AS ∧ ∄EX ∧ ∄IN";
COMPARISON(15)="∃DIS ∧ ∃IN";
COMPARISON(16)="∃DIS ∧ ∄EX ∧ ∄IN";
COMPARISON(17)="∃AS ∧ ∃EX";

% Assignment of colors for each transport and reaction mechanism
colores=[1 0 0; 0 1 0; 0 0 1; 0 1 1;1 0 1; 1 1 0;0 0.45 0.75;0.85 0.3 0.1;...
    0.9 0.7 0.12; 0.50 0.20 0.55; 0.45 0.65 0.2; 0.30 0.75 0.9; 0.65 0.1
    0.2;0.35 0.9 0.8; 0.70 0.25 0.1; 0.55 0.35 0.8; 0.50 0.80 0.45];

% Creation od CHANGE_EXTRA
CHANGE_EXTRA=zeros(t1,t2);

for i=1:t1
    for j=1:t2
        % Determination whether there is Drug Association or Dissociation
                between time instants
          [y1to,y2to]=min(sqrt((X_Data(i,j)-
          PointsCollocationto(:,1)).^2+(Y_Data(i,j)-
          PointsCollocationto(:,2)).^2));
          [y1tf,y2tf]=min(sqrt((X_Data(i,j)-
          PointsCollocationtf(:,1)).^2+(Y_Data(i,j)-
          PointsCollocationtf(:,2)).^2));

        if Concentration3to(y2to)<=Concentration3tf(y2tf)
           AS=1; % There is Association
        else
            AS=0; % There is Dissociation
        end

        % Determination of change of extracellular concentration
```

```
if Concentration1to(y2to)>=Concentration1tf(y2tf) % Extracellular
concentration increases
       CHANGE_EXTRA(i,j)=1;
end
%-------------------------------------------------------------------

% Determination whether there is Internalization or Externalization
% between time instants
if and(C2_over_C1_to(i,j)<1,C2_over_C1_tf(i,j)<1)
    INTER_EXTER=1; % There is internalization at both time instants
else if and(C2_over_C1_to(i,j)>1,C2_over_C1_tf(i,j)>1)
        INTER_EXTER=2;  % There is externalization at both time
        instants
else if and(C2_over_C1_to(i,j)<1,C2_over_C1_tf(i,j)>1)
        INTER_EXTER=3; % Internalization at to and Externalization
        at tf
else if and(C2_over_C1_to(i,j)>1,C2_over_C1_tf(i,j)<1)
        INTER_EXTER=4; % Externalization at to and Internalization
        at tf
else if and(C2_over_C1_to(i,j)==1,C2_over_C1_tf(i,j)==1)
        INTER_EXTER=5; % Neither internalization, nor
        externalization is present at both time
end
end
end
end
end
%-------------------------------------------------------------------

% Determination of the transport mechanisms
if C2_over_C1_to(i,j)<=C2_over_C1_tf(i,j)
    if C3_over_C1_to(i,j)<=C3_over_C1_tf(i,j)
        if
        (C3_over_C1_to(i,j)/C2_over_C1_to(i,j))<=(C3_over_C1_tf(i,j)
        /C2_over_C1_tf(i,j))
         %----------------------------------------------------------
         if AS==1 % There is association
            if INTER_EXTER==1
                INDEX(i,j)=1; % Internalization at both times
            else if INTER_EXTER==2
                    INDEX(i,j)=2; % Externalization at both times
            else if INTER_EXTER==5
                     INDEX(i,j)=14; % Neither externalization, nor
                     internalization
                else
                     INDEX(i,j)=120;
                end
                end
                end
            else % There is Dissociation
                if INTER_EXTER==1
                    INDEX(i,j)=15;
                else if INTER_EXTER==2
                        INDEX(i,j)=4; % Externalization at both times
                else if INTER_EXTER==5
                        INDEX(i,j)=16;
```

```
            else
                 INDEX(i,j)=4150;
            end
            end
            end
        end
        %-------------------------------------------------------
    else

        if AS==1 % There is Association
            if INTER_EXTER==1
                INDEX(i,j)=5; % There is internalization
            else if INTER_EXTER==2
                    INDEX(i,j)=17;
            else if INTER_EXTER==5
                    INDEX(i,j)=14;
            else
                    INDEX(i,j)=5170;
            end
            end
            end

        else % There is Dissociation
            if INTER_EXTER==1
                INDEX(i,j)=6; % Internalization at both times
            else if INTER_EXTER==2
                    INDEX(i,j)=2; % Externalization at both times
            else if INTER_EXTER==5
                   INDEX(i,j)=16; % Neither internalization, nor
                 externalization
            else
                INDEX(i,j)=620; % Internalization+Externalization
            end
            end
            end
        end
        %-------------------------------------------------------

    end

else

     if
     (C3_over_C1_to(i,j)/C2_over_C1_to(i,j))<=(C3_over_C1_tf(i,j)
     /C2_over_C1_tf(i,j))

    %-------------------------------------------------------
    if AS==1 % There is Association
            if INTER_EXTER==1
                 INDEX(i,j)=7; % Internalization at both time
                 instants
            else if INTER_EXTER==2
                 INDEX(i,j)=2; % Externalization at both time
                 instants
            else if INTER_EXTER==5
```

```
                INDEX(i,j)=14;  % Neither externalization,
              nor internalization
        else
            INDEX(i,j)=720; % Internalization+Externalization
        end
        end
        end
    else % There is Dissociation
        if INTER_EXTER==1
              INDEX(i,j)=8; % Internalization at both time
              instants
        else if INTER_EXTER==2
                INDEX(i,j)=9; % Externalization at both time
              instants
        else if INTER_EXTER==5
                INDEX(i,j)=16;
        else
              INDEX(i,j)=890; % Internalization +
              Externalization
        end
        end
        end
    end
    %-----------------------------------------------------
else
    if AS==1 % There is Association
        if INTER_EXTER==1
              INDEX(i,j)=10; % Internalization at both time
              instants
        else if INTER_EXTER==2
                 INDEX(i,j)=17;
        else if INTER_EXTER==5
                INDEX(i,j)=14;
        else
            INDEX(i,j)=10170;
        end
        end
        end
    else % There is Dissociation
        if INTER_EXTER==1
            INDEX(i,j)=15; % Internalization at both time
                           instants
        else if INTER_EXTER==2
                INDEX(i,j)=11; % Externalization at both time
              instants
        else if INTER_EXTER==5
                INDEX(i,j)=16; % Neither internalization, nor
              externalization
        else
              INDEX(i,j)=15110; %
              Internalization+Externalization
        end
        end
        end
    end
    %-----------------------------------------------------
```

```
        end

    end
else

    if C3_over_C1_to(i,j)<=C3_over_C1_tf(i,j)

        if
        (C3_over_C1_to(i,j)/C2_over_C1_to(i,j))<=(C3_over_C1_tf(i,j)/
        C2_over_C1_tf(i,j))

            if AS==1 % There is Association
                if INTER_EXTER==1
                     INDEX(i,j)=12; % Internalization at both time
                     instants
                else if INTER_EXTER==2
                       INDEX(i,j)=17;  % Externalization at both
                     time instants
                else if INTER_EXTER==5  % Neither externalization,
                nor internalization
                        INDEX(i,j)=14;
                else
                    INDEX(i,j)=12170; % Internalization +
               Externalization
                end
                end
                end
            else  % There is Dissociation
                if INTER_EXTER==1
                    INDEX(i,j)=15;
                else if INTER_EXTER==2
                       INDEX(i,j)=4; % Externalization at both time
                     instants
                else if INTER_EXTER==5
                        INDEX(i,j)=16;
                else
                    INDEX(i,j)=4150;
                end
                end
                end
            end

        else

            if AS==1 % There is Association
                if INTER_EXTER==1
                     INDEX(i,j)=7; % Internalization at both time
                     instants
                else if INTER_EXTER==2
                        INDEX(i,j)=2; % Externalization at both time
                     instants
                else if INTER_EXTER==5
                        INDEX(i,j)=14; % Neither internalization,
                     nor externalization
                else
```

```
                    INDEX(i,j)=720; % Internalization +
                    Externalization
                end
                end
                end
            else  % There is Dissociation
                if INTER_EXTER==1
                    INDEX(i,j)=8; % Internalization at both time
                    instants
                else if INTER_EXTER==2
                      INDEX(i,j)=9; % Externalization at both time
                    instants
                else if INTER_EXTER==5
                       INDEX(i,j)=16;
                else
                    INDEX(i,j)=890; % Internalization +
                    Externalization
                end
                end
                end
            end
            %-------------------------------------------------------

        end

    else

        if
        (C3_over_C1_to(i,j)/C2_over_C1_to(i,j))<=(C3_over_C1_tf(i,j)
        /C2_over_C1_tf(i,j))

            if AS==1 % There is Association
                if INTER_EXTER==1
                    INDEX(i,j)=12; % Internalization at both time
                    instants
                else if INTER_EXTER==2
                      INDEX(i,j)=17; % Externalization at both time
                    instants
                else if INTER_EXTER==5
                      INDEX(i,j)=14; % Neither internalization, nor
                    externalization
                else
                    INDEX(i,j)=12170; % Internalization +
                    Externlization
                end
                end
                end
            else % There is dissociation
                if INTER_EXTER==1
                    INDEX(i,j)=8; % Internalization at both time
                  instants
                else if INTER_EXTER==2
                       INDEX(i,j)=13; % Externalization at both
                    time instants
                else if INTER_EXTER==5
                       INDEX(i,j)=16;
```

```
                        else
                             INDEX(i,j)=8130; % Internalization +
                             Externalization
                        end
                        end
                        end
                    end
                    %-------------------------------------------------------
                else

                    if AS==1 % There is Association
                        if INTER_EXTER==1
                             INDEX(i,j)=10; % Internalization at both time
                             instants
                        else if INTER_EXTER==2
                               INDEX(i,j)=17;
                        else if INTER_EXTER==5
                               INDEX(i,j)=14;
                        else
                            INDEX(i,j)=10170;
                        end
                        end
                        end

                    else % There is Dissociation
                        if INTER_EXTER==1
                             INDEX(i,j)=8; % Internalization at both time
                             instants
                        else if INTER_EXTER==2
                              INDEX(i,j)=13; % Externalization at both time
                             instants
                        else if INTER_EXTER==5
                               INDEX(i,j)=16;
                        else
                             INDEX(i,j)=8130; % Internalization +
                             Externalization
                        end
                        end
                        end
                    end
                    %------------------------------------------------------
                end
            end
        end
    end

end
%--------------------------------------------------------------------------

% CORRECTION OF MATRIX OF INDEX
[y1,y2]=find(INDEX==0);
if isempty(y1)==0
    stop=1;
end

% GENERATION OF CONTOUR PLOT
```

```
% Obtention of different indexes in the matrix
Index_Diff=unique(INDEX);
colorin=[];
mechanism=[];
for i=1:length(Index_Diff)
    if Index_Diff(i)<100 % The index does not end at zero
        colorin=[colorin;colores(Index_Diff(i),1:3)];
        mechanism=[mechanism;COMPARISON(Index_Diff(i))];
     else
        a=num2str(Index_Diff(i)/10);
        if length(a)==2

          colorin=[colorin;(colores(str2num(a(1)),1:3)+colores(str2num(a(2)),
          1:3))/2];
            mechanism=[mechanism;strcat(COMPARISON(str2num(a(1))),"-to-
          ",COMPARISON(str2num(a(2))))];
        else if length(a)==3

                 colorin=[colorin;(colores(str2num(a(1)),1:3)+colores(str2num
                 (a(2:3)),1:3))/2];
                  mechanism=[mechanism;strcat(COMPARISON(str2num(a(1))),"-to-
                 ",COMPARISON(str2num(a(2:3))))];
              else

              colorin=[colorin;(colores(str2num(a(1:2)),1:3)+colores(str2num(
              a(3:4)),1:3))/2];
              mechanism=[mechanism;strcat(COMPARISON(str2num(a(1:2))),"-to-
              ",COMPARISON(str2num(a(3:4))))];
             end
        end
    end
end
%------------------------------------------------------------------------
```
